# Repeated loss of the West Antarctic Ice Sheet’s Amundsen Sea sector during late Pleistocene interglacials

**DOI:** 10.1038/s41467-026-77004-2

**Published:** 2026-11-09

**Authors:** Claus-Dieter Hillenbrand, Werner Ehrmann, Young Kyu Park, Jinwook Kim, Steve M. Bohaty, Reed P. Scherer, Joseph A. Ruggiero, Julia S. Wellner, Karsten Gohl, Rebecca L. Totten, Robert D. Larter, Mariana Esteves, Johann P. Klages, James A. Smith, Thomas Frederichs, Keiji Horikawa, Sandra Passchier, Joseph R. Mastro, Thorsten Bauersachs, Becky A. Hopkins, Chuang Xuan, Colm Ó Cofaigh, Rachel W. Clark, James W. Marschalek, Patric Simões Pereira, Benedict T. I. Reinardy, Anna Ruth W. Halberstadt, Li Wu, Ellen A. Cowan, Christine S. Siddoway, Delaney E. Robinson, Masao Iwai, Liang Gao, Sidney R. Hemming, Michelle L. Penkrot, Theresa M. King, Jeremy M. Lloyd, Denise K. Kulhanek

**Affiliations:** 1 British Antarctic Survey, Cambridge, UK; 2 Institute for Earth System Science and Remote Sensing, University of Leipzig, Leipzig, Germany; 3 Department of Earth System Sciences, Yonsei University, Seoul, Republic of Korea; 4 Korea Polar Research Institute, Incheon, Republic of Korea; 5 Institute of Earth Sciences, Heidelberg University, Heidelberg, Germany; 6 Department of Earth, Atmosphere and Environment, Northern Illinois University, DeKalb, IL USA; 7 School of Earth, Space and Planets, Colorado School of Mines, Golden, CO USA; 8 Department of Earth and Atmospheric Sciences, University of Houston, Houston, TX USA; 9 Alfred Wegener Institute Helmholtz Centre for Polar and Marine Research, Bremerhaven, Germany; 10 Faculty of Geosciences, University of Bremen, Bremen, Germany; 11 Department of Geological Sciences, The University of Alabama, Tuscaloosa, AL USA; 12 CONSERVE Center, Alabama Water Institute, The University of Alabama, Tuscaloosa, AL USA; 13 Department of Geosciences, UiT the Arctic University of Norway, Tromsø, Norway; 14 Cluster of Excellence “The Ocean Floor – Earth’s Uncharted Interface”, University of Bremen, Bremen, Germany; 15 MARUM – Center for Marine Environmental Sciences, University of Bremen, Bremen, Germany; 16 Faculty of Science, Academic Assembly, University of Toyama, Toyama, Japan; 17 Department of Earth and Environmental Studies, Montclair State University, Montclair, NJ USA; 18 Chair of Organic Biogeochemistry in Geo-Systems, RWTH Aachen University, Aachen, Germany; 19 School of Ocean and Earth Science, University of Southampton, Southampton, UK; 20 Department of Earth, Ocean and Ecological Sciences, University of Liverpool, Liverpool, UK; 21 Department of Geography, Durham University, Durham, UK; 22 Department of Earth Science and Engineering, Imperial College London, London, UK; 23 Institute of Geology, University of Innsbruck, Innsbruck, Austria; 24 Department of Sustainable Development, Environmental Science and Engineering, KTH Royal Institute of Technology, Stockholm, Sweden; 25 Department of Earth and Planetary Sciences, Jackson School of Geosciences, The University of Texas at Austin, Austin, TX USA; 26 Laboratory for Coastal Ocean Variation and Disaster Prediction, College of Oceanography and Meteorology, Guangdong Ocean University, Zhanjiang, China; 27 State Key Laboratory of Marine Geology, Tongji University, Shanghai, China; 28 Department of Geological and Environmental Sciences, Appalachian State University, Boone, NC USA; 29 Department of Geology, Colorado College, Colorado Springs, CO USA; 30 Kochi Core Center (KCC), Marine Core Research Institute (MaCRI), Kochi University, Nankoku, Japan; 31 Key Laboratory of Polar Geology and Marine Mineral Resources, China University of Geosciences, Beijing, China; 32 School of Ocean Sciences, China University of Geosciences, Beijing, China; 33 Department of Earth and Environmental Sciences and Lamont-Doherty Earth Observatory of Columbia University, Palisades, NY USA; 34 Scientific Ocean Drilling, Texas A&M University, College Station, TX USA; 35 College of Marine Science, University of South Florida, St. Petersburg, FL USA; 36 Institute of Geosciences, Christian-Albrechts-University Kiel, Kiel, Germany

**Keywords:** Palaeoclimate, Palaeoceanography

## Abstract

Sea-level reconstructions and numerical models suggest that the vulnerable West Antarctic Ice Sheet underwent major mass losses during the late Pleistocene, raising global sea level by several metres. However, direct evidence from ice-sheet proximal records for such retreat has remained inconclusive. Here we investigate variations in clay mineral assemblages and past ocean temperatures archived in West Antarctic continental margin sediments during the last 800,000 years. Our results demonstrate that the supply of glacigenic detritus sourced from the main outlet glaciers in the Amundsen Sea sector of the West Antarctic Ice Sheet ceased almost entirely during all previous warm periods, documenting the loss of their drainage basins. Concomitant seawater warming, revealed by a microfossil proxy, is attributed to intensified deep-water advection towards Antarctica’s Pacific margin, which is corroborated by the clay mineralogical evidence. Our findings show that ocean forcing can drive major deglaciation of West Antarctica and related global sea-level rise.

## Abstract (web summary)

The mineralogical composition of the fine-grained fraction of Antarctic continental margin sediments reveals major retreat of glaciers draining the Amundsen Sea sector of the West Antarctic Ice Sheet during each warm period of the last 800,000 years.

## Introduction

**Fig. 1:**
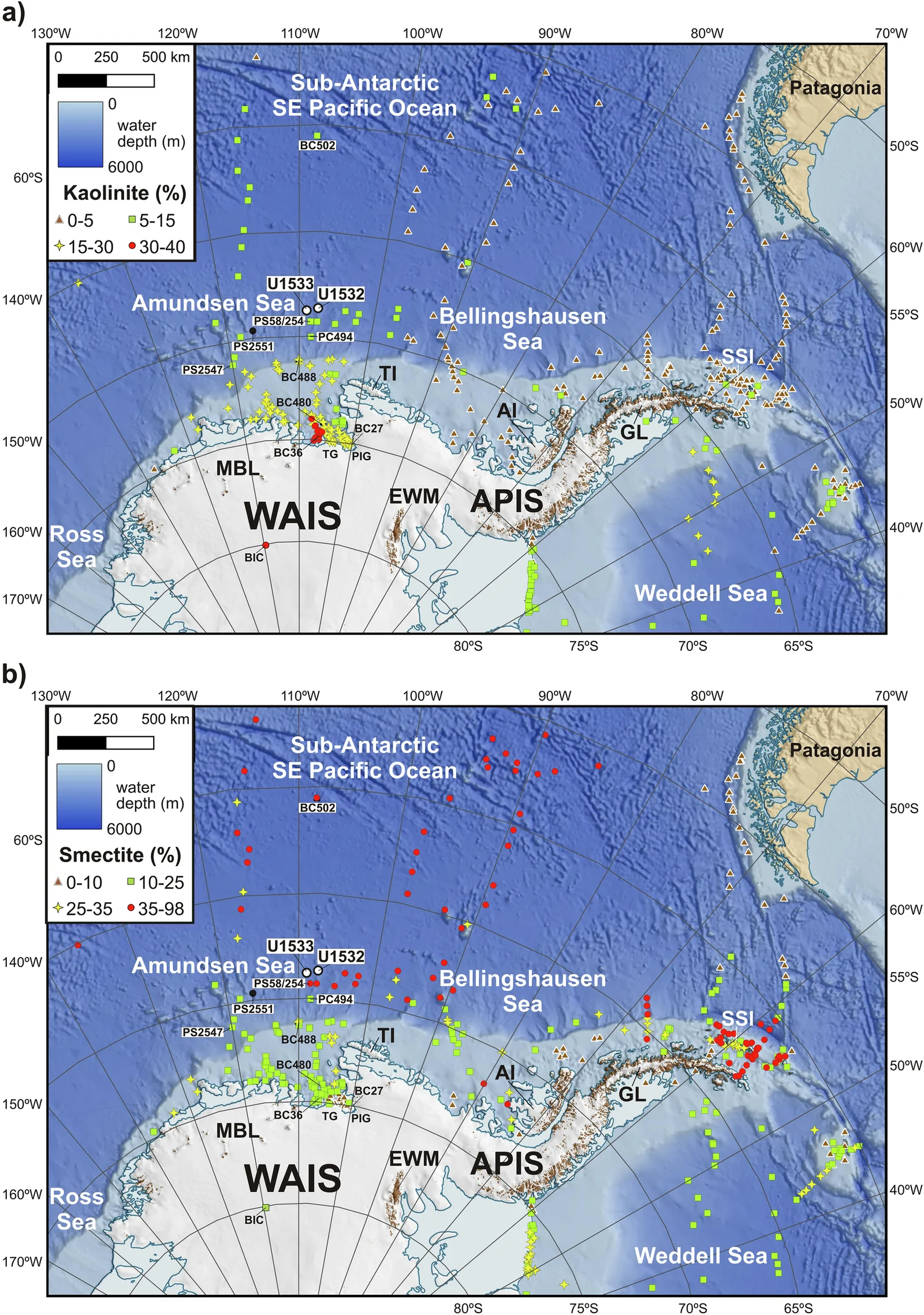
Kaolinite and smectite contents in contemporary sediments from the SE Pacific and SW Atlantic sectors of the Southern Ocean. **a** Bathymetric map showing kaolinite content in the clay fraction (<2 μm) of latest MIS-1 to modern seafloor-surface sediments and basal glacigenic debris of the Byrd Ice Core (BIC). Clay mineral data from refs. ^18,26,27,29–31,33,73^, this study, and additional sources (Supplementary Data S2). Locations of sediment cores analysed for this study (U1533, U1532) and referred to in the text (PS2547^18^; PS2551^18^; PS58/254^37^; PC494^75^) and Fig. 2 are also shown. Map generated with Quantarctica^86^, using data from IBCSO v.1.0^87^ and SCAR ADD v.7.0^88^. AI Alexander Island, APIS Antarctic Peninsula Ice Sheet, EWM Ellsworth-Whitmore Mountains, GL Graham Land, MBL Marie Byrd Land, PIG Pine Island Glacier, SSI South Shetland Islands, TG Thwaites Glacier, TI Thurston Island, WAIS West Antarctic Ice Sheet. **b** Same as in **a** but for smectite content.

About 80% of the West Antarctic Ice Sheet (WAIS) rests below sea level on a landward dipping bed^1^, making it Earth’s ice sheet that is most vulnerable to rapid retreat. Modern observations have revealed that ice-shelf melting along the WAIS’s Pacific margin is driven by intrusions of warm Circumpolar Deep Water (CDW) onto the continental shelf^2^. The melting causes thinning of the ice shelves, weakening their buttressing effect, which increases the flow of grounded ice feeding into them. This, in turn, initiates glacier thinning upstream of the ice shelves and grounding-line retreat^2–4^. As a result, the WAIS is currently losing mass at an accelerating rate^3^^,^^4^. Between 2003 and 2019 Common Era (C.E.), WAIS retreat and thinning contributed ~7.5 mm to global sea-level rise^4^, and numerical models predict a likely Antarctic contribution of 8‒24 cm by 2100 C.E., depending on future greenhouse gas emission scenarios and associated warming^5^. Present WAIS melting is focused in the Amundsen Sea sector^2–4^, where Thwaites and Pine Island glaciers (Fig. 1) drain one third of the WAIS into the eastern Amundsen Sea Embayment. Potentially irreversible ice loss from these two major outlets may initiate not only widespread deglaciation in the Amundsen Sea drainage sector but also other marine-based WAIS sectors and even the entire WAIS within the coming centuries^6,7^. This would raise global sea-level by 3.3‒5.3 m^1^.

Both far-field records of past global sea-level highstands^8–10^ and coupled ice-sheet climate model results^5,11–13^ suggest that partial or even total WAIS “collapse”—i.e., major, rapid retreat—occurred during some warm periods of the Brunhes chron (773–0 ka). This includes the last interglacial peak of Marine Isotope Stage (MIS) 5e (130–115 ka), which is regarded as a key analogue for a near-future scenario with global mean annual temperatures exceeding pre-industrial levels by ~1–1.5 °C for decades and most likely even longer. Identifying the exact magnitude, timing, and source ice sheet(s) of past sea-level maxima from far-field records, however, remains challenging^8,14–16^. Moreover, different ice-sheet model simulations disagree regarding the extent of WAIS retreat during past warm periods, and which of the WAIS drainage basins, if any, deglaciated^5,11–13^. Also, direct evidence for late Pleistocene WAIS loss from subglacial records^17^, continental margin sediments^18,19^, or genetic data^20^ has, so far, been inconclusive or disputed (Supplementary Information). Furthermore, several Antarctic geological records have shown that various WAIS drainage sectors experienced episodes of temporary ice-sheet retreat and thinning beyond modern limits, followed by re-advance and re-thickening during the Holocene (11.7–0 ka)^21^. These findings may challenge the view of the inevitable irreversibility of the present WAIS retreat.

Given all these uncertainties, knowledge of WAIS response to natural climatic changes during the last 800 kyr, when glacial-interglacial cycles followed a 100-kyr periodicity^22^, is essential for (1) assessing the reliability of model-based sea-level projections, and (2) understanding whether current WAIS retreat is likely to continue. Here we investigate clay mineral assemblages in sedimentary records drilled at International Ocean Discovery Program (IODP) Expedition 379 sites U1533 and U1532 on the eastern Amundsen Sea continental rise^23^ to reconstruct changes in WAIS extent since 800 ka. Along with a seawater temperature proxy based on morphological variations of diatom frustules, we use these data to identify the possible driver(s) of past WAIS retreat. The main objective of our study is to evaluate whether the data sets indicate loss of the WAIS’s Amundsen Sea drainage sector during past warm periods, and, if confirmed, its ramifications for WAIS changes in response to ongoing and future global warming. In the following, we present and interpret our results before discussing their implications for the WAIS retreat during late Pleistocene interglacial periods.

## Results

### Clay mineral provenance of Antarctic marine sediments: fingerprinting source areas

Any interpretations of past changes in marine sediment provenance require information on the source areas of the detrital fraction. For the almost entirely ice-covered Antarctic continent, this can be achieved by investigating sediments deposited offshore on its continental margin. Physical weathering has dominated in Antarctica since the onset of widespread East Antarctic glaciation during the earliest Oligocene (~34 Ma)^24^. Consequently, clay minerals eroded below the Antarctic ice sheets and subsequently transported offshore are mainly derived from physical erosion of crystalline bedrock and ancient sedimentary strata. In contrast, clay mineral alteration by chemical processes or diagenesis is rarely observed and only of local importance^25,26^. This predominance of physical weathering of the subglacial substrate is clearly reflected by clay mineral assemblages of seafloor-surface sediments from around Antarctica, which usually contain >60% illite and chlorite, i.e., clay minerals typically formed by physical weathering^24–30^. Therefore, clay mineral assemblages in Oligocene–Holocene Antarctic sediments can be used as a tracer for the erosion, transport, and deposition of fine-grained detritus^24,25^. In the Atlantic and eastern Pacific sectors of the Southern Ocean, mapping of clay mineral assemblages in seafloor-surface sediments proximal to the ice-sheet margin allowed for the identification of source areas along the coast and further inland below the ice^26–31^. Moreover, differences between these clay mineral assemblages and the clay mineral distribution patterns in adjacent offshore regions enabled the reconstruction of oceanic transport pathways of fine-grained glacigenic material^26,27^.

We combine recently acquired and previously published clay mineral provenance data of contemporary, i.e., latest MIS-1 (14–0 ka) to modern, marine sediments from the eastern Pacific sector, including data from sites located directly at today’s WAIS front and in the sub-Antarctic SE Pacific basin (Figs. 1 and S1; Supplementary Information). The geological substrate underlying the Amundsen Sea sector of the WAIS^18,29–31^, together with late Cretaceous and middle to late Eocene sedimentary strata on the eastern Amundsen Sea continental shelf^32^, had been previously identified as a source for the clay mineral kaolinite. This source is unique in the Pacific sector because kaolinite can only be derived from pre-Oligocene strata as its formation requires weathering in low pH soil environments, i.e., in tropical climates or swamps^24–26,32^. Local submarine/subglacial outcrops of the kaolinite-rich source strata probably extend from the Amundsen Sea mid-shelf southward as far inland as the Byrd Subglacial Basin^29–33^. Our data compilation confirms kaolinite supply from the entire Amundsen Sea Embayment coast^29–31^, but, crucially, our data also reveal that subglacial erosion under Thwaites Glacier is the most important source for kaolinite delivery offshore (Figs. 1a and S2 and Table 1)^18,29–31^. In comparison, Pine Island Glacier also supplies kaolinite-bearing detritus, but this detritus is significantly more enriched in illite (Fig. S1a and Table 1)^29–31,34^.

**Table 1:** Clay mineralogical fingerprints of sources for fine-grained detritus in the SE Pacific sector of the Southern Ocean

Sector	Sub-sector	Smectite (%)	Illite (%)	Chlorite (%)	Kaolinite (%)	Esquevin index	Smectite composition
Western Antarctic Peninsula coast	South Shetland Islands area	54	17	27	2		
	West coast of Graham Land	18	29	52	0		
	**Average**	**34**	**24**	**41**	**1**		
Bellingshausen Sea coast	North & South coast of Alexander Island	8	59	33	0		
	Eastern embayment	34	38	25	3		
	Western embayment	12	58	27	3		
	**Average**	**19**	**51**	**28**	**2**		
Amundsen Sea coast	Thurston Island block	19	48	25	8	0.3–0.4	Mg < 35%
	Pine Island Glacier	8	58	17	18	≥0.4	Mg < 35%
	Thwaites Glacier	17	38	10	35	≥0.4	Mg < 35%
	Marie Byrd Land dome	21	43	18	19	≤0.3	
	**Average**	**15**	**47**	**16**	**22**		
Sub-Antarctic South East Pacific Basin	North of Bellingshausen Sea sector	39	37	21	3		
	North of the Amundsen Sea sector	40	35	16	9		Mg < 60%
	**Average**	**39**	**37**	**19**	**5**		
Interior West Antarctica	Byrd Subglacial Basin	23	22	17	39		

Averages of the smectite, illite, chlorite and kaolinite contents in the clay fraction ( < 2 µm) are given for the different source sectors (in bold font) as well as their sub-sectors and the basal glacigenic debris in the Byrd Ice Core (in normal font). The samples used to characterise the sectors and sub-sectors are given in Supplementary Data S2. The Esquevin index of illite minerals^80^ and the chemical composition of smectite minerals^36^ were only analysed on some of the samples.

**Fig. 2:**
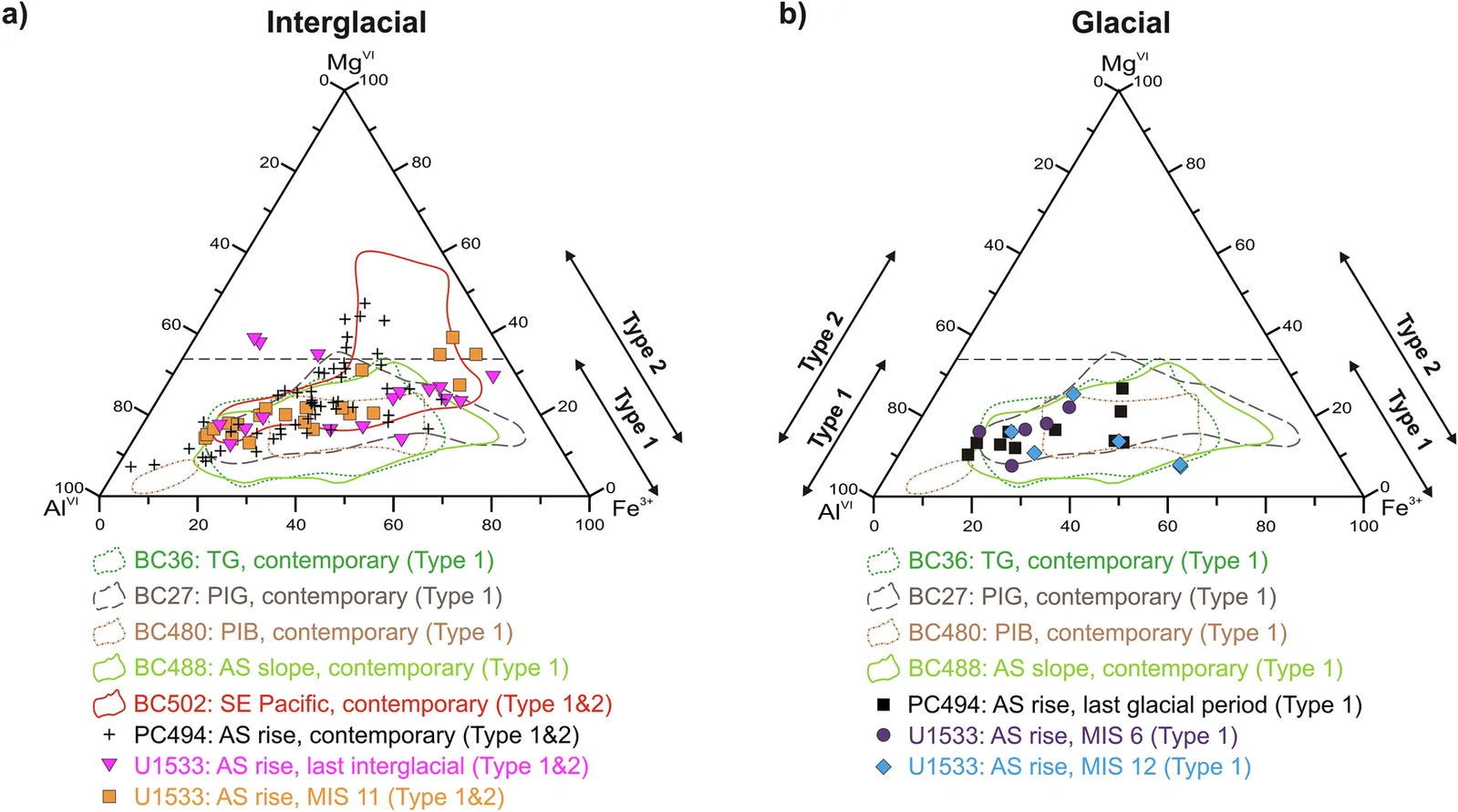
Smectite composition of interglacial and glacial sediments from the eastern Pacific sector of the Southern Ocean. **a** Average chemical (aluminium–iron–magnesium [Al–Fe–Mg]) composition of the octahedral sheet of smectite minerals in interglacial sediments from the Amundsen Sea (AS). Data for Marine Isotope Stage (MIS) 1 and MIS-5e are from Park et al.^36^. Coloured contour lines encircle compositions of individual smectite minerals analysed on contemporary samples representing potential provenance end members, including seafloor-surface sediments from the continental shelf (BC36: Thwaites Glacier [TG]; BC27: Pine Island Glacier [PIG]; BC480: Pine Island Bay [PIB]), uppermost continental slope (BC488) and the sub-Antarctic SE Pacific Basin (BC502). Samples from the shelf and slope contain Mg-poor smectites (Mg = 0–35%) only, which we refer to as Type-1 smectites, while the sub-Antarctic sample contains both Mg-poor and Mg-enriched smectites (Mg = 15–60%), which we refer to as Type-2 smectites. The horizontal dashed line separates smectites with Mg >35% from smectites with Mg <35%. Mg contents of Type-1 and Type-2 smectites are illustrated to the right of the ternary diagram. Symbols mark compositions of individual smectite minerals analysed on contemporary (PC494) and past interglacial sediment samples from the continental rise (U1533: last interglacial period, i.e., MIS-5e, and MIS-11), revealing that these samples contain a mixture of Mg-rich and Mg-poor smectites (for site locations, see Fig. 1; for smectite compositional data, see Supplementary Data S4). **b** Same as **a**, but with symbols marking compositions of individual smectite minerals analysed on glacial sediment samples from the continental rise (PC494: last glacial period, spanning MIS-2 to MIS-4^75^; U1533: MIS-6 and MIS-12) and without the field of sub-Antarctic smectites (BC502) being displayed. All glacial samples from the continental rise contain Mg-poor smectites only, indicating that these glacial sediments were predominantly sourced from the Amundsen Sea sector.

Furthermore, our compilation shows that smectite contents in contemporary Amundsen Sea continental rise sediments are too high to be explained by delivery from the WAIS margin alone (Figs. 1b and S2; see Andrews and LeMasurier^28^ and Supplementary Information for clay mineral composition of Ross Sea shelf sediments). Smectite-rich deep-sea sediments in the sub-Antarctic SE Pacific basin^35^ represent an alternative source (Figs. 1b, S2 and Table 1). Recently, the analysis of the chemical composition of smectite minerals in seafloor-surface sediments from the study area^36^ has shown that smectites on the Amundsen Sea shelf, which we refer to as Type-1 smectites, predominantly comprise a mixture of aluminium (Al)-rich smectites (montmorillonite–beidellite) and some iron (Fe)-enriched varieties (nontronite). The magnesium (Mg) contents of these Type-1 smectites vary from 5‒35%, whilst Mg-rich varieties (saponite) are absent (Fig. 2a). In contrast, smectites from the sub-Antarctic SE Pacific basin, which we refer to as Type-2 smectites, additionally include varieties with Mg contents of 35‒57% (Fig. 2a). The Mg-rich varieties of the Type-2 smectites are probably derived from submarine alteration of oceanic crust basalts^35,36^. Contemporary Amundsen Sea continental rise sediments contain a mixture of Type-1 and Type-2 smectites. This is consistent with (1) the transport of smectite-enriched suspended clay particles containing Type-2 minerals from the sub-Antarctic Pacific to the West Antarctic margin within southward flowing CDW and bottom waters, and (2) increased continent-ward dilution with Type-1 smectite-bearing clay supplied from the Amundsen Sea shelf^36^.

### Major glacial-interglacial changes in WAIS extent are archived in clay mineral provenance data from the Amundsen Sea continental rise

**Fig. 3:**
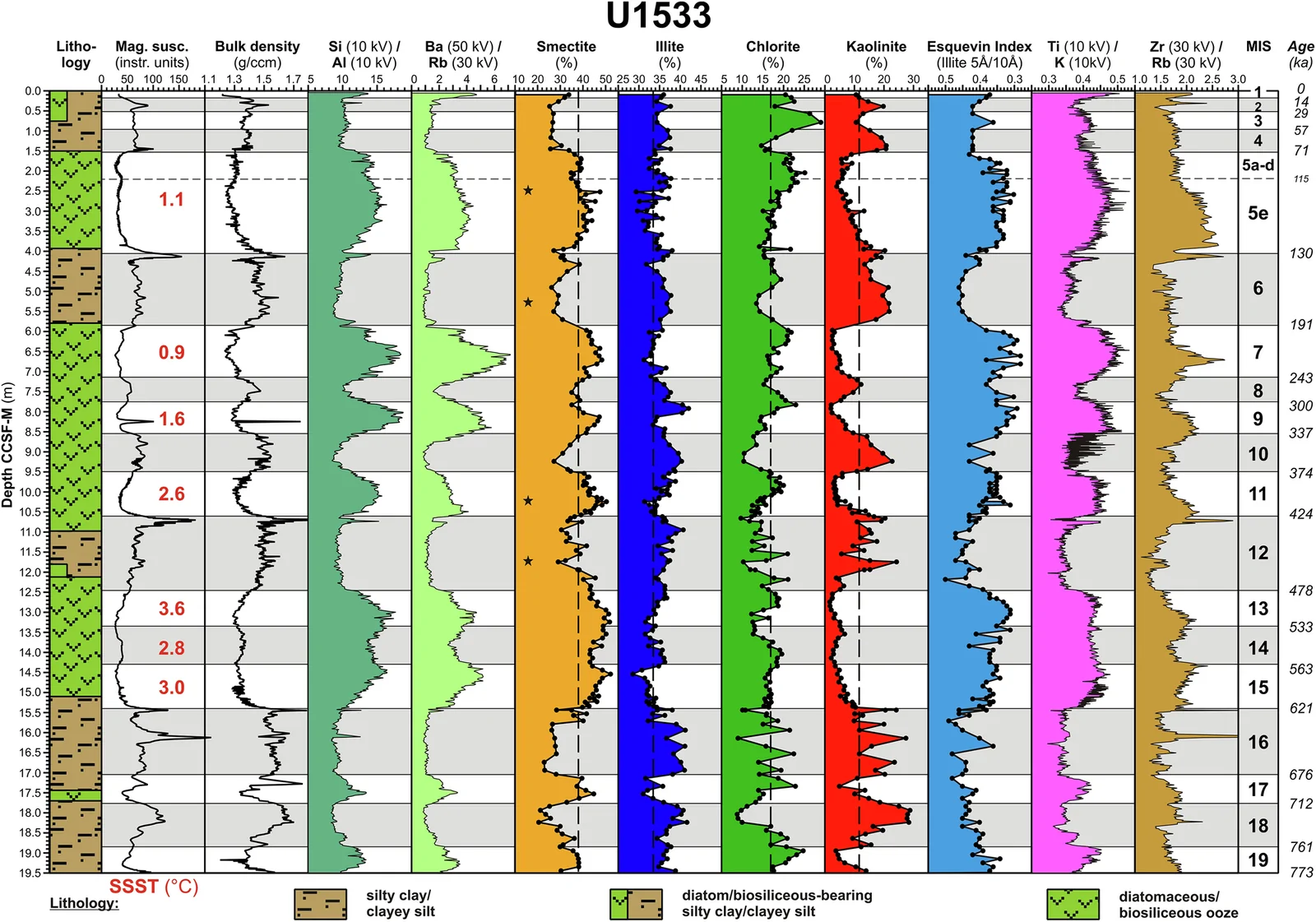
Late Pleistocene to Holocene (773–0 ka) environmental proxy record at Site U1533 on the eastern Amundsen Sea continental rise. Down-core changes in lithology, physical properties (magnetic susceptibility, gamma-ray attenuation bulk density), palaeo-productivity proxies measured by X-ray fluorescence (XRF) scanning (silica/aluminium [Si/Al], barium/rubidium [Ba/Rb]), clay mineral assemblages (smectite, illite, chlorite, kaolinite; Supplementary Data S3), Esquevin index (the illite 5 Å/10 Å peak area ratio reflecting illite composition^26,80^; note reverse *x*-axis), and proxies for sediment provenance from oceanic/continental crust (titanium/potassium [Ti/K]) and grain size (zirconium/rubidium [Zr/Rb]) measured by XRF-scanning in the upper 19.5 m core composite depth below seafloor method M (CCSF-M) at Site U1533 (lithology, physical properties and composite depth scale from Gohl et al.^23^). Data from discrete samples are marked by black dots. Vertical dashed lines in clay mineral panels mark data from the nearest contemporary surface sediment sample recovered at multiple corer site PS58/254-2^29^ located ~68 km to the SSE of Site U1533 (Fig. 1). Assignment of interglacial (white) and glacial (grey shaded) Marine Isotope Stages (MIS) is also shown (Supplementary Data S1). Black asterisks in smectite content panel mark depths of samples from MIS-5e, MIS-6, MIS-11 and MIS-12 analysed for smectite composition (Fig. 2). Red numbers in magnetic susceptibility panel give average summer sea-surface temperatures (SSSTs, in °C) reconstructed from morphological variations of frustules of the diatom species *Fragilariopsis kerguelensis* for pre-Holocene interglacial periods back to MIS-15, including glacial MIS-14^45,84^ (Supplementary Data S5). Modern SSSTs in the vicinity of Site U1533 range from −0.51 to +0.34 °C^45,85^.

**Fig. 4:**
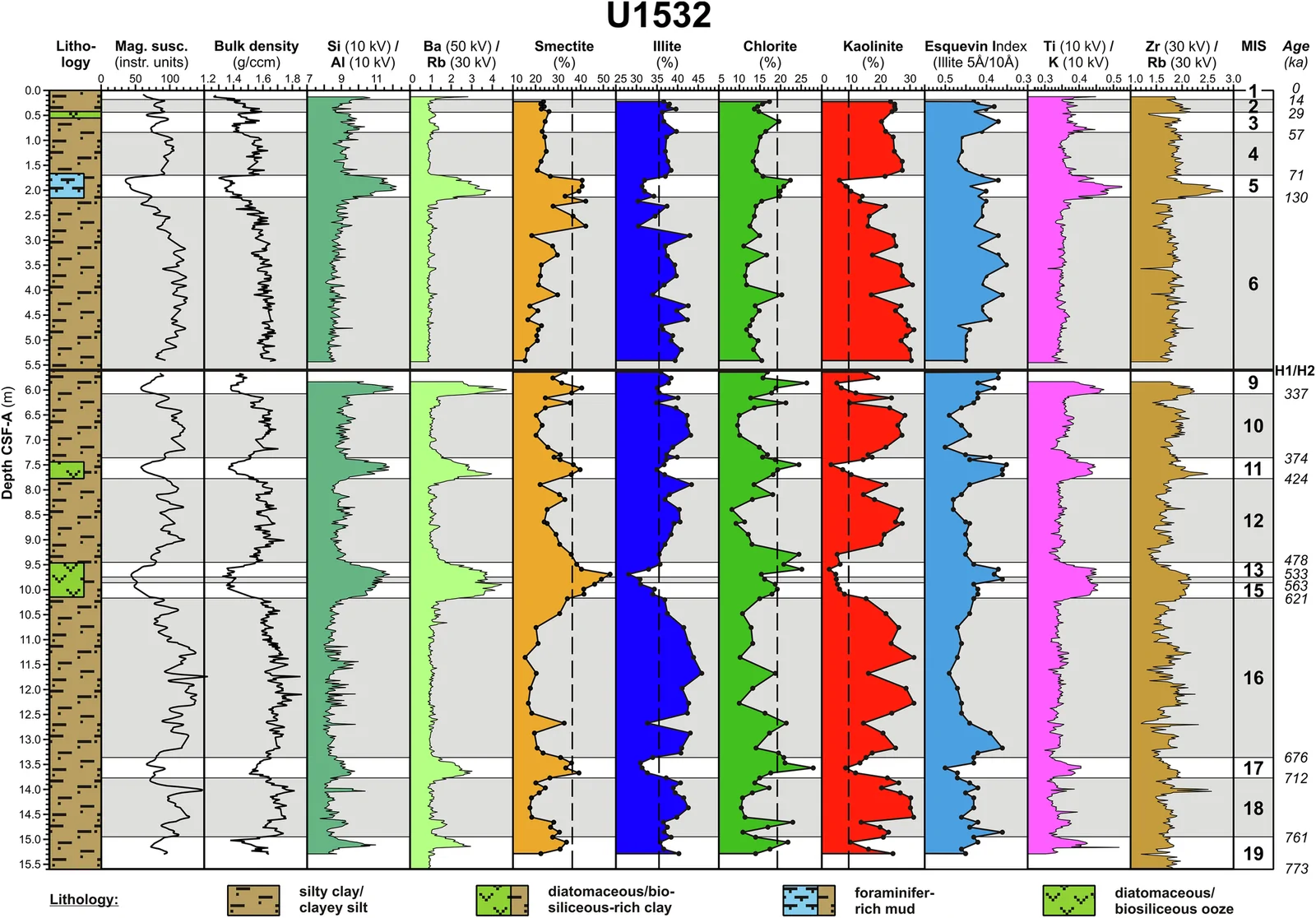
Late Pleistocene to Holocene (773–0 ka) environmental proxy record at Site U1532 on the eastern Amundsen Sea continental rise. Down-core changes in lithology, physical properties (magnetic susceptibility, gamma-ray density), palaeo-productivity proxies measured by X-ray fluorescence (XRF) scanning (silica/aluminium [Si/Al], barium/rubidium [Ba/Rb]), clay mineral assemblages (smectite, illite, chlorite, kaolinite; Supplementary Data S3), Esquevin index (the illite 5 Å/10 Å peak area ratio reflecting illite composition^26,80^; note reverse *x*-axis), and proxies for sediment provenance from oceanic/continental crust (titanium/zirconium [Ti/K]) and grain size (zirconium/rubidium [Zr/Rb]) measured by XRF-scanning in the upper 15.6 m core depth below seafloor method A (CSF-A) at Site U1532 on the Amundsen Sea continental rise (lithology, physical properties and depth scale from Gohl et al.^23^). Data from discrete samples are marked by black dots. Vertical dashed lines in clay mineral panels mark data from the nearest contemporary surface sediment sample recovered at multiple corer site PS58/253-2^29^ located ~77 km south of Site U1532 (Supplementary Data S2). XRF scanning data collected from a heavily disturbed section directly at the base of core U1532A-1H and the top of core U1532A-2H (5.46–5.80 m CSF-A)^23^ have been removed. Assignment of interglacial (white) and glacial (grey shaded) Marine Isotope Stages (MIS) is also shown (Supplementary Data S1). Comparison of physical properties, XRF-scanner data and clay mineral assemblages for sedimentary sequences retrieved at sites U1532 and U1533 during the Brunhes magnetic chron revealed a gap between cores U1532A-1H and U1532A-2H (marked by ‘H1/H2’), indicating that sediments spanning MIS-8 to MIS-7 (300–243 ka) were either not recovered or are absent at Site U1532 (Supplementary Data S1 and Fig. S3).

Sediment cores at IODP Exp. 379 Site U1533 were recovered from 4180 m water depth on the distal part of the gentle western flank of “Resolution Drift”, a sediment drift on the eastern Amundsen Sea continental rise^23^. Site U1532 was drilled at 3960 m water depth on the same flank near the drift crest (Fig. 1). The late Pleistocene‒Holocene strata at both sites predominantly consist of bioturbated diatom-bearing and (rarely) foraminifera-bearing sediments that alternate with laminated, terrigenous muds^23^ (Figs. 3 and 4)—similar to other cores from the Amundsen Sea margin^18,37^. Elevated barium (Ba)/rubidium (Rb) ratios, a proxy for biogenic barium enrichment^18,37–39^ derived from X-ray fluorescence (XRF) core scanning, correlate with increased diatom abundance, as inferred from microscopic investigation, high silica (Si)/Al ratios, and low sediment density^23^. The same relationship between diatom abundance, biogenic Ba concentration, density and biogenic opal content was reported for sediments spanning the Brunhes chron, which were recovered in piston core PS58/254-1^37,38^, located just ~68 km SSE of Site U1533 on Resolution Drift (Fig. 1). As previously inferred from the Ba/Rb record of Site U1533 by Hopkins et al.^39^, the down-core Ba/Rb maxima at sites U1532 and U1533 delineate warmer periods of increased phytoplankton productivity in surface waters of the Amundsen Sea^18,37,38^ (Fig. S3). Thus, these maxima document that the coinciding diatom abundance maxima are not the result of enhanced siliceous microfossil preservation^18,37–39^. The interbedded terrigenous silty clays, characterised by high magnetic susceptibility and density, were deposited during glacial periods, probably by interaction of a bottom current with the fine-grained suspension load of turbidity currents travelling through a deep-sea channel flanking Resolution Drift^23,38,39^. These turbidity currents were likely triggered by gravitational instabilities on the Amundsen Sea continental slope, caused by the enhanced supply of unsorted glacigenic debris to the shelf break by grounded ice-stream advances across the shelf during glacials^18,23,37–39^. Interglacial sediment intervals at Site U1533 are expanded and glacial intervals are condensed when compared to Site U1532 (Figs. 3, 4 and S3). This disparity most likely results from the specific locations of the two sites on Resolution Drift. The relative contributions of different sediment transport processes affecting drift deposition throughout late Pleistocene glacial-interglacial cycles are known to vary systematically across Antarctic rise drifts, creating distinct, localised depositional settings^40^.

In both IODP Exp. 379 records the zirconium (Zr)/Rb ratio, a proxy for mean grain size in silty-clayey Antarctic margin sediments^41,42^, reveals a slight coarsening and thus higher interglacial bottom-current speeds when compared to glacials (Figs. 3 and 4). Magnetic susceptibility peaks occur at several glacial terminations (cf. ref. ^37^) and may, like coinciding Zr/Rb maxima, indicate enhanced delivery of iceberg-rafted debris (IRD). The deposition of IRD on the Amundsen Sea continental rise probably increased when fringing parts of the WAIS grounded on the adjacent shelf disintegrated due to warming and/or sea-level rise at the onset of glacial retreat. The resulting calving of numerous icebergs led to increased IRD deposition offshore, as observed in other sediment records from the Antarctic margin and Southern Ocean^27,43^. However, the exact relationships between magnetic susceptibility, Zr/Rb ratios and IRD content at the IODP Exp. 379 sites still need to be investigated in detail.

At sites U1533 and U1532, kaolinite supplied from the Amundsen Sea sector is significantly enriched in glacial sediments, whereas smectite is enriched in the interglacial deposits (Figs. 3 and 4). These changes are consistent with the proposed depositional model and down-core clay mineral assemblage data of cores PS2551-1 and PS58/254-1 recovered from the western and eastern Amundsen Sea continental rise, respectively^18,37,38^ (Fig. 1). The high kaolinite concentrations in the glacial sediments were attributed to the increased supply of glacigenic detritus from the Amundsen Sea sector during cold periods. At these times, the grounded WAIS had advanced to the shelf break and delivered kaolinite-enriched detritus eroded in interior West Antarctica and on the Amundsen Sea shelf directly to the continental slope and rise. Illite and chlorite contents at sites U1533 and U1532 show only a subdued glacial-interglacial pattern. Illite concentrations are slightly elevated in glacial sediments, whilst chlorite concentrations are slightly increased in sediments deposited during late stages of interglacials (cf. refs. ^18,37^). The Esquevin index is a proxy for the chemical composition of illite, with a value > 0.40 indicating an Al-rich (i.e., muscovitic) composition and a value < 0.15 indicating a Fe- and Mg-rich (i.e., biotitic) composition^26^. At Site U1533, its value is almost consistently >0.40 in the glacial sediments (Fig. 3). This equally points to increased input of detritus eroded by ice streams draining into the eastern Amundsen Sea during glacials, with Pine Island Glacier delivering illite-enriched detritus with Esquevin index values of 0.4–0.6^29,31,34^ (Fig. S1a and Table 1).

### Late Pleistocene variability in ocean forcing reconstructed from smectite composition and diatom data

The smectite maxima in past interglacial sediments at both IODP sites cannot be explained by enhanced detrital supply from neighbouring Antarctic sectors, such as the Bellingshausen Sea and Ross Sea coasts, because smectite contents in glacigenic sediments from these source areas are too low (Table 1, Supplementary Information). Therefore, the interglacial smectite increase must, as at present, at least partly result from the delivery of smectite-enriched detritus from the sub-Antarctic SE Pacific basin^36^ (Figs. 1b and S2). This is evident from the smectite composition in interglacial (MIS-5e; MIS-11: 424–374 ka) and glacial (MIS-6: 191–130 ka; MIS-12: 478–424 ka) samples from Site U1533 and in a glacial sample (MIS-4 to MIS-2: 71–14 ka) from piston core PC494 (Fig. 2), which was recovered near the crest of Resolution Drift ~149 km south of Site U1533 (Fig. 1). All investigated interglacial samples contain a mixture of Type-1 smectites characterising Amundsen Sea shelf sediments and Type-2 smectites typifying sub-Antarctic SE Pacific basin sediments (Fig. 2a). In contrast, the glacial samples seem to contain exclusively Type-1 smectites, which is evident from the absence of smectites with Mg-contents >35% (Fig. 2b). The titanium/potassium (Ti/K) ratios are increased in the interglacial sediments at both IODP sites (Figs. 3 and 4). Titanium is usually enriched in basaltic rocks and, thus, oceanic crust, while potassium is typically enriched in continental crustal rocks, such as granites. Therefore, the Ti/K ratio in marine sediments often reflects the relative proportions of siliciclastic detritus from oceanic versus continental sources^44^. Like the presence of smectites with Mg contents >35%, high Ti/K ratios in the interglacial sediments from the Amundsen Sea continental rise indicate enhanced detrital supply from the sub-Antarctic Pacific.

If the smectite increase during interglacial periods solely resulted from the reduced input of shelf-derived glacigenic detritus enriched in kaolinite (mainly—but not only—sourced from Thwaites Glacier; Table 1) and containing some more illite (mainly sourced from Pine Island Glacier), then the chlorite content in the interglacial sediments from the Amundsen Sea continental rise should also have increased. However, no clear glacial-interglacial variability is recorded by the chlorite contents (Figs. 3 and 4). Conversely, the high kaolinite contents of the glacial sediments cannot have been caused exclusively by the reduced smectite delivery from the sub-Antarctic SE Pacific basin. In this case, an increase of both illite and chlorite, matching the prominent kaolinite increase, would be expected. However, the illite concentrations show only a very subtle increase, while no systematic change in chlorite content is recorded (Figs. 3 and 4; cf. ref. ^37^). These findings suggest that the observed high kaolinite concentrations in the glacial sediments originate from both increased delivery of detritus from the Amundsen Sea sector due to WAIS advance across the shelf and decreased supply from the sub-Antarctic SE Pacific basin due to reduced poleward CDW advection (Figs. 3, 4, and S2). Importantly, we observe the same glacial-interglacial pattern in smectite and kaolinite contents at both IODP Exp. 379 sites. The systematic pattern persists despite sedimentation rates at Site U1532 having been higher during glacials than during interglacials at this site and during most glacials at Site U1533. In contrast, sedimentation rates at Site U1533 lack, at least during the late Pleistocene, a systematic glacial-interglacial pattern, but were during interglacials of the last 800 kyr consistently higher than at Site U1532 (Fig. S3 and Supplementary Data S1).

The limited number of compositional analyses on smectite minerals from glacial sediments (Fig. 2b) is largely due to the low smectite content in these samples. This is probably a consequence of the high glacial delivery of Amundsen Sea sector-derived detritus, including Type-1 smectites, which overwhelmed the supply of detritus from the sub-Antarctic Pacific. Thus, the apparent absence of Type-2 smectites in the glacial samples may simply reflect high dilution with Type-1 smectites. A recently published reconstruction of summer sea-surface temperature (SSST) for MIS-5e sediments at Site U1533 revealed SSSTs of up to 1.3–2.2 °C warmer than present^45^. The reconstruction had utilised an established proxy based on temperature-dependent morphological variations of frustules of the diatom species *Fragilariopsis kerguelensis*^45^. Application of the same method to interglacial sediments at Site U1533 since MIS-15 (621–563 ka) provides pre-Holocene SSSTs, binned for each interglacial MIS, between 0.6–1.4 °C (MIS-7: 243–191 ka) and 3.3–4.1 °C (MIS-13: 533–478 ka) warmer than today (Fig. 3 and Supplementary Data S5). Since intensified poleward advection of CDW would also lead to surface-ocean warming^46^, these SSST reconstructions support the hypothesis that the smectite increase, together with the presence of Type-2 smectites in the interglacial sediments, resulted from increased CDW advection towards West Antarctica.

## Discussion

### Interglacial clay mineral assemblages: ocean-forced ungrounding of Thwaites Glacier and the Amundsen Sea drainage sector

Notably, Site U1533 (Fig. 3) records considerably reduced kaolinite contents (<5%) during each interglacial after 800 ka, including MIS-14 (563–533 ka), which lacks characteristics of a glacial period on the Antarctic continental margin and globally^37^. Kaolinite concentrations at Site U1532 show a similar pattern (Fig. 4), although interglacial reductions are less pronounced. This is probably caused by the condensed interglacial deposition at Site U1532 (~1 cm/kyr; Fig. S3 and Supplementary Data S1), which results in a relatively coarse temporal resolution of interglacial samples. Kaolinite minima <5% during pre-Holocene interglacials at Site U1533 compare to 12% kaolinite in the modern sample from nearby multiple core PS58/254-2, with kaolinite content being consistently ≥8% in contemporary sediments within at least ~400 kilometres around both IODP sites^18,27,29^ (Fig. 1a). In some interglacial intervals, kaolinite is nearly absent (<2%) at Site U1533 (Fig. 3). This major provenance change demonstrates that during pre-Holocene interglacials only very limited fine-grained glacigenic detritus was delivered to the adjacent continental rise from the Amundsen Sea drainage sector, and specifically from the kaolinite-rich beds beneath Thwaites Glacier. We thus interpret the prominent provenance shift to indicate a cessation of subglacial erosion under this part of the WAIS, requiring widespread ungrounding and deglaciation of the Thwaites Glacier catchment (Fig. 1a). Moreover, because Pine Island Glacier also supplies kaolinite-bearing detritus (kaolinite content 14–19%)^29,31,34^ (Table 1 and Supplementary Data S2), its near-absence in interglacial intervals at Site U1533 implies substantial retreat of this ice stream, too.

Previous ice-sheet model simulations had reconstructed a wide range of WAIS configurations for the last interglacial period^47^ by deducing major retreat of marine-based grounded ice in (1) the Weddell and Bellingshausen Sea drainage sectors^48,49^, (2) the Amundsen and Bellingshausen Sea sectors as well as parts of the Ross and Weddell Sea sectors^12,13,49^, or (3) across the entire WAIS^5,11,50^. Other model simulations reconstructed WAIS extent nearly identical to today^51,52^. However, even if all marine-based ice in the Amundsen Sea catchment was lost, isostatically adjusted subglacial bed topography^53^ shows that a land-based ice cap would likely have survived on the Marie Byrd Land dome (Fig. 1). Smaller ice caps probably also persisted on an island archipelago in today’s Thurston Island block area, which includes Thurston Island and smaller peninsulas/islands located further south along the eastern Amundsen Sea Embayment coast^5,11–13^.

Subglacial erosion in the Marie Byrd Land dome region, where Cretaceous sedimentary strata of similar age as those cropping out on the eastern Amundsen Sea shelf^32^ are eroded under the ice today^54^, and offshore delivery of the produced detritus likely continued even when marine-based ice was absent or afloat. This is evident from elevated (>10%), yet lower than contemporary (17%), kaolinite contents maintained throughout interglacial periods since 800 ka at the western Amundsen Sea rise site PS2551-1 offshore from the Marie Byrd Land dome^18^. At Site U1533 the Esquevin index value of illite in the pre-Holocene interglacial sediments mainly ranges from 0.30–0.40 (Fig. 3). This range reveals that the illite—together with other detritus—was predominantly derived from the Thurston Island block, where Esquevin index values typically also vary from 0.30–0.40^29^ (Table 1), and, thus, corroborates continued glacial erosion in the Thurston Island block area. In contrast, illites sourced from Marie Byrd Land typically have Esquevin index values ≤ 0.30^29^ (Table 1). The recorded interglacial clay mineral provenance is also consistent with transport pathways of detrital particles in some modelled WAIS collapse scenarios, where the marine-based sectors of the WAIS are deglaciated and prevailing surface winds blowing across the Amundsen Sea surface are NNW- to NW-directed^55^. Such a wind pattern would result in surface currents and icebergs transporting glacigenic detritus eroded on the Thurston Island block towards both IODP sites. Detritus derived from the eastern Marie Byrd Land dome, however, would be carried towards site PS2551-1, located ~340 kilometres west of Site U1533 (Fig. 1).

The presence of Mg-rich smectites in interglacial sediments on the Amundsen Sea continental rise indicates that the interglacial smectite increase is partly caused by a high supply of Type-2 smectites from the sub-Antarctic SE Pacific basin (Fig. 2a). The increased delivery of Type-2 smectites is likely to reflect an interglacial intensification of southward CDW advection to the West Antarctic continental margin, which is also evident from the elevated SSSTs (Fig. 3). We conclude that this process led to major WAIS retreat during all previous interglacial periods of the last 800 kyr, consistent with modelled partial or total WAIS collapse during MIS-5e in response to thermal ocean erosion at the floating margins of the ice sheet^11–13^. Similarly, WAIS retreat at the end of the last glacial period and since the mid-twentieth century has been attributed to ocean forcing based on numerical simulations^11,56^ and data^34,42,57–59^, respectively. The dominant role of the ocean driver for WAIS changes is also suggested by reconstructions of deep-water temperatures in the pelagic Southern Ocean and the sub-Antarctic region that concluded CDW warming throughout late Pleistocene interglacial periods and the Holocene^60,61^.

For MIS-5e, our data indicate maximum WAIS retreat at a relatively late stage (Fig. 3), in support of published genetic evidence pointing to the opening of a Trans-Antarctic seaway as the result of a WAIS collapse late during the last interglacial^20^ (Supplementary Information). Reconstructions of global mean sea level during MIS-5e concluded that it peaked already around 127–125 ka^8^, but several regional studies recorded two highstands centred at ~129 ka and ~117 ka^9^^,^^10^. Differences in the timing of relative sea-level maxima may result from (1) chronological uncertainties of individual sea-level archives, for example, due to coral carbonate diagenesis^8,62^, (2) influences of glacial isostatic adjustment on relative sea-level recordings^8^^,^^16^, and (3) asynchronous or multiphase melting of polar ice sheets (WAIS, East Antarctic Ice Sheet, Greenland Ice Sheet) throughout MIS-5e^8,13–16,62^. Regardless, our findings agree with model experiments simulating a sea-level rise of ~3 m from Antarctic ice-sheet deglaciation around 116 ka, mainly triggered by the ocean-forced collapse of the Amundsen Sea sector^12^. Furthermore, our results are consistent with the scenario of an additional sea-level highstand at the end of MIS-5e^10^.

### Global implications

Our data document the ungrounding of Thwaites Glacier and widespread deglaciation in the Amundsen Sea sector of the WAIS during each of the pre-Holocene warm periods since 800 ka. This ice loss would have contributed at least ~1.5 m to global sea level^3^. Previously published model experiments have suggested that deglaciation of the Amundsen Sea drainage sector^7,11^ and especially the Thwaites Glacier catchment^6^ could be the first phase or initiation of major WAIS retreat. Our results do not reveal the full extent of WAIS retreat during the late Pleistocene interglacial periods, i.e., whether the ice sheet underwent full or only partial collapse. Even though they can neither provide indisputable evidence for nor dismiss model projections concluding, for example, a (West) Antarctic contribution of ~3–4 m to the sea-level highstand of MIS-5e^5,8,12,13^, they do corroborate a considerable WAIS contribution of at least 1.5 m.

Taking into account recently published findings on the geochronological and geochemical provenance of the Early to Mid-Pliocene sediments at sites U1532 and U1533, partial WAIS collapses during late Pleistocene interglacials seem likely. For several Pliocene interglacial periods, the provenance data point to a retreat of grounded ice in the Amundsen Sea sector all the way back to the Ellsworth-Whitmore Mountains in the WAIS’s Weddell Sea drainage sector^19,63,64^ (Fig. 1). These major WAIS retreats were inferred from changes in the neodymium isotope composition of the bulk sediments^19^ and the lead isotope composition of the fine-grained detritus^63^ as well as the argon-40/argon-39 (^40^Ar/^39^Ar) ages of iceberg-rafted biotite and hornblende grains^64^. The neodymium isotope investigation also targeted the Pleistocene interval and concluded rather limited interglacial WAIS retreat during this time^19^. Our results confirm that the Amundsen Sea drainage sector collapsed during MIS-5e. They may not rule out, however, that the Weddell and Ross Sea sectors survived, which would agree with some model simulations^13^ and an ice-core derived reconstruction for the Weddell Sea sector^65^ but possibly not with recently published ice core evidence from the Ross Sea sector^66^ (Supplementary Information). Scenarios of WAIS survival in the Weddell and/or Ross Sea regions could only be reconciled with the genetic evidence^20^, if floating ice shelves rather than grounded ice remained present in these sectors, which is supported by some model results^65,67^. Furthermore, such scenarios would likely require a substantial sea-level contribution from ice loss in East Antarctica^15^.

In any case, our results reveal that natural climatic conditions were evidently sufficient to cause major WAIS deglaciation during previous late Pleistocene warm times. They emphasise that global climate warming of as little as 1.0–1.5 °C above pre-industrial temperatures—which occurred during MIS-5e^8^ and matches regional SSST reconstructions for the Amundsen Sea^45^ (Fig. 3 and Supplementary Data S5)—can constitute a tipping point for major WAIS deglaciation^61,68^. Model simulations highlight that anthropogenic greenhouse gas emissions since the 1960s, combined with atmospheric ozone depletion since the 1980s, have warmed Southern Ocean waters and caused wind anomalies over the shelf break in the Amundsen Sea, which increased CDW upwelling^56^. These processes have maintained ocean-forced WAIS melting in the Amundsen Sea sector over the last few decades^56^, which will, even if held constant, most likely result in the deglaciation of the Thwaites and Pine Island Glacier drainage basins over the coming centuries^69^. Therefore, our results raise the prospect that the WAIS may be committed to further major retreat even without continued anthropogenic greenhouse gas emissions and that human-induced global temperature increase will likely accelerate and amplify natural interglacial WAIS loss and associated global sea-level rise in the future.

## Methods

### IODP Expedition 379 drill cores and age models

During IODP Expedition 379 in austral summer 2019, RV *JOIDES Resolution* drilled sites U1533 and U1532 on the western flank of Resolution Drift^23^ (for core site locations, see Fig. 1). Resolution Drift is a sediment drift located on the Amundsen Sea continental rise directly offshore from the eastern Amundsen Sea Embayment, which is a main gateway for ice draining the WAIS via the major ice streams Thwaites Glacier and Pine Island Glacier^23^. Site U1532 (68° 36.7′ S, 107° 31.5′ W, water depth 3960 m) is located on the upper flank of the drift and retrieved latest Miocene to Holocene sediments in five holes (A–D, G) from a maximum drill depth of 787 m with an average recovery of 90%^23^. Site U1533 (68° 44.1′ S, 109° 01.5′ W, water depth 4180 m) is situated 62 km further WSW on the lower drift flank and recovered latest Miocene to Holocene sediments in four holes (A–D) from a maximum drill depth of 383 m with an average recovery of 76%^23^. The western flank of Resolution Drift is bound by an N–S-oriented deep-sea channel west of Site U1533^23,70^. The channel marks the pathway of glacigenic detritus transported downslope from the shelf by turbidity currents and gravity flows and, as such, is likely the major source of terrigenous sediment deposited at the drill sites^23,37–39,64,70^. Bottom currents, assumed to be flowing eastwards, transported clay- and silt-sized particles before depositing them to form the drift^23,38,39,64,70^.

At Site U1533, late Pleistocene to Holocene sediments were retrieved in Holes U1533A, U1533C and U1533D, with the Brunhes–Matuyama chron boundary (C1n base at ~773 ka) being detected in holes U1533A and U1533D^23^. In Hole U1533A, high-resolution magnetic analyses carried out post-cruise identified this chron boundary at 19.44 ± 0.05 m core composite depth below seafloor method M (CCSF-M)^39^. At Site U1532, sediments of this age were only recovered in Hole U1532A, where the Brunhes–Matuyama chron boundary was encountered at 15.64 ± 0.88 m core depth below seafloor method A (CSF-A)^23^. Recoveries for the late Pleistocene–Holocene sediments at sites U1533 and U1532 slightly exceeded 100%. Biostratigraphic constraints for the Brunhes chron were provided by the last appearance datums (LAD) of the diatom *Actinocyclus ingens* (>600 ka) at 17.3 ± 2.3 m CCSF-M in Hole U1533A and the diatom *Rouxia constricta* (>300 ka) at 9.8 ± 0.0 m CSF-A in Hole U1532A^23^. We established high-resolution age models spanning the Brunhes chron for sites U1533 and U1532 by correlating maxima in barium (Ba)/rubidium (Rb) ratios, which reflect maxima in biogenic barium content and, thus, biological productivity^37,38,57,71–73^, with interglacial Marine Isotope Stages (MIS) in the LR04 benthic foraminifera oxygen isotope stack^74^ (Supplementary Data S1 and Fig. S3). Comparison of physical properties, X-ray fluorescence (XRF) scanner data and clay mineral assemblages for sedimentary sequences retrieved at sites U1532 and U1533 during the Brunhes magnetic chron revealed a gap between cores U1532A-1H and U1532A-2H. The gap indicates that sediments spanning MIS-7 to MIS-8 (243–300 ka) in age were either not recovered or are absent at Site U1532 (Fig. S3). Our age model for Site U1533 is nearly identical to the “barium age model” previously published by Hopkins et al.^39^ and merely represents a refinement of the latter. In detail, we constructed the age models for sites U1533 and U1532 by correlating the bases and tops of Ba/Rb maxima with the beginnings and ends of interglacial MIS in the LR04 stack (Fig. S3). Thereby, we used the identified base of the Brunhes chron at both sites as a guide rather than a fixed age-depth point (cf. ref. ^39^). The validity of these Ba/Rb-derived age models is supported by the late Pleistocene–Holocene geomagnetic relative palaeointensity (RPI) record for Site U1533^39^. Despite the RPI-derived ages being consistently slightly younger than the Ba/Rb-derived ages during the last 800 kyr, the RPI and Ba/Rb age models are largely in agreement^39^. The observed offset of the RPI-derived ages most likely results from the relatively low accumulation rate of ~2.3 cm/kyr at Site U1533. This low accumulation rate caused changes of the palaeomagnetic signal to be locked in the sediment column at an estimated seabed depth of ~26 cm rather than at the seafloor, resulting in temporal offsets of up to ~13 kyr^39^.

### Contemporary seafloor surface sediment samples and conventional cores

Clay mineral data were obtained for this study from 33 seafloor-surface sediment samples collected in the vicinity of the Thwaites Glacier and Pine Island Glacier fronts with a multiple corer (27 samples), box corer (4 samples), Kasten corer (1 sample) and gravity corer (1 sample). All these samples were retrieved on cruises NBP1902 and NBP2002 with RV/IB *Nathaniel B. Palmer* of the Thwaites Glacier Offshore Research (THOR) project, which forms part of the International Thwaites Glacier Collaboration (ITGC) program, during austral summer 2019 and 2020, respectively. In addition, we here present clay mineral data from 19 seafloor-surface sediment samples, which were collected from the Amundsen Sea continental rise and the eastern Pacific sector of the Southern Ocean with a multiple corer on expedition ANT-XVIII/5a with RV *Polarstern* in austral summer 2001, and from 11 core-top samples, which were collected in the wider region on various other cruises. The clay mineral assemblage data of these contemporary seabed sediments were combined with previously analysed clay mineral assemblage data on seafloor-surface sediments^26,27,29–31,73^ (Supplementary Information) to build a comprehensive clay mineral provenance data set from the eastern Pacific and western Atlantic sectors of the Antarctic continental margin and Southern Ocean (Supplementary Data S2). Most samples in the dataset were collected from 0–1 cm core depth (up to 0–3 cm, where available sample material was already depleted) in multiple and box cores to obtain undisturbed modern seafloor-surface sediments. Core-top samples from gravity, Kasten, piston, percussion and vibro-cores were only included where the clay mineralogical fingerprint of potential source areas on the shelf hitherto had not been sufficiently characterised by clay mineral analyses (i.e., the eastern and western Antarctic Peninsula, South Orkney, and Weddell Sea shelves), or where previously published data sets forming part of our compilation had included such samples. At the deep-water sites, nearly all surface sediment data are from multiple or box cores to avoid the risk of “fingerprinting” early MIS-1 or even pre-MIS-1 sediments. This is because at those sites, sedimentation rates for MIS-1 can be very low. Moreover, surface sediments recovered in piston and gravity cores can be heavily disturbed—for example, when a piston penetrates the seabed or the “soupy” seafloor surface sediments start to flow during core recovery—or may be entirely lost, when a gravity corer over-penetrates (e.g., refs. ^37,73^).

To investigate the smectite composition in a contemporary, undisturbed seafloor surface sediment sample from the vicinity of the IODP Exp. 379 drill cores, we analysed a sample from the box core BC495. BC495 had been recovered together with piston core PC494 ~ 149 km south of Site U1533 from the crest of Resolution Drift (70° 02.9′ S, 108° 20.9′ W, water depth 3468 m) during cruise JR179 with RRS *James Clark Ross* in austral summer 2008 (Fig. 1; for simplicity, we refer to both cores uniformly as “PC494” in text and figures). Because the sediments spanning the last glacial period from MIS-2 (29–14 ka) to MIS-4 (71–57 ka) are more condensed at sites U1532 and U1533 than at site PC494, we also targeted core PC494 for a smectite composition sample from the last glacial period. PC494 retrieved a 10.4-metre-long sequence predominantly consisting of structureless to laminated silty clays, with a sandy mud interval with foraminifera from 0–0.2 m and a foraminifera- and diatom-bearing mud interval from 4.7–5.2 m^75^. Based on the lithostratigraphic changes in core PC494 and correlation of its RPI record with the independently dated SINT-800 palaeointensity stack^76^, the sandy mud interval at the core top was assigned to MIS-1, and the lower foraminifera- and diatom-bearing interval was assigned to MIS-5 (130–71 ka)^75^. The purely terrigenous silty clay intervals from 0.2–4.7 m and below 5.2 m were assigned to the glacial periods spanning MIS-2 to MIS-4 and MIS-6, respectively^75^ (Supplementary Data S1). As in many other cores from the Antarctic continental margin^18,27,37,40,71,73^, sediments deposited at site PC494 during the time spanning MIS-2, MIS-3 (57–29 ka) and MIS-4 have the characteristics of a single, pronounced glacial period, and MIS-3 could not be reliably identified based on lithological changes or proxy evidence^75^.

### Physical properties and X-ray fluorescence data

Magnetic susceptibility and gamma-ray attenuation (GRA) bulk density on whole core sections from Sites U1533 and U1532 were measured at 2-cm depth resolution during IODP Exp. 379 using a whole-round multi-sensor core logger^23^. XRF scans on the archive halves of the Pleistocene‒Holocene core sections from sites U1533 and U1532 were conducted post-cruise at 2-cm depth resolution using an Avaatech XRF core scanner at the IODP Gulf Coast Repository (College Station, Texas, USA). The archive halves were scanned and analysed for a range of elements, with silica (Si) and aluminium (Al) being measured at 10 kV for determining the Si/Al ratio as a proxy for biogenic opal^72,73^, titanium (Ti) and potassium (K) being measured at 10 kV for determining the Ti/K ratio as a proxy for the supply of siliciclastic detritus derived from oceanic and continental crust^44^, rubidium (Rb) and zirconium (Zr) being measured at 30 kV for determining the Zr/Rb ratio as a grain-size proxy^41,42^, and barium (Ba) being measured at 50 kV for determining the Ba/Rb ratio as an indicator for the content of biogenic Ba, which is a proxy for biological productivity^18,37,38,71–73^.

### Clay mineral assemblages

Clay mineral assemblages were analysed on down-core sediment samples taken at 10- to 20-cm depth resolution from sites U1533 (200 samples from Holes U1533A and U1533D), U1532 (102 samples from Hole U1532A) and PC494 (64 samples) (Supplementary Data S3). The relative clay mineral contents in the previously published surface-sediment samples had been determined with X-ray diffraction (XRD) techniques according to procedures and methods described in detail elsewhere^24,26,29,31^. The clay mineral samples investigated for this study were processed and analysed with XRD at the Institute for Earth System Science and Remote Sensing, University of Leipzig (Leipzig, Germany). First, the clay fraction (<2 μm) was separated from the bulk sediment in settling tubes. Clay (40 mg) was dispersed in an ultrasonic bath and mixed with 1 ml of an internal standard consisting of a 0.04% MoS_2_ suspension. The samples were mounted as texturally oriented aggregates by rapidly filtering the suspension through a membrane filter of 0.20 μm pore width. The filter cakes were dried at 50 °C and mounted on aluminium tiles before being exposed to ethylene glycol vapour at a temperature of 60 °C for at least 18 h immediately before the XRD analyses. The measurements were conducted on an automated powder diffractometer system Rigaku MiniFlex with CoKα radiation (30 kV, 15 mA). The samples were X-rayed in the range 3°–40° 2*θ* with a scan speed of 0.02° 2*θ*/s. Additionally, the range 28°–30.5° 2*θ* was measured with a step size of 0.01° 2*θ*/s to better resolve the (002) kaolinite peak and the (004) chlorite peak. The X-ray diffractograms were evaluated using the “MacDiff” program (freeware available from https://www.uni-frankfurt.de/69528130/Petschick___MacOS_Software).

The clay mineral investigations in this study focus on the main clay mineral groups: smectite, illite, chlorite, and kaolinite. These clay minerals were identified by their basal reflections at ~17 Å (smectite), 10 Å and 5 Å (illite), 14.2 Å, 7 Å, 4.72 Å, and 3.54 Å (chlorite), and 7 Å and 3.57 Å (kaolinite). Semi-quantitative evaluations of the mineral assemblages were made on the integrated peak areas. The relative percentages of smectite, illite, chlorite, and kaolinite were determined using empirically estimated weighting factors^26,77–79^. No effort was made to quantify mixed-layer clay minerals.

The illite composition was determined from the 5/10 Å peak area ratios ( = Esquevin index)^80^, with an Esquevin index value > 0.4 corresponding to Al-rich illites (muscovite)^26^. The Esquevin index value decreases with magnesium (Mg) and iron (Fe) substituting for the octahedral Al. Therefore, Mg- and Fe-rich illites (biotite) have an Esquevin index value < 0.15^26^. By preparing, analysing and evaluating samples six times, a standard deviation of ~1% was achieved for the contents of smectite, chlorite and kaolinite, and of ~1.5% for illite. The standard deviation for the Esquevin index is ~0.05 Δ°2*θ*.

### Smectite composition

The average chemical (Al–Fe–Mg) composition of the octahedral sheet of smectite clay minerals was investigated on selected samples following the methodology given in Park et al.^36^. Transmission electron microscopy (TEM) analysis was performed to acquire the structural and chemical variations in the smectites using a JEOL JEM-F200 fitted with an energy dispersive X-ray spectrometer (EDS) for JEM-F200 operated at a voltage of 200 kV at Yonsei University (Seoul, Republic of Korea). The TEM samples were selected from contemporary seafloor-surface sediments from the continental shelf and rise in the Amundsen Sea and the sub-Antarctic SE Pacific Ocean, as well as glacial (MIS-2–4, MIS-6, MIS-12) and interglacial (MIS-1, MIS-5, MIS-11) sediment intervals at sites U1533 and PC494 (Fig. 2 and Supplementary Data S4). The samples were impregnated with LR White resin, followed by application of a water/methanol exchanging process to prevent layer collapse in the smectite (from 13 Å to 10 Å, with the latter spacing being identical to the illite spacings) under the high-energy electron beam during TEM operation. The impregnated samples were then sectioned at 100 nm thickness using a diamond knife ultramicrotome (ULTRACUT UCT, Leica, installed at Eulji University, Seongnam, Republic of Korea), and then placed on a holey carbon TEM grid. Hundreds of bright-field lattice fringe images with corresponding selected area electron diffraction (SAED) patterns and EDS spectra were recorded for smectite clay minerals and then analysed using Digital MicrographTM software (Version 3.22. 1461. 0, Gatan Inc., Pleasanton, CA, USA). The EDS spectra were quantified to measure the elemental composition of smectite packets based on 20 oxygen atoms and 4 hydroxyl groups. The mechanical error range for the TEM-EDS quantification (wt. %) was approximately <0.5%. To reduce operational error, ~300 smectite packets were measured on the samples.

### Reconstruction of summer sea-surface temperature (SSST)

SSSTs for interglacial sediments at Site U1533 spanning MIS-15 to MIS-5 were calculated from temperature-dependent morphological variations (i.e., rectangularity) of valves of the diatom species *Fragilariopsis kerguelensis*^81–83^ and are given in Supplementary Data S5. Details of the applied method are provided in Ruggiero et al.^45^ and Mastro^84^. In total, 42 samples from the late Pleistocene–Holocene section of Site U1533 were investigated, but because of the limited number of *F. kerguelensis* valves present in the samples, measurements for *F. kerguelensis* valves from sediment intervals within each MIS were binned to create a single representative *F. kerguelensis* population for each individual interglacial period from MIS-15 to MIS-5, including glacial MIS-14^84^. Between 62 and 377 rectangularity measurements could be carried out for each analysed MIS^84^. SSST calibration followed Kloster et al.^81^, using modern SSSTs^85^. A recent study applied the same SSST reconstruction at high sample resolution to both modern and MIS-5 sediments from the Amundsen Sea rise, including Site U1533^45^.

## Supplementary information

Supplementary Information

Description of Additional Supplementary File

Supplementary Data S1

Supplementary Data S2

Supplementary Data S3

Supplementary Data S4

Supplementary Data S5

Transparent Peer Review file

## Data Availability

All data generated for this study are provided as Supplementary Data S1–S5. The down-core clay mineral assemblage data for IODP sites U1533 and U1532, as well as the clay mineral data from the seafloor-surface sediment samples collected on behalf of the THOR project, are also available from the NERC Polar Data Centre (PDC) at the British Antarctic Survey (Cambridge, UK). The datasets include clay mineral data from Hole U1533A (https://doi.org/10.5285/287c6403-9f9f-45f9-a34e-49e69f870b99), Hole U1533D (https://doi.org/10.5285/b86984d2-8d73-4731-bad7-701a8cb43534), Hole U1532A (https://doi.org/10.5285/43a4fb6f-62b2-4ed8-96b9-6b8827dbe15a) and seafloor-surface sediments collected from the vicinity of Thwaites Glacier and Pine Island Glacier (https://doi.org/10.5285/e8198dd3-ea60-4a4c-9975-d9c1695820fe). The bulk of the shipboard-collected core data from IODP Exp. 379 (including core photos) and the onshore collected XRF-scanner data are accessible at https://zenodo.org/communities/iodp (for a list of available datasets, see https://publications.iodp.org/proceedings/379/datasets.html).
