# Supplementary material for: Repeated loss of the West Antarctic Ice Sheet’s Amundsen Sea sector during late Pleistocene interglacials
Source: Nat Commun. 2026 Nov 9;17:10450. doi: 10.1038/s41467-026-77004-2 (PMC13748572; doi:10.1038/s41467-026-77004-2)
Supplement: Supplementary file 1 [file 41467_2026_77004_MOESM1_ESM.pdf]

Supplementary Information for

Repeated loss of the West Antarctic Ice Sheet's Amundsen Sea sector during late Pleistocene interglacials

Claus-Dieter Hillenbrand *et al.*

Corresponding author: Claus-Dieter Hillenbrand; email: hilc@bas.ac.uk

This Supplementary Information includes:

Supplementary Text

Supplementary Figures S1-S3

Supplementary References (1-105)

Other Supplementary Information for this manuscript includes:

Supplementary Data S1-S5

Supplementary Data Captions S1-S5

Supplementary Text

Oceanographic setting of the study area and possible role of bottom-current transport of clay-sized detritus from the Ross Sea within Antarctic Bottom Water (AABW)

In contrast to the continental shelf and upper slope of the Amundsen Sea[1-3], the water column structure above its continental rise has not been a focus of oceanographic research over recent decades and, thus, is less well studied. The deep and bottom water masses dominating the water column above the Amundsen Sea continental rise are Upper Circumpolar Deep Water (UCDW) and Lower Circumpolar Deep Water (LCDW)[4], referred to as "CDW" south of the Antarctic Polar Front, where the IODP Exp. 379 and PC494 core sites are located[4-6]. A water-column profile collected with a temperature probe at Site U1533 during Exp. 379 showed that the potential temperature (θ) of deep and bottom waters ranges from a maximum of +2.06 °C at around 370 m water depth to +0.17 °C just above the seafloor at 4162 m water depth[5], while a conductivity-temperature-depth (CTD) cast taken ~110 km to the NW of site PC494 during cruise JR179 gave a θ of +0.03 °C for bottom water at a similar water depth of 4218 m[7]. Classical Antarctic Bottom Water (AABW) has a θ of -0.7 °C[8], and according to this AABW definition seabed areas around Antarctica bathed by bottom waters with θ >0 °C, such as the study area, would only be influenced by CDW but not AABW[9,10]. According to a more recent, alternative AABW definition based on a neutral density (γ_n) >28.27 kg/m^3 (ref. 11), the studied core sites may just be located within the influence of a thin layer of AABW originating from the Ross Sea[11-13] that, like other deep and bottom waters around Antarctica, including the sub-Antarctic Pacific sector, has warmed and freshened over the last three decades[12-14].

Even if the study area has been influenced by some eastward flow of AABW, the observed glacial-interglacial changes in clay mineral assemblages in the investigated cores cannot be explained by changes in AABW outflow from the Ross Sea and associated long-distance current transport from this ice-sheet sector. First, smectite contents in seafloor-surface and core-top sediments from the Ross Sea shelf (normalised to 100% clay mineral content) are on average ~30–35% and only locally as high as (or higher than) in the interglacial sediments from the eastern Amundsen Sea continental rise[15,16], and they are (often much) less than ~20% in late Pleistocene sediments from the Ross Sea continental slope[17,18]. This conflicts with interglacial transport of smectite-enriched clay from the Ross Sea to the Amundsen Sea via deep-sea currents, which is also evident from the fact that the smectite contents in surface sediments on the Amundsen Sea continental rise actually decrease from East to West (Fig. 1b).

On the other hand, the Ross Sea drainage sector is an unlikely source for kaolinite supply to the Amundsen Sea continental rise during glacial periods because kaolinite content in the majority of core-top sediments from the Ross Sea shelf is on average less than 7%[15,16]. Two local kaolinite sources, where kaolinite contents in seafloor-surface sediments can reach up to ~14%, are present directly offshore from the easternmost Ross Ice Shelf[16] and in a small bay within Terra Nova Bay on the western Ross Sea shelf[19]. However, AABW and CDW flow would transport detritus derived from these source areas westward along the Ross Sea continental slope and rise, but not eastward[20]. This is consistent with the observation that kaolinite contents recorded in late Pleistocene sediments from the slope and rise of the central and western Ross Sea are typically low and rarely, if at all, reach maxima up to ~15%[17,18,21]. Crucially, down-core analyses of clay mineral assemblages have

shown that the kaolinite contents in sediments deposited on the central Ross Sea continental slope during late Pleistocene glacial periods are lower than in the interglacial sediments, exceeding not more than ~6%[17].

Second, AABW outflow is known to have weakened in other sectors of the Antarctic continental margin during late Pleistocene glacial periods[22,23]. Similarly, the formation of dense shelf waters, i.e., precursor water masses for AABW, and production of AABW itself on the Ross Sea continental margin are assumed to have been reduced during peak glacial periods of the late Pleistocene[24], including the Last Glacial Maximum (LGM; ~23–19 ka before present)[23], while an observed coarsening of glacial sediments deposited on the Ross Sea continental slope was attributed to an intensification of the westward flowing Antarctic Slope Current (ASC) rather than increased outflow of dense shelf waters[20]. Thus, any influence of AABW outflow from the Ross Sea on sediment deposition on the Amundsen Sea continental rise would have been even weaker during glacial times than today. In summary, any intensified or reduced advection of Ross Sea derived clay-sized detritus within AABW during glacials or interglacials is no feasible scenario for explaining the glacial-interglacial changes in clay mineral assemblages recorded on the eastern Amundsen Sea continental rise (Figs. 3, 4).

<u>Previous geological and biological records recovered from below and proximal to the West Antarctic Ice Sheet (WAIS) to test the hypothesis of its collapse during the last 800 kyr</u>

In the following, we present a short review of proxy evidence from sediments and ice cores as well as genetic evidence from biological records for/against WAIS collapse during the last 800 kyr. For a focus on the last interglacial period of MIS-5e (130–115 ka), the reader is also referred to the recently published review by Bentley & Wolff[25],

which additionally takes into account findings from far-field sea-level records and numerical models.

Subglacial sediments

Findings of late Pleistocene marine diatoms and relatively high concentrations of the cosmogenic nuclide beryllium-10 (^{10}Be) in till samples recovered at site Upstream B (UpB) from the bed of the Whillans Ice Stream that drains the WAIS into the Ross Ice Shelf had been interpreted as evidence for at least one WAIS collapse during the last ~750 kyr[26]. Marine Isotope Stage (MIS) 11 (424–374 ka), when global sea level was 6–13 m higher than today[27], and/or MIS-5e, when sea level was probably 3.6–8.7 m above present[27-31], were considered as the most likely time periods for the WAIS collapse[26], even though large regional variations in relative MIS-5e highstands have been reported over the last few years[32-34]. More recently published subglacial sediment records from the Ross Sea drainage sector have provided evidence that the WAIS grounding line had temporarily retreated to the UpB and other subglacial sampling sites during the early Holocene and subsequently re-advanced[35-37]. These new findings allude to the possibility that marine organisms – and adhering ^{10}Be – could have been advected there at the beginning of the previous and earlier interglacial periods without the necessity for a partial or total WAIS collapse. Similar early Holocene ice sheet retreat upstream of the modern grounding line with subsequent re-advance has also been reported for other Antarctic drainage sectors[38], including the Amundsen Sea sector of the WAIS[39].

Marine sediment cores

At Ocean Drilling Program (ODP) Leg 178 Site 1096 on the western Antarctic Peninsula continental rise[40,41], a high-resolution chronology was developed for its late Pleistocene–Holocene section using the relative magnetic palaeointensity (RPI)

record of the sediments[42]. Silt-sized detritus with a geochemical provenance signature (i.e., a combination of neodymium–strontium–lead [Nd–Sr–Pb] isotopes and trace elements) indicative for subglacial erosion below the WAIS was detected in sediments assigned to the end of glacial MIS-6 (191–130 ka) and MIS-5d to MIS-1 (115–0 ka), respectively[42]. In contrast, this provenance signature was absent from deposits assigned to the last interglacial MIS-5e, which contained silt exclusively derived from the Antarctic Peninsula and eroded by the Antarctic Peninsula Ice Sheet[42]. The lack of silt particles eroded by the WAIS was interpreted to be the result of WAIS collapse during MIS-5e[42].

However, the depth assignments of tephra layers[43] in the two drill holes at Site 1096 used to constrain the RPI age model and, thus, the age range of the alleged MIS-5e sediments[42] are neither consistent with the shipboard derived[40] nor the post-cruise developed[41] composite depth scales. These depth scales place the sediments with the exclusive Antarctic Peninsula provenance fingerprint into glacial MIS-6. Moreover, the new RPI-based age model for Site 1096[42] is called into question. This is because RPI age models published for cores in the same region[44] have subsequently been shown to deviate at glacial terminations, including the MIS-6/5e transition, from age models derived from stable oxygen isotope ($\delta^{18}O$) data analysed on planktic foraminifera and tephrochronological constraints for the same cores[45]. The discrepancies probably result from early diagenetic processes that affected the magnetic properties of the sediments[45]. Also according to these new age models[45], the purely Antarctic Peninsula derived sediments reported by Carlson et al.[42] would have been deposited during glacial MIS-6. At this time, grounded ice streams draining the Antarctic Peninsula Ice Sheet had advanced to the nearby shelf break and delivered vast amounts of Antarctic Peninsula derived glacigenic debris to the

continental slope and rise[45-49], overwhelming any provenance signal of WAIS derived silt. The latter could only have been transported NE-wards to Site 1096 by surface water currents or icebergs[46,47,49] because Site 1096 is – and has been – affected by a generally SW-ward flowing bottom current[45,50].

Proxies considered to be sensitive for major ice-sheet retreat, such as accumulation rates of coarse-grained iceberg-rafted debris (IRD)[22,51], were analysed by Hillenbrand et al.[52] on a condensed sedimentary sequence recovered at gravity core site PS2547-3 on the western Amundsen Sea continental slope (Fig. S1). The core had been dated at 5 cm-depth resolution using planktic and benthic foraminifera $\delta^{18}O$ stratigraphy constrained by magnetostratigraphy[52] (cf. Williams et al.[53], who analysed piston core PC493 from the same location at 1 cm-depth resolution). In accordance with earlier published ice-sheet model results[54], the authors expected to detect a depositional anomaly, such as an unusual IRD accumulation maximum, during an interglacial period to be indicative of a WAIS collapse. However, they failed to find such a depositional anomaly during the last 800 kyr and even the last 1.8 Myr[52].

Another study carried out a multi-proxy investigation on a piston core collected at site PS58/254-1 from Resolution Drift on the eastern Amundsen Sea continental rise (Fig. S1) to search for any depositional anomaly indicative of a WAIS collapse[55]. This sediment record was dated with a combination of lithostratigraphy and magnetostratigraphy[55], subsequently corroborated by diatom biostratigraphy[56], and has a higher temporal resolution than the previously investigated core PS2547-3[52]. The only significant depositional anomaly identified in the core from Resolution Drift was glacial MIS-14 (563–533 ka), when, like in other sectors of the Antarctic continental margin, the Southern Ocean, and other parts of the world, sediments

more typical for late Pleistocene interglacials had been deposited[55]. Consequently, the whole period from interglacial MIS-15 to MIS-13 (621–478 ka) could be considered as a single, prolonged warm period[55] (cf. references 57,58). The authors concluded that, if the WAIS had collapsed during the past 800 kyr, the period from MIS-15 to MIS-13 was the most likely time[55].

Ice core records

In the Weddell Sea drainage sector, a WAIS collapse during MIS-5e was inferred by Turney et al.[59] from a horizontal ice core recovered in the Patriot Hills, located at the northern flank of the Ellsworth-Whitmore Mountains (Fig. S1) and directly south of the Ronne Ice Shelf. Hydrogen isotope (δD) data analysed on this record indicate warmer conditions and reduced sea-ice cover nearby just before the MIS-6/5e transition[59]. Nearly all ice of MIS-5e age was missing from the core, which was interpreted as consequence of major ice-sheet surface draw-down combined with strong wind-scouring resulting from widespread deglaciation of the Weddell Sea drainage sector of the WAIS[59]. However, according to the authors of this study, the absence of most MIS-5e-old ice leaves room for alternative explanations for the hiatus in the investigated ice core[59]. Like other unconformities in this blue-ice record, the MIS-5e hiatus could possibly result from a dominance of wind erosion alone[60]. Furthermore, a major reduction in sea-ice cover in the Scotia–Weddell Sea region during the MIS-6/5e transition and MIS-5e, inferred from both diatom assemblages in marine sediment cores[61] and $\delta^{18}O$ and δD data from East Antarctic ice cores[62], may explain the sea-ice minimum at the MIS-6/5e transition archived in the blue-ice record.

According to a subsequently published ice-sheet modelling study the apparent hiatus in the horizontal ice core may record a period of extreme layer thinning during MIS-

5e[63]. The thinning, resulting from a combination of grounding-line retreat and ice-flow acceleration in the SW Weddell Sea Embayment with glacioisostatic uplift in response to both this retreat and the collapse of WAIS's Amundsen Sea drainage sector, could have allowed stratigraphically separated isochrones to eventually intersect in the blue-ice area[63]. Consistent with these ice-sheet model simulations[63], water isotope and sea-salt data from an ice core recently drilled at the Skytrain Ice Rise, located just to the NE of the Patriot Hills, were interpreted to indicate that the WAIS may have retreated in the Weddell Sea sector during MIS-5e but that the Ronne Ice Shelf remained in place and had an extent comparable to today[64]. In contrast, a change in dust provenance across the MIS-6 to MIS-5e transition recorded in blue ice from the Allan Hills in the SW Ross Sea sector has recently been attributed to major retreat of the Ross Ice Shelf and the WAIS in the Ross Sea sector during MIS-5e[65]. If, however, the scenarios of limited WAIS loss are confirmed, this would require significant ice retreat in East Antarctica to explain the total Antarctic contribution to the global sea-level highstand during MIS-5e, most likely in the marine-based Wilkes Subglacial Basin[66-68].

Biological evidence

Bryozoans are sessile benthic organisms common on the Antarctic continental shelf, and their young are short-lived benthic larvae with no or only very limited capacity for widespread, rapid dispersal[69]. Similarity between modern bryozoan assemblages from the continental shelves of the Amundsen, Bellingshausen, Weddell and Ross seas had previously been interpreted to indicate a connection between these ocean sectors via Trans-Antarctic seaways that had opened up in response to a WAIS collapse during the recent geological past[69,70]. These studies could only estimate the timing of the last faunal exchange, favouring a late Pleistocene seaway opening[69,70].

A comparison of the population genetics of a circum-Antarctic benthic octopus species sampled in various shelf sectors around West and East Antarctica detected historic signals of gene flow between the populations[71]. The study concluded that the observed similarities in octopus DNA, especially between populations from the Amundsen, Ross and Weddell Sea shelves, were only possible due to the opening of West Antarctic seaways, with the applied demographic model suggesting that the last WAIS collapse occurred during MIS-5 (130–71 ka)[71]. However, long-distance dispersal of the investigated octopus species, possibly caused by current-transport of eggs, had previously been reported[72]. Given the existence of large-scale circulation systems, such as the clockwise-flowing Ross Gyre which connects the Amundsen Sea and Ross Sea continental shelves today[73], long-distance gene flow between various shelf sectors may be a possibility. Future genetic investigations need to verify, whether the analysis of Lau et al.[71] will withstand further scrutiny and the test of time[74].

Supplementary Figures S1-S3

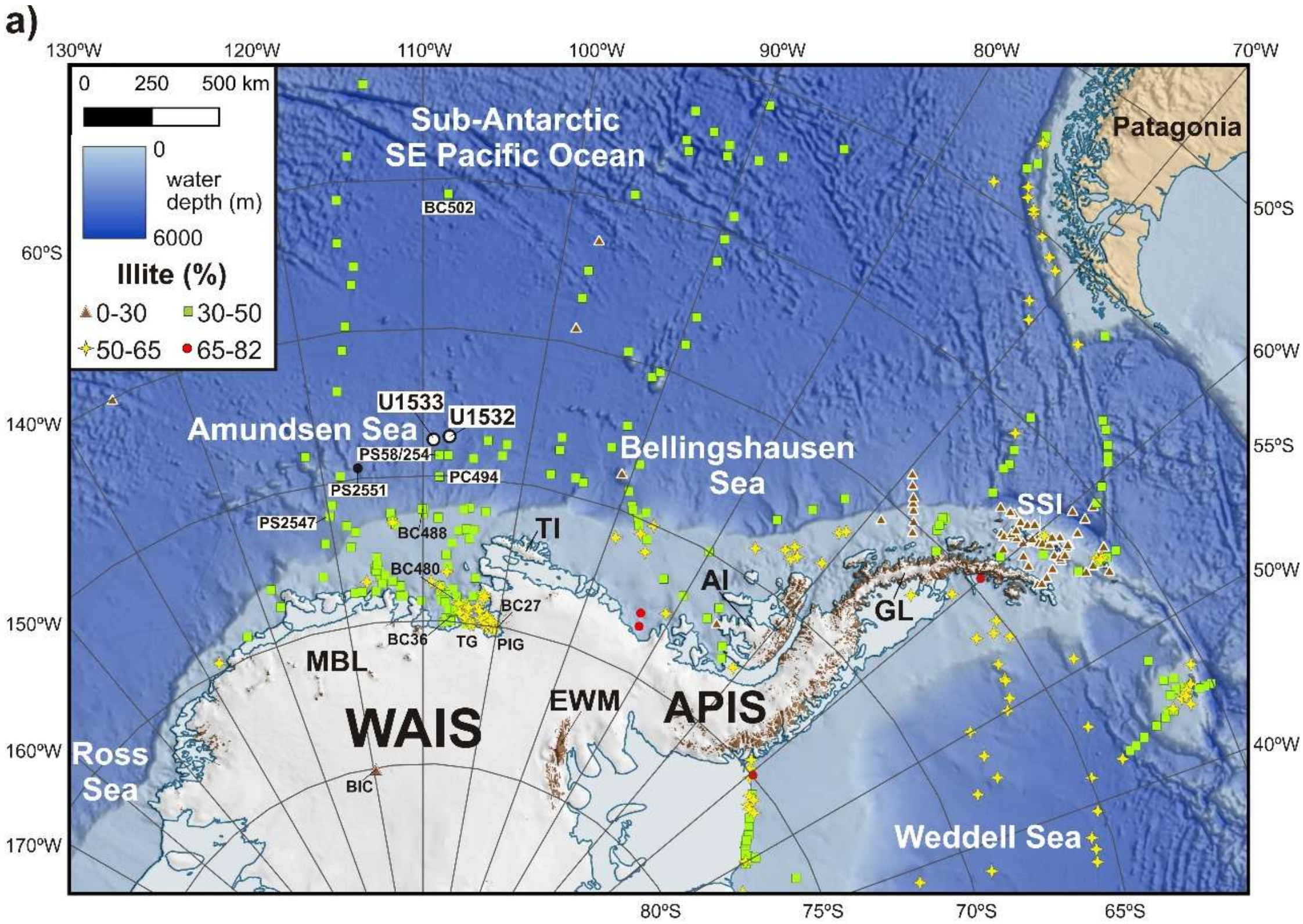

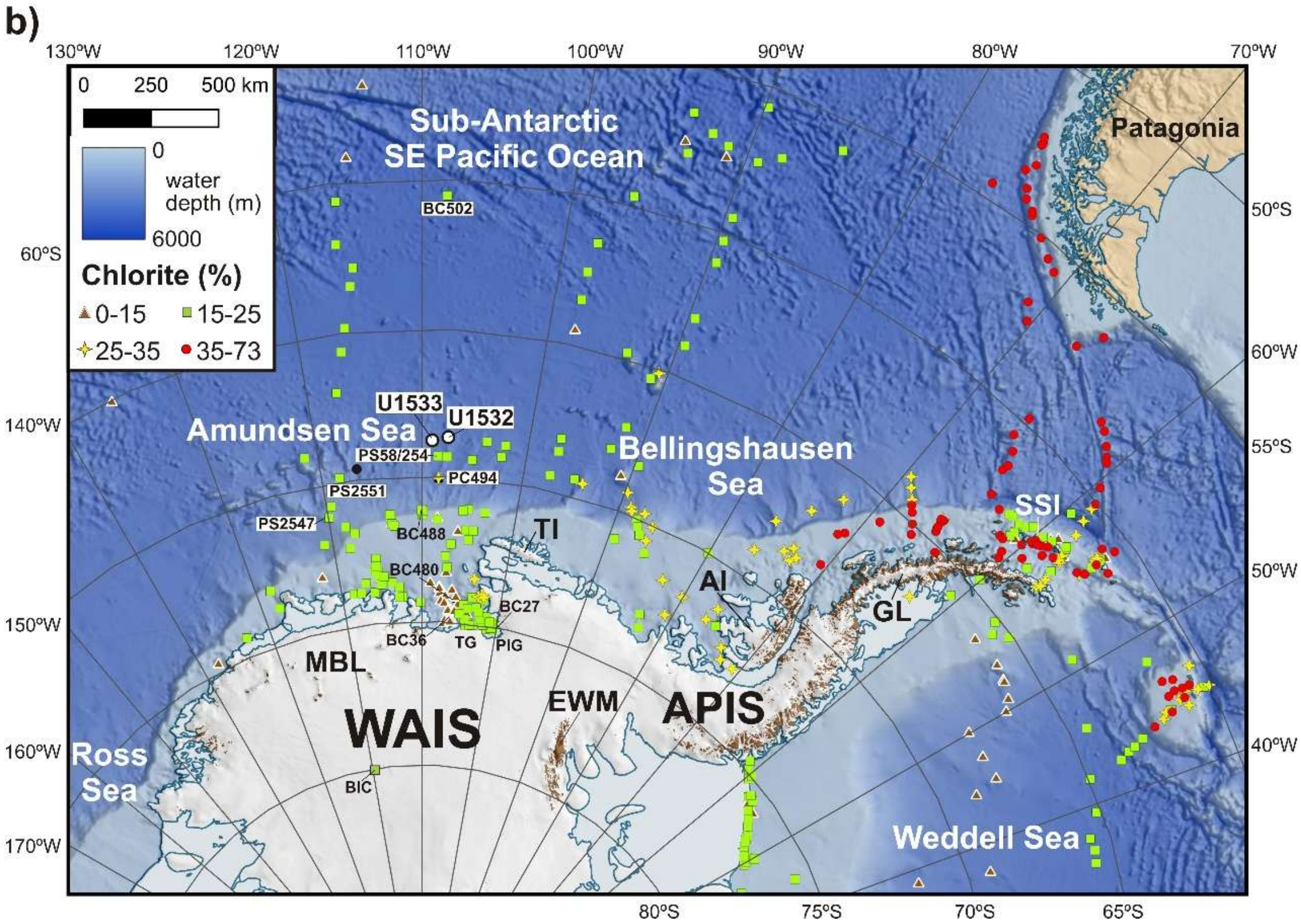

Fig. S1: Illite and chlorite contents in contemporary sediments from the SE Pacific and SW Atlantic sectors of the Southern Ocean. a) Bathymetric map

showing illite content in the clay fraction (<2 μm) of latest MIS-1 [14–0 ka] to modern seafloor-surface sediments and basal glacigenic debris of the Byrd Ice Core (BIC). Clay mineral data from this study and references 22,45,52,75-93 (Supplementary Data S2). Map generated with Quantarctica[94], using data from IBCSO v.1.0[95] and SCAR ADD v.7.0[96]. AI: Alexander Island, APIS: Antarctic Peninsula Ice Sheet, EWM: Ellsworth-Whitmore Mountains, GL: Graham Land, MBL: Marie Byrd Land, PIG: Pine Island Glacier, SSI: South Shetland Islands, TG: Thwaites Glacier, TI: Thurston Island, WAIS: West Antarctic Ice Sheet. **b)** Same as in Fig. S1a, but for chlorite content.

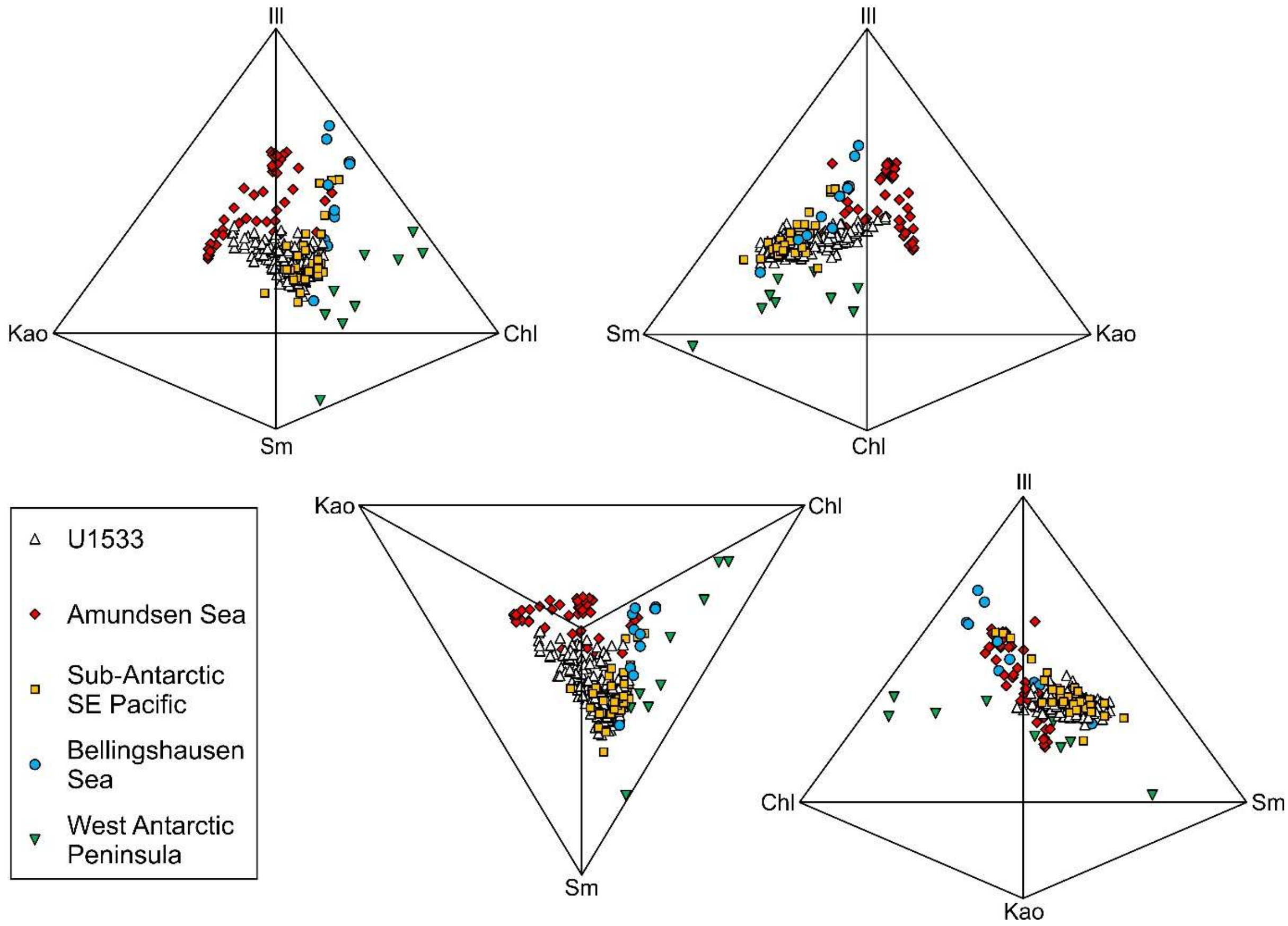

Fig. S2: Tetrahedral diagrams displaying the relationship between clay mineral assemblages of West Antarctic and sub-Antarctic source areas and in glacial and interglacial sediments at Site U1533 on the eastern Amundsen Sea continental rise. Contemporary surface sediments from near-coastal sample locations on the West Antarctic continental shelf and sampling sites in the sub-Antarctic Pacific Ocean considered to characterise the individual source areas are marked in Supplementary Data S2. Data for Site U1533 span the late Pleistocene to Holocene (773–0 ka; Fig. 3 in main paper). Chl: chlorite, Ill: illite, Kao: kaolinite, Sm: smectite. The 3D-visualisation of the clay mineral assemblages as tetrahedral diagrams used the 'Tetra-Plot' Microsoft Excel spreadsheet published by Cucciniello[97].

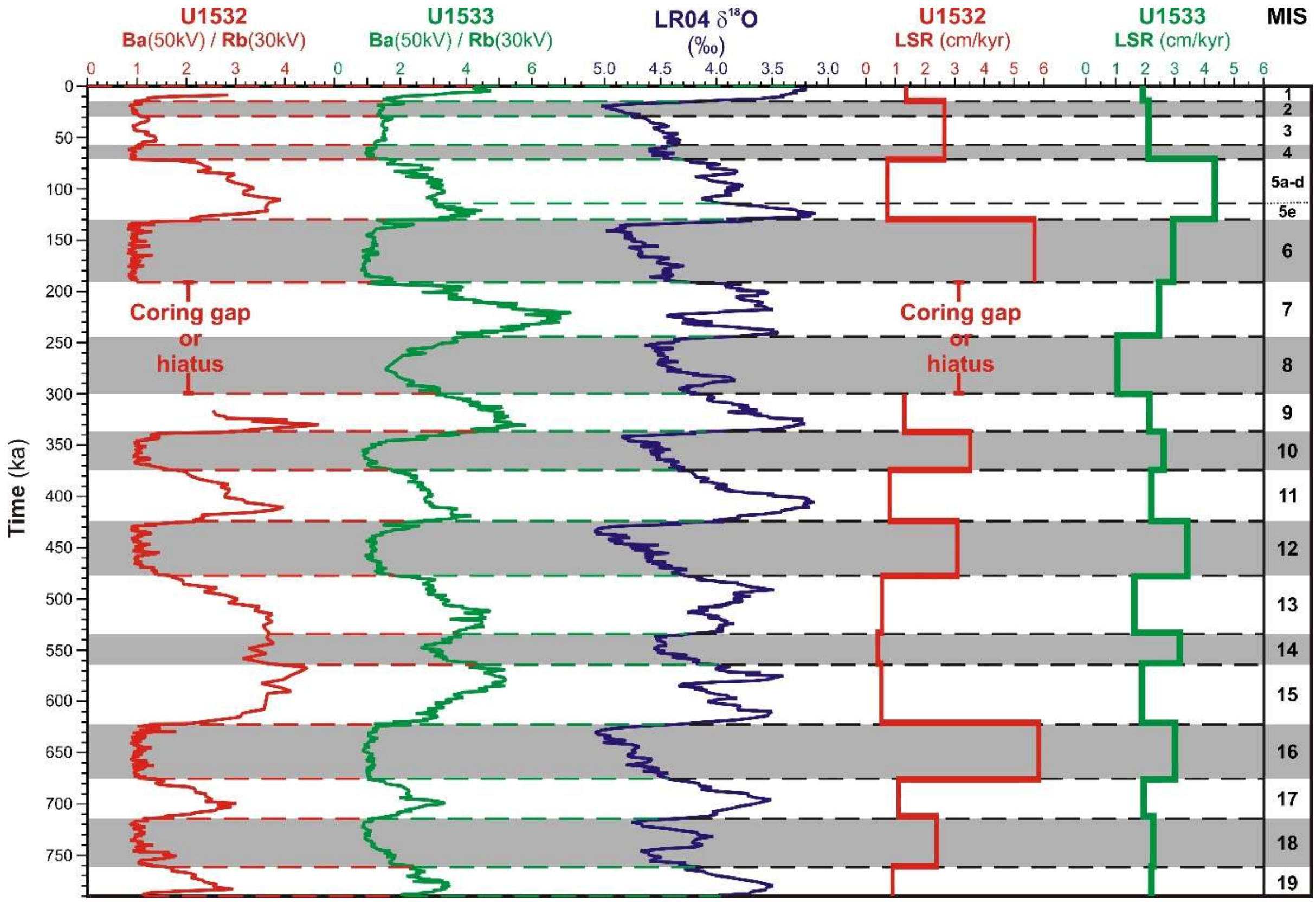

Fig. S3: Correlation between barium/rubidium (Ba/Rb) ratios and the LR04 global oxygen isotope stack to develop age models for sites U1532 and U1533 and linear sedimentation rates. Bases and tops of Ba/Rb maxima were correlated with the beginnings and ends of interglacial Marine Isotope Stages (MIS) in the LR04 benthic foraminifera $\delta^{18}O$ stack[98]. Age-depth tie lines (Supplementary Data S1) are illustrated by the dashed lines, and grey shaded intervals mark glacial Marine Isotope Stages (MIS). Linear sedimentation rates (LSRs) shown are marked with an asterisk in Supplementary Data S1 and highlight glacial-interglacial variabilities at and between the two sites.

Supplementary References

1. A. Jenkins et al., Decadal ocean forcing and Antarctic Ice Sheet response: Lessons from the Amundsen Sea. *Oceanography* **29 (4)**, 106-117 (2016); https://doi.org/10.5670/oceanog.2016.103.
2. S. Jacobs et al., The Amundsen Sea and the Antarctic Ice Sheet. *Oceanography* **25 (3)**, 154-163 (2012); http://dx.doi.org/10.5670/oceanog.2012.90.
3. K.J. Heywood et al., Between the devil and the deep blue sea: The role of the Amundsen Sea continental shelf in exchanges between ocean and ice shelves. *Oceanography* **29 (4)**, 118-129 (2016); https://doi.org/10.5670/oceanog.2016.104.
4. A.H. Orsi, T. Whitworth III, W.D. Nowlin Jr., On the meridional extent and fronts of the Antarctic Circumpolar Current. *Deep-Sea Res. I* **42**, 641-673 (1995).
5. K. Gohl, J.S. Wellner, A. Klaus, Expedition 379 Scientists, *Amundsen Sea West Antarctic Ice Sheet History*. *Proc. IODP* **379** (International Ocean Discovery Program, College Station, Texas, U.S.A., 2021); https://doi.org/10.14379/iodp.proc.379.2021.
6. J.R. Horrocks, *The formation and Late Quaternary palaeoenvironmental history of sediment mounds in the Amundsen Sea, West Antarctica* (Durham theses, Durham University, Durham, UK, 2018), 338 pp.; http://etheses.dur.ac.uk/12659/.
7. P. Carter, D. Vance, C.-D. Hillenbrand, J.A. Smith, D.R. Shoosmith, The neodymium isotopic composition of waters masses in the eastern Pacific sector of the Southern Ocean. *Geochim. Cosmochim. Acta* **79**, 41-59 (2012).
8. A.W. Mantyla, J.L. Reid, Abyssal characteristics of the World Ocean waters. *Deep-Sea Res.* **30**, 805-833 (1983).

9. S.G. Purkey, G.C. Johnson, Antarctic Bottom Water warming and freshening: Contributions to sea level rise, ocean freshwater budgets, and global heat gain. *J. Clim.* **26**, 6105-6122 (2013).
10. Y. Choi, S. Nam, East–west contrasting changes in Southern Indian Ocean Antarctic Bottom Water salinity over three decades. *Sci. Repts.* **12**, 12175 (2022); https://doi.org/10.1038/s41598-022-16331-y.
11. A.H. Orsi, G.C. Johnson, J.L. Bullister, Circulation, mixing, and production of Antarctic Bottom Water. *Prog. Oceanogr.* **43**, 55-109 (1999).
12. S.G. Purkey et al., A synoptic view of the ventilation and circulation of Antarctic Bottom Water from chlorofluorocarbons and natural tracers. *Annual Rev. Mar. Sci.* **10**, 503-527 (2018); https://doi.org/10.1146/annurev-marine-121916-063414.
13. Y. Bai, L. Zhao, J. Xiao, S. Lin, Contraction and warming of Antarctic Bottom Water in the Amundsen Sea. *Acta Oceanol. Sin.* **41**, 68-79 (2022); https://doi.org/10.1007/s13131-021-1829-8.
14. G.C. Johnson, S.G. Purkey, N.V. Zilberman, D. Roemmich, Deep Argo quantifies bottom water warming rates in the Southwest Pacific Basin. *Geophys. Res. Lett.* **46**, 2662-2669 (2019); https://doi.org/10.1029/2018GL081685.
15. J.T. Andrews, W. LeMasurier, Resolving the argument about volcanic bedrock under the West Antarctic Ice Sheet and implications for ice sheet stability and sea level change. *Earth Planet. Sci. Lett.* **568**, 117035 (2021).
16. L. Wu et al., Grain-size, coarse fraction lithology and clay mineral compositions of surface sediments from Ross Sea, Antarctica: implications for their provenance and delivery mode. *Front. Mar. Sci.* **10,** 1324391 (2024); https://doi.org/10.3389/fmars.2023.1324391.

17. S. Ha, B.-K. Khim, H.G. Cho, E. Colizza, Origin of clay minerals of core RS14-GC2 in the continental slope to the East of the Pennell-Iselin Bank in the Ross Sea, Antarctica. *J. Miner. Soc. Korea* **31**, 1-12 (2018).

18. S. Ha et al., Estimation of sediment provenance using clay mineral composition in the Central Basin of the Ross Sea continental margin, Antarctica. *Ocean Polar Res.* **41**, 265-274 (2019); http://dx.doi.org/10.4217/OPR.2019.41.4.265.

19. J. Jung et al., Multibeam bathymetry and distribution of clay minerals on surface sediments of a small bay in Terra Nova Bay, Antarctica. *Minerals* **11**, 72 (2021); https://doi.org/10.3390/min11010072.

20. S. Kim et al., Late Pleistocene paleoceanographic changes in the Ross Sea – Glacial-interglacial variations in paleoproductivity, nutrient utilization, and deep-water formation. *J. Quat. Sci. Rev.* **239**, 106356 (2020); https://doi.org/10.1016/j.quascirev.2020.106356.

21. J. Jung et al., Clay mineralogical characteristics and origin of sediments deposited during the Pleistocene in the Ross Sea, Antarctica. *J. Miner. Soc. Korea* **32**, 163-172 (2019).

22. B. Diekmann et al., "Terrigenous sediment supply in the polar to temperate South Atlantic: Land-ocean links of environmental changes during the Late Quaternary" in *The South Atlantic in the Late Quaternary: Reconstruction of Material Budgets and Current Systems*, G. Wefer, S. Mulitza, V. Ratmeyer, Eds. (Springer-Verlag Berlin-Heidelberg-New York-Tokyo, 2003), pp. 375-399.

23. S. Ha et al., Glaciomarine sediment deposition on the continental slope and rise of the central Ross Sea since the Last Glacial Maximum. *Mar. Geol.* **445**, 106752 (2022); https://doi.org/10.1016/j.margeo.2022.106752.

24. M. Bollen et al., Pleistocene oceanographic variability in the Ross Sea: A multi-proxy approach to age model development and paleoenvironmental analyses. *Global Planet. Change* **216**, 103901 (2022); https://doi.org/10.1016/j.gloplacha.2022.103901.

25. M.J. Bentley, E.W. Wolff, Past interglacial West Antarctic Ice Sheet collapse: a critical review of evidence and approaches. *Quat. Sci. Rev.* **387**, 110025 (2026); https://doi.org/10.1016/j.quascirev.2026.110025.

26. R.P. Scherer et al., Pleistocene collapse of the West Antarctic ice sheet. *Science* **281**, 82-85 (1998).

27. A. Dutton et al., Sea-level rise due to polar ice-sheet mass loss during past warm periods. *Science* **3491**, doi:10.1126/science.aaa4019 (2015); http://dx.doi.org/10.1126/science.aaa4019.

28. P. Blanchon, A. Eisenhauer, J. Fietzke, V. Liebetrau, Rapid sea-level rise and reef back-stepping at the close of the last interglacial highstand. *Nature* **458**, 881-884 (2009).

29. M.J. O'Leary et al. Ice sheet collapse following a prolonged period of stable sea level during the last interglacial. *Nat. Geosci.* **6**, 796-800 (2013).

30. R.E. Kopp, F.J. Simons, J.X. Mitrovica, A.C. Maloof, M. Oppenheimer, Probabilistic assessment of sea level during the last interglacial stage. *Nature* **462**, 863-867 (2009).

31. R.L. Barnett et al., Constraining the contribution of the Antarctic Ice Sheet to Last Interglacial sea level. *Sci. Adv.* **9**, eadf0198 (2023); https://www.science.org/doi/10.1126/sciadv.adf0198.

32. B. Dyer et al., Sea-level trends across the Bahamas constrain peak last interglacial ice melt. *Proc. Natl. Acad. Sci.* 118, e2026839118 (2021); https://doi.org/10.1073/pnas.2026839118.

33. R.C. Creel, J. Austermann, Glacial isostatic adjustment driven by asymmetric ice sheet melt during the Last Interglacial causes multiple local sea-level peaks. *Geology* **53**, 253-258 (2025); https://doi.org/10.1130/G52483.1.

34. K. Vyverberg et al., Episodic reef growth in the Last Interglacial driven by competing influence of polar ice sheets to sea level rise. *Sci. Adv.* **11**, eadu3701 (2025); https://doi.org/10.1126/sciadv.adu3701.

35. J. Kingslake et al., Extensive retreat and re-advance of the West Antarctic Ice Sheet during the Holocene. *Nature* **558**, 430-434 (2018).

36. S.U. Neuhaus et al., Did Holocene climate changes drive West Antarctic grounding line retreat and readvance? *Cryosphere* **15**, 4655-4673 (2021); https://doi.org/10.5194/tc-15-4655-2021.

37. R.A. Venturelli et al., Constraints on the timing and extent of deglacial grounding line retreat in West Antarctica. *Am. Geophys. Union Advances* **4**, e2022AV000846 (2023); https://doi.org/10.1029/2022AV000846.

38. J.S. Johnson et al., Existing and potential evidence for Holocene grounding line retreat and readvance in Antarctica. *Cryosphere* **16**, 1543-1562 (2022); https://doi.org/10.5194/tc-16-1543-2022.

39. G. Balco et al., Reversible ice sheet thinning in the Amundsen Sea Embayment during the Late Holocene. *Cryosphere* **17**, 1787-1801 (2023); https://doi.org/10.5194/tc-17-1787-2023.

40. P.F. Barker, A. Camerlenghi, G.D. Acton et al., *Proceedings Ocean Drilling Program Initial Report 178* [CD-ROM] (Ocean Drilling Program, Texas A&M University, College Station, Texas, U.S.A., 1999).

41. P.F. Barker, A. Camerlenghi, G.D. Acton, A.T.S. Ramsay et al., *Proceedings Ocean Drilling Program Scientific Results 178* [CD-ROM] (Ocean Drilling Program, Texas A&M University, College Station, Texas, U.S.A., 2002).

42. A.E. Carlson, B.L. Beard, R.G. Hatfield, M. Laffin, Absence of West Antarctic-sourced silt at ODP Site 1096 in the Bellingshausen Sea during the last interglaciation: Support for West Antarctic ice-sheet deglaciation. *J. Quat. Sci. Rev.* **261**, 106939 (2021).

43. C.-D. Hillenbrand et al., Volcanic time-markers for Marine Isotopic Stages 6 and 5 in Southern Ocean sediments and Antarctic ice cores: implications for tephra correlations between palaeoclimatic records. *J. Quat. Sci. Rev.* **27**, 518-540 (2008).

44. J.E.T. Channell, C. Xuan, S.J. Crowhurst, D.A. Hodell, R.D. Larter, Relative paleointensity (RPI) and age control in Quaternary sediment drifts off the Antarctic Peninsula. *J. Quat. Sci. Rev.* **211**, 17-33 (2019).

45. C.-D. Hillenbrand et al., New multi-proxy data from West Antarctic drift sediments: implications for dating and interpreting Late Quaternary palaeoenvironmental records from the Antarctic margin. *J. Quat. Sci. Rev.* **257**, 106842 (2021); https://doi.org/10.1016/j.quascirev.2021.1068422021.

46. R.G. Lucchi et al., Mid-late Pleistocene glacimarine sedimentary processes of a high-latitude, deep-sea sediment drift (Antarctic Peninsula Pacific margin). *Mar. Geol.* **189**, 343-370 (2002).

47. C.J. Pudsey, A. Camerlenghi, Glacial-interglacial deposition on a sediment drift on the Pacific margin of the Antarctic Peninsula. *Antarct. Sci.* **10**, 286-308 (1998).

48. C.J. Pudsey, Sedimentation on the continental rise west of the Antarctic Peninsula over the last three glacial cycles. *Mar. Geol.* **167**, 313-338 (2000).

49. S. Rodrigues et al., Recognizing key sedimentary facies and their distribution in mixed turbidite–contourite depositional systems: The case of the Pacific margin of the Antarctic Peninsula. *Sedimentol.* **69**, 1953-1991 (2022).

50. C.-D. Hillenbrand et al., The present and past bottom-current flow regime around the sediment drifts on the continental rise west of the Antarctic Peninsula. *Mar. Geol.* **255**, 55-63 (2008).

51. M.E. Weber et al., Millennial-scale variability in Antarctic ice-sheet discharge during the last deglaciation. *Nature* **510**, 134-138 (2014).

52. C.-D. Hillenbrand, D.K. Fütterer, H. Grobe, T. Frederichs, No evidence for a Pleistocene collapse of the West Antarctic Ice Sheet from continental margin sediments recovered in the Amundsen Sea. *Geo-Mar. Lett.* **22**, 51-59 (2002).

53. T.J. Williams et al., Paleocirculation and ventilation history of Southern Ocean sourced deep water masses during the last 800,000 years. *Paleoceanogr. Paleoclimatol.* **34**, 833-852 (2019).

54. D.R. MacAyeal, Irregular oscillations of the West Antarctic Ice Sheet. *Nature* **359**, 29-32 (1992).

55. C.-D. Hillenbrand, G. Kuhn, T. Frederichs, Record of a Mid-Pleistocene depositional anomaly in West Antarctic sediments: an indicator for ice-sheet collapse? *J. Quat. Sci. Rev.* **28**, 1147-1159 (2009).

56. M.A. Konfirst, R.P. Scherer, C.-D. Hillenbrand, G. Kuhn, A marine diatom record from the Amundsen Sea - Insights into oceanographic and climatic response to the Mid-Pleistocene Transition in the West Antarctic sector of the Southern Ocean. *Mar. Micropal.* **92-93**, 40-51 (2012).

57. Q. Hao, L. Wang, F. Oldfield, Z. Guo, Extra-long interglacial in Northern Hemisphere during MISs 15-13 arising from limited extent of Arctic ice sheets in glacial MIS 14. *Sci. Repts.* **5**, 12103, doi:10.1038/srep12103 (2015).

58. P.D. Hughes, P.L. Gibbard, J. Ehlers, The “missing glaciations” of the Middle Pleistocene. *Quat. Res.* **96**, 161-183, https://doi.org/10.1017/qua.2019.76 (2020).

59. C. Turney et al., Early Last Interglacial ocean warming drove substantial ice mass loss from Antarctica. *Proc. Natl. Acad. Sci.* **117**, 3996-4006 (2020).

60. K. Winter et al., Assessing the continuity of the blue ice climate record at Patriot Hills, Horseshoe Valley, West Antarctica. *Geophys. Res. Lett.* **43**, 2019-2026 (2016); https://doi.org/10.1002/2015GL066476.

61. M. Chadwick, X. Crosta, O. Esper, L. Thöle, K.E. Kohfeld, Compilation of Southern Ocean sea-ice records covering the last glacial-interglacial cycle (12–130 ka). *Clim. Past* **18**, 1815-1829 (2022); https://doi.org/10.5194/cp-18-1815-2022.

62. M.D. Holloway et al., Antarctic last interglacial isotope peak in response to sea ice retreat not ice-sheet collapse. *Nat. Commun.* **7**, 12293, doi:10.1038/ncomms12293 (2016).

63. N.R. Golledge et al., Retreat of the Antarctic Ice Sheet During the Last Interglaciation and Implications for Future Change. *Geophys. Res. Lett.* **48**, e2021GL094513 (2021); https://doi.org/10.1029/2021GL094513.

64. E.W. Wolff et al., The Ronne Ice Shelf survived the last interglacial. *Nature* **638**, 133-137 (2025); https://doi.org/10.1038/s41586-024-08394-w.

65. A.J. Carter et al., Diminished Ross Ice Shelf and West Antarctic Ice Sheet during Last Interglacial warming. *Nature Geosci.* (2026); https://doi.org/10.1038/s41561-026-01988-1.

66. M. Iizuka et al. Multiple episodes of ice loss from the Wilkes Subglacial Basin during the Last Interglacial. *Nat. Commun.* **14**, 2129 (2023); https://doi.org/10.1038/s41467-023-37325-y.

67. D.J. Wilson et al., Ice loss from the East Antarctic Ice Sheet during late Pleistocene interglacials. *Nature* **561**, 383-386 (2018); https://doi.org/10.1038/s41586-018-0501-8.

68. T. Blackburn et al., Ice retreat in Wilkes Basin of East Antarctica during a warm interglacial. *Nature* **583**, 554-558 (2020); https://doi.org/10.1038/s41586-020-2484-5.

69. D.K.A. Barnes, C.-D. Hillenbrand, Faunal evidence for a late quaternary trans-Antarctic seaway. *Global Change Biol.* **16**, 3297-3303, (2010); https://doi.org/10.1111/j.1365-2486.2010.02198.x.

70. D.G. Vaughan, D.K.A. Barnes, P.T. Fretwell, R.G. Bingham, Potential seaways across West Antarctica. *Geochem. Geophys. Geosys.* **12 (10)**, Q10004, (2011); https://doi.org/10.1029/2011GC003688.

71. S.C.Y. Lau et al., Genomic evidence for West Antarctic Ice Sheet collapse during the Last Interglacial. *Science* **382**, 1384-1389 (2023); https://doi.org/10.1126/science.ade0664.

72. J.M. Strugnell, P.C. Watts, P.J. Smith, A.J. Allcock, Persistent genetic signatures of historic climatic events in an Antarctic octopus. *Mol. Ecol.* **21**, 2775-2787 (2012).

73. A.K. Wåhlin et al., Some implications of Ekman layer dynamics for cross-shelf exchange in the Amundsen Sea. *J. Phys. Oceanogr.* **42**, 1461-1474 (2012); https://doi.org/10.1175/JPO-D-11-041.1.

74. A. Dutton, R.M. DeConto, Genetic insight on ice sheet history. *Science* **382**, 1356-1357 (2023); https://doi.org/10.1126/science.adm6957.

75. R. Petschick, G. Kuhn, F.X. Gingele, Clay mineral distribution in surface sediments of the South Atlantic: Sources, transport, and relation to oceanography. *Mar. Geol.* **130**, 203-229 (1996).

76. S. Aoki, K. Oinuma, Clay mineral composition in shallow water sediment samples near the Antarctic Peninsula and in deep-sea core samples from the Pacific and the Indian-Antarctic basins. *J. Oceanogr.* **36**, 23-29 (1980).

77. C.-D. Hillenbrand et al., Clay mineral provenance of sediments in the southern Bellingshausen Sea reveals drainage changes of the West Antarctic Ice Sheet during the Late Quaternary. *Mar. Geol.* **265**, 1-18 (2009).

78. W. Ehrmann et al., Provenance changes between recent and glacial-time sediments in the Amundsen Sea embayment, West Antarctica: clay mineral assemblage evidence. *Antarct. Sci.* **23**, 471-486 (2011).

79. J.A. Smith et al., Deglacial history of the West Antarctic Ice Sheet in the western Amundsen Sea Embayment. *J. Quat. Sci. Rev.* **30**, 488-505 (2011).

80. J.A. Smith et al., New constraints on the timing of West Antarctic Ice Sheet retreat in the eastern Amundsen Sea since the Last Glacial Maximum. *Global Planet. Change* **122**, 224-237 (2014).

81. A.G.C. Graham, J.A. Smith, Palaeoglaciology of the Alexander Island ice cap, western Antarctic Peninsula, reconstructed from marine geophysical and core data. *J. Quat. Sci. Rev.* **35**, 63-81 (2012).

82. W. Dickens, *Reconstructing glacial and environmental change on the South Orkney Plateau, sub-Antarctica*. (PhD thesis, Department of Geography, University of Cambridge, Cambridge, UK, 2016), 225 pp.; https://www.dropbox.com/s/resrqzu7jjuq7bf/Dickens%20PhD%20Thesis%202016_Revised%2013th%20May.pdf?dl=0.

83. Z.A. Roseby et al., History of Anvers-Hugo Trough, western Antarctic Peninsula shelf, since the Last Glacial Maximum. Part I: Deglacial history based on new sedimentological and chronological data. *J. Quat. Sci. Rev.* **291**, 107590 (2022); https://doi.org/10.1016/j.quascirev.2022.107590.

84. J.A. Smith et al., “Using sub-ice-shelf sediments to reconstruct glacial history” in *24th Annual West Antarctic Ice Sheet (WAIS) Initiative Workshop 2017, 8-11 October 2017, Camp Casey Conference Center, Coupeville, Washington (U.S.A.*) (2017a); https://www.waisworkshop.org/sites/waisworkshop.org/files/webform/2017/abstracts/Smith_J_ii_2017-abstract.pdf.

85. J.A. Smith et al., History of the Larsen C Ice Shelf reconstructed from sub-ice shelf and offshore sediments. *Geology* **49**, 978-982 (2021).

86. P. Simões Pereira et al., Geochemical fingerprints of glacially eroded bedrock from West Antarctica: Detrital thermochronology, radiogenic isotope systematics and trace element geochemistry in Late Holocene glacial-marine sediments. *Earth Sci. Rev.* **182**, 204-232 (2018).

87. J.A. Smith et al., Sub-ice-shelf sediments record history of twentieth-century retreat of Pine Island Glacier. *Nature* **541**, 77-80 (2017b).

88. P. Simões Pereira et al., The geochemical and mineralogical fingerprint of West Antarctica’s weak underbelly: Pine Island and Thwaites glaciers. *Chem. Geol.* **550**, 119649 (2020).

89. C.-D. Hillenbrand, H. Grobe, B. Diekmann, G. Kuhn, D.K. Fütterer, Distribution of clay minerals and proxies for productivity in surface sediments of the Bellingshausen and Amundsen seas (West Antarctica) – Relation to modern environmental conditions. *Mar. Geol.* **193**, 253-271 (2003).

90. J.P. Klages et al., Retreat of the West Antarctic Ice Sheet from the western Amundsen Sea shelf at a pre- or early LGM stage. *J. Quat. Sci. Rev.* **91**, 1-15 (2014).

91. S. Wu et al., Surface sediment characteristics related to provenance and ocean circulation in the Drake Passage sector of the Southern Ocean. *Deep-Sea Res. I* **154**, 103135 (2019).

92. C.-D. Hillenbrand, W. Ehrmann, J.A. Smith, “Clay mineral assemblages in Antarctic continental margin sediments: Successful applications and pitfalls in reconstructing palaeoenvironments from detrital provenance indicators” in *11th International Symposium on Antarctic Earth Sciences (ISAES), 10-15 July 2011, Edinburgh (UK), Programme and Abstracts*, M. Siegert et al., Eds. (Scientific Committee for Antarctic Research, 2011), p. 215.

93. J.W. Marschalek et al., Byrd Ice Core debris constrains the sediment provenance signature of central West Antarctica. *Geophys. Res. Lett.* **51**, e2023GL106958 (2024); https://doi.org/10.1029/2023GL106958.

94. K. Matsuoka et al., Quantarctica, an integrated mapping environment for Antarctica, the Southern Ocean, and sub-Antarctic islands. Environ. Model. Softw. 140, 105015 (2021); https://doi.org/10.1016/j.envsoft.2021.105015.

95. J.A. Arndt et al., The International Bathymetric Chart of the Southern Ocean (IBCSO) Version 1.0 — A new bathymetric compilation covering circum-Antarctic waters. *Geophys. Res. Lett.* **40**, 3111-3117 (2013); https://doi.org/10.1002/grl.50413.

96. Scientific Committee on Antarctic Research (SCAR), Antarctic Digital Database (ADD) Version 7.0 [Data set]. British Antarctic Survey, Cambridge, UK (2016); https://www.add.scar.org/ [accessed 2022].

97. C. Cucciniello, Tetra-Plot: A Microsoft Excel spreadsheet to perform tetrahedral diagrams. *Periodico di Mineralogia* **85**, 115-119 (2016).

98. L.E. Lisiecki, M.E. Raymo, Pliocene-Pleistocene stack of 57 globally distributed benthic $\delta^{18}O$ records. *Paleoceanography* **20**, PA1003 (2005); https://doi.org/10.1029/2004PA0010712005.

99. B. Hopkins et al., Evaluation of geomagnetic relative palaeointensity as a chronostratigraphic tool in the Southern Ocean: Refined Plio-/Pleistocene chronology of IODP Site U1533 (Amundsen Sea, West Antarctica). *J. Quat. Sci. Rev.* **325**, 108460 (2024); https://doi.org/10.1016/j.quascirev.2023.108460.

100. Esquevin, J. Influence de la composition cimique des illites sur le cristallinite. *Bull. Cent. Rech. Pau. S.N.P.A.* **3**, 147-154 (1969).

101. Y.K. Park et al., Elemental composition of smectite minerals in continental rise sediments from the Amundsen Sea, West Antarctica, as a tool to identify detrital input from various sources throughout late Quaternary glacial-interglacial cycles. *Chem. Geol.* **657**, 122116 (2024); https://doi.org/10.1016/j.chemgeo.2024.122116.

102. M. Kloster, G. Kauer, O. Esper, N. Fuchs, B. Beszteri, Morphometry of the diatom *Fragilariopsis kerguelensis* from Southern Ocean sediment: High-throughput measurements show second morphotype occurring during glacials. *Mar. Micropal.* **143**, 70-79 (2018); https://doi.org/10.1016/j.marmicro.2018.07.002.

103. J.A. Ruggiero et al., Population morphometrics of the Southern Ocean diatom *Fragilariopsis kerguelensis* related to sea surface temperature. *J. Micropal.* **43**, 323-336 (2024); https://doi.org/10.5194/jm-43-323-2024.

104. J.R. Mastro, *Estimating paleotemperatures in the Amundsen Sea for the last 620,000 years through diatom morphometrics (IODP Expedition 379)*, Graduate Research Theses & Dissertations **7410**, 43 pp. (Northern Illinois University, DeKalb, U.S.A., 2020); https://huskiecommons.lib.niu.edu/allgraduate-thesesdissertations/7410.

105. R.A. Locarnini et al., *World Ocean Atlas 2013, Volume 1: Temperature*, S. Levitus (Ed.), A. Mishonov (Technical Ed.); *NOAA Atlas NESDIS* **73**, 40 pp. (National Oceanographic Data Center, Silver Spring, Maryland, U.S.A., 2013); https://doi.org/10.7289/V55X26VD.
