# Supplementary material for: Repeated loss of the West Antarctic Ice Sheet’s Amundsen Sea sector during late Pleistocene interglacials
Source: Nat Commun. 2026 Nov 9;17:10450. doi: 10.1038/s41467-026-77004-2 (PMC13748572; doi:10.1038/s41467-026-77004-2)
Supplement: Supplementary file 2 [file 41467_2026_77004_MOESM2_ESM.pdf]

Description of Additional Supplementary Files

Supplementary Data Captions S1-S5

Reference numbers refer to the reference list provided in the Supplementary Information.

Supplementary Data S1: Age models and linear sedimentation rates for the late Pleistocene–Holocene sections of IODP Exp. 379 sites U1532 and U1533[5] and piston core PC494. For Site U1533, the Brunhes–Matuyama chron boundary (B/M) refined by Hopkins et al.[99] was used. For core PC494, an age model based on lithostratigraphy only had been developed (age-depth fix points in normal font) that was then refined by geomagnetic relative palaeointensity (RPI) measurements[6] (age-depth fix points in italics). MIS: Marine Isotope Stage; CSF-A: core depth below seafloor method A[5]; CCSF-M: core composite depth below seafloor method M[5]; mbsf: core depth in metres below seafloor; Lithostrat.: Lithostratigraphy; LR04: LR04 benthic foraminifera $\delta^{18}O$ stack[98]; LSR: Linear Sedimentation Rate; *: LSRs of age models not distinguishing between MIS-2, -3 and -4 (U1532, U1533) and between MIS-5a-d and MIS-5e (U1533), or based on lithostratigraphy refined by RPI correlations (PC494).

Supplementary Data S2: Contents of the clay minerals smectite, illite, chlorite and kaolinite in the <2 µm fraction of contemporary (i.e., latest MIS-1 [14–0 ka] to modern) seafloor-surface sediments from the eastern Pacific and SW Atlantic sectors of the Antarctic continental margin and basal glacigenic debris of the Byrd Ice Core (BIC). Near-coastal shelf and sub-Antarctic SE Pacific Basin samples characterising the source sectors and sub-sectors listed in Table 1 of the main paper and plotted in Fig. S2 as well as references for clay mineral data and sample

information are also given. Depths of samples marked with an asterisk (samples from BIC) are depths below the ice-sheet surface.

Supplementary Data S3: Contents of the clay minerals smectite, illite, chlorite and kaolinite in the <2 µm fraction of late Pleistocene sediments at IODP Exp. 379 sites U1532 and U1533[5] and piston core site PC494[6]. For illite, the 5Å/10Å peak area ratio (= Esquevin Index[100]), which is a proxy for its chemical composition, is also given. CSF-A: core depth below seafloor method A[5]; CCSF-M: core composite depth below seafloor method M[5]; cmbsf: core depth in centimetres below seafloor.

Supplementary Data S4: Relative contents of aluminium (Al^{VI}), magnesium (Mg^{VI}) and iron (Fe^{3+}) in smectite minerals of samples from contemporary seafloor-surface sediments[101] and late Pleistocene glacial and interglacial sediments at IODP Exp. 379 sites U1532 and U1533 and piston core site PC494 (contents normalised to 100%). cmbsf: core depth in centimetres below seafloor; CSF-A: core depth below seafloor method A[5]; CCSF-M: core composite depth below seafloor method M[5].

Supplementary Data S5: Summer sea-surface temperatures (SSSTs) for each of the interglacial Marine Isotope Stages (MIS) from MIS-5 (130–71 ka) back to MIS-15 (621–563 ka) including glacial MIS-14 (563–533 ka), which had characteristics of an interglacial period[55], at Site U1533. All SSSTs were calculated from morphological variations of frustules of the diatom species *Fragilariopsis kerguelensis* using the calibration of Kloster et al.[102], and numbers of morphometric measurements per MIS are also given. SSST for MIS-5 is from Ruggiero et al.[103], while all other SSSTs are from Mastro[104]. Modern SSST in the vicinity of Site U1533 is -0.51 to +0.34 °C[103,105], i.e., the average SSST of +0.9 °C for MIS-7 (243–191 ka) implies a warming by ca. 0.6 to 1.4 °C compared to today.
