# Supplementary material for: Repeated loss of the West Antarctic Ice Sheet’s Amundsen Sea sector during late Pleistocene interglacials
Source: Nat Commun. 2026 Nov 9;17:10450. doi: 10.1038/s41467-026-77004-2 (PMC13748572; doi:10.1038/s41467-026-77004-2)
Supplement: Supplementary file 3 [file 41467_2026_77004_MOESM8_ESM.pdf]

nature portfolio

Peer Review File

Repeated loss of the West Antarctic Ice Sheet's Amundsen Sea sector during late Pleistocene interglacials

Corresponding Author: Dr Claus-Dieter Hillenbrand

This file contains all reviewer reports in order by version, followed by all author rebuttals in order by version
Parts of this Peer Review File have been redacted as indicated to remove third-party material.

Version 0:

Reviewer comments:

Reviewer #1

(Remarks to the Author)
In this study, the authors analyse the modern distribution of clay minerals in surface samples from the West Antarctic Ice Sheet (WAIS) sector to reconstruct past sediment provenance. Their results indicate that material derived from glaciers draining the WAIS into the Amundsen Sea sector was greatly reduced, or nearly absent, during interglacial periods. These findings suggest that the WAIS experienced substantial mass loss during these intervals.

The inferred WAIS deglaciation appears to have been driven primarily by ocean forcing and contributed to global sea-level rise. Importantly, the results indicate that such WAIS retreat may be triggered at temperature increases of only ~1–1.5 °C above pre-industrial levels—comparable to conditions during MIS 5e—highlighting the potential for the WAIS to approach a tipping point under near-future warming scenarios.

I found the applied methods to be sound and well described. The approach of using modern clay mineral distributions to reconstruct past sediment delivery and infer ice-sheet evolution is clearly presented and conceptually straightforward. To further strengthen the study, the authors could consider providing additional detail on the SSST reconstructions derived from diatom valve morphology.

General suggestions:
1) One site is associated with multiple core codes. I recommend that the authors list all relevant codes (PS2547, PC493, PS2551, PS58/254, PC494) at the first mention of the core and then specify a single code to be used consistently thereafter for simplicity (e.g., "hereafter referred to as PC494"). The core code used in the manuscript should also be used in the figures to ensure clarity.

2) More generally, I think there is an issue with interpretations and hypotheses being introduced and discussed within the Results section. The Results should remain primarily descriptive. I have tried to highlight specific instances where I recommend either moving the interpretative content to the Discussion or, alternatively, considering a combined Results and Discussion section.

Abstract:
I suggest not using abbreviations if possible.

Introduction:
L.109-112: This is a very long sentence, and I suggest breaking it up for clarity. In particular, the statement on MIS 5e would benefit from slight expansion. For example: "Far-field records of past global sea-level highstands and coupled ice-sheet climate model results5, suggest that partial or even total WAIS "collapse", i.e., major, rapid retreat, occurred during some warm periods of the Brunhes chron (773–0 ka). This includes the last peak interglacial of Marine Isotope Stage (MIS) 5e (130–115 ka), which is regarded as a key analogue for a near future scenario with temperatures ~1–1.5 °C above pre-industrial levels."

L. 124: I suggest expanding slightly on the type of seawater temperature proxy introduced here.

Results

L. 199-201: Instead of stating that “down-core Ba/Rb and Br/Rb records show distinct maxima during interglacial intervals,” the authors interpret these maxima as reflecting increased phytoplankton productivity in surface waters. I would argue that this interpretative component belongs in the Discussion rather than the Results, as it moves beyond description into proxy interpretation.

L206–209: This section is not purely descriptive but includes interpretative elements, which would be more appropriate for the Discussion.

L204–209: The phrase “…probably by interactions of bottom currents with the fine-grained suspension load…” represents an interpretation of depositional processes. I recommend moving this to the Discussion section.

L211–216: This is a very long sentence and is difficult to follow. I suggest breaking it up for clarity.

L221: Again, this sentence is quite long and could be separated for readability. For example: “…delivery of iceberg-rafted debris (IRD). The IRD was likely deposited when WAIS sectors …”

L293: The phrasing “…support the hypothesis that …” is interpretative and would be better placed in the Discussion rather than the Results.
Discussion

L349: When reading this section, I wondered why the contemporary samples from Core U1533 were not included in the plot. Did these samples also exhibit Type-2 smectite? If so, their inclusion could further support these interpretations.

Methods:
L420: Please add a reference to Figure 1.

L468: This is a clear and helpful explanation. You may wish to quantify the temporal offset (ca. 13,000 years) to give readers a better sense of the impact of lock-in depth on the chronology.

L469: Although this is mentioned earlier, I suggest beginning with a clarifying sentence noting that this record was not analysed as part of the present study but is instead used for comparison.

L489–490: “…Sites U1533 and U1532 were measured at 2-cm depth resolution during IODP Exp. 379 using a whole-round…” I suggest deleting “on board”.

L508–537: This paragraph describes the collection of surface samples. I recommend introducing a separate section (e.g., “Contemporary Surface Sediments”) following “Sediment Cores and Age Model,” which would make this information easier for readers to locate.

L537: This is a useful point. You could consider adding that gravity cores (and similar surface cores) may be subject to over-penetration; therefore, without surface dating (e.g., ^{210}Pb), it is not always possible to confirm that the recovered sediment is truly modern.

L600: “…of the diatom species Fragilariopsis kerguelensis (F. kerguelensis)[66–68]…”

Figures:
Figure 1: “Amundsen Sea” is already spelled out in the figure itself and therefore does not need to be repeated in the caption. I suggest removing it from the figure text. In addition, please define the abbreviations WAIS and APIS in the caption for clarity.

Reviewer #2

(Remarks to the Author)
Hillenbrand et al. provide a manuscript regarding WAIS deglaciation episodes in the last 800 kiloyears, starting from MIS 19, and discuss how these events can be used to infer information about WAIS current situation and its future projections. To do so, the Authors analyze clay mineral assemblages from various sources contained in the stratigraphic sequence, coupled with a diatom-derived paleotemperature proxy and with XRF elemental ratios. All proxies point towards a systematic deglaciation and ungrounding, for all the interglacial periods before the Holocene, of the Amundsen sector of the WAIS, in conjunction with an increase in sea surface temperature evidenced by the diatom-derived proxy. The Authors conclude that the deglaciation and ice loss during interglacial periods was determined primarily by ocean forcing, with increased advection of warm CDW towards the Amundsen shelf, and that this process, coupled with ongoing anthropogenic emissions, will inevitably cause an amplified rise of the sea level.
The manuscript is well researched, well written and the data are presented in an overall exhaustive way. The methodology is sound and the entire data set used by the authors is available and easy to read. The results are solid and innovative, being extremely important to understand the evolution of the Antarctic ice sheet during the Pleistocene, especially for the

community revolving around paleo-reconstruction in polar environments and for modelling future projections. My comments concern some minor modifications, mostly to enhance data visualization and to clarify certain aspects of the work, apart from a couple of secondary aspects that, in my opinion, deserve to be discussed. The manuscript has already a very valid structure and I think that, after these few adjustments, it will be ready for publication.

GENERAL COMMENTS
1) The Authors state that the low smectite content of glacial sediments from both IODP cores can be related to a weakened poleward transport by CDW advection. I agree with the interpretation, but I would point out that inferring this from the smectite content alone could be misleading, since the content could be lower even in a scenario of increased transport of smectite by the CDW, if the sedimentation rate contemporarily increases and the mineral gets diluted in a much larger volume. From Figs. 3 and 4 it seems that the sedimentation rate generally decreases during glacial periods, thus corroborating the authors' interpretation, but I would like this point to be, even if briefly, discussed in the text for clarity. In Table S1 the Linear Sedimentation Rate is reported (actually, there are two different LSR for both cores, without a clear distinction in the column header), but it is not clear from the layout of the table which sedimentation rates are associated with glacials and which ones with interglacials. Would it be possible to plot, maybe in the SI (in Fig. S3 or in a separate panel), the sedimentation rates estimated from the age model?

2) The paleo-SSST reconstruction is compelling, but one thing that is not discussed is the apparent tendency of having higher temperatures going downcore, which is, at best, mildly suspicious. I don't seem to find any mention of this trend in the manuscript, in the SI or in the cited references for the methodological approach (42 and 69). I am also curious about SSST reconstruction results from the glacial periods: are they possible or the poor diatom content makes it unfeasible? Also, is there any way to have SSSTs for different intervals inside the same interglacial? I would expect temperatures to be higher (and thus higher CDW advection) at the end of the glacial/at the onset of the interglacial (see for example the recent work from Pambianco et al., 2025 (Ref. 1)), and it would be interesting to compare these temperatures with other independent SST reconstructions to see if they match.

3) The clay mineral assemblages are the core of the work, but it is sometimes difficult for the reader to follow all the sources and associations for the different minerals and elemental compositions. I suggest implementing a brief explanation/list, maybe in a table, to sum up these details so that one can use it as a reference during the reading.

SPECIFIC COMMENTS
Lines 97-98: "This melting increases discharge of grounded ice feeding into and buttressed by the ice shelves". Please expand on this concept, the sentence is not very clear as it is.
Lines 103-108: I would rephrase as: Here, potentially irreversible ice loss from two major outlets draining into the eastern Amundsen Sea Embayment (Thwaites and Pine Island glaciers, Fig. 1) may initiate not only widespread deglaciation in this sector but also in other marine-based WAIS sectors and in the entire WAIS within the coming centuries, raising global sea-level by 3.3–5.3 metres
Line 112: Change "last peak interglacial" with "last interglacial peak"
Line 134: "sediments from its margin". Add "continental" margin.
Lines 195-196: References are missing for these ratios (they are present in the method section, but they should be cited here as well). Also, why Br was not normalized with Cl (Ref. 2)?
Lines 211-216: This sentence is very long. I suggest dividing it in two parts, for example: "This disparity most likely results from the dissimilar locations of the two sites on Resolution Drift. The relative contributions of different sediment transport processes affecting drift deposition throughout late Pleistocene glacial-interglacial cycles are known to vary systematically across Antarctic rise drifts, creating distinct, localized depositional settings."
Lines 251-255: Very long sentence. I suggest dividing it in two parts, for example: "The prominent kaolinite reduction in the interglacial sediments on the eastern Amundsen Sea rise does not solely result from reduced deposition of kaolinite enriched glacigenic detritus from the Amundsen Sea shelf. In this case, a corresponding prominent increase of both illite and chlorite is to be expected but is not observed". Besides, this first sentence introduces kaolinite, and then the focus of the paragraph shifts abruptly towards smectite. I would move this part at the end of the paragraph (line 277), after the explanation regarding the high kaolinite concentrations during glacial times.
Line 260: PC494 is missing from the map. If I understood this correctly, it should be located on the same site as BC495, but this is not clarified in the figure's caption. I would nonetheless suggest inserting the name of the core on the map.
Line 265: Move "exclusively" after "to contain".
Line 294: Rephrase "smectites in the interglacial rise sediments" as "smectites in the Amundsen Sea rise interglacial sediments" or just "smectites in the interglacial sediments".
Lines 328-334: Very long sentence. I suggest dividing it, for example "At Site U1533 the Esquevin index of illite in the pre-Holocene interglacial sediments mainly ranges from 0.30–0.40 (Fig. 3), revealing that this illite – together with other detritus – was predominantly derived from the Thurston Island block rather than Marie Byrd Land. There, the Esquevin index is typically ≤0.3029 333, and, thus, corroborates continued glacial erosion in the Thurston Island block area"
Lines 340-341: This assumption is a bit stretched. It is true that site PS2551-1 is further west from the IODP sites, but a transport from eastern Marie Byrd Land dome towards NW/WNW would miss site PS2551-1, which is located almost straight up north to Marie Byrd Land.
Lines 397-399: Please further elaborate on this concept or refer to the explanation in the SI.
Lines 406-411: This sentence is a bit convoluted, I would suggest dividing it in two sentences or to shorten it to make it clearer.
Line 420: Remove the initial "while" and close the sentence at row 423 after "90%" with a dot.
Line 534: Add a comma after "sediments".
Line 548: Change "they were" with "being".

Line 908: Amundsen Sea is already spelled out on the map. WAIS and APIS are missing from the caption.
Line 950: The EI is not plotted on the Zr/Rb ratio but on the Ti/K ratio.
Line 973: See previous comment.

FIGURES
The figures are overall quite clear and understandable, but I would suggest making some improvements in order to enhance the representation of the dataset.

Figure 1
- Names of the seas and geographic indications: the sea names are aligned and justified in different ways. I think this partially impedes the readability of the figure. In order to make the figure tidier and easier to read, I suggest arranging the names in a more symmetrical way. The Patagonia name is divided, it would be tidier to leave it on one row, if possible.
- Core names and locations: core names and symbols are not well readable, they are significantly smaller than geographic locations and often black on a dark blue background. I would suggest changing the colors (e.g. from black to white) and/or add an outline. You could also add a third panel with a focus on the Amundsen Sea sector and embayment, where most of the cores are located, showing the cores and the main locations (e.g. Amundsen Rise, etc), without the surface sediments if this helps readability. This panel could be also useful to graphically show, with arrows, the preferential transport paths described in the main text, which would help the reader following the main discussion.
- Symbology: you use different symbols and colors corresponding to different % of minerals. The brown triangles are often not well distinguishable on the blue background. You may use the same symbol for all the categories, with symbol size proportional to the % (e.g. small star for low %, big star for high %, in-between star size for in-between %). Even better, it would be ideal to couple this graphic representation with a color palette (e.g. reddish-white for low %, red for high %, in-between shades for in-between %), ideally choosing a different color palette for each mineral.

Figure 2
It is challenging to read this diagram in its entirety. Firstly, I would suggest moving type1 and type2 arrows on the right axis (Mg) and separating the legend, listing the glacial samples under panel 2b. The colours for BC27, BC480 and BC502 contour lines are kind of similar, and it is not easy to distinguish them. Also, looking at the relatively consistent compositions of contemporary samples (excluding sub-Antarctic SE Pacific Basin samples), it could be more reader-friendly to plot an "average" contour line from all sources of Type 1 smectites.

Figures 3 and 4
The figures are simple yet clear. It would have been nice to report the core photos somewhere, i.e. in the supplementary material.

References
1. Chiara Pambianco et al., Past intrusion of Circumpolar Deep Water in the Ross Sea: Impacts on the ancient Ross Ice Shelf. Sci. Adv. 11, eadt7075(2025). DOI: 10.1126/sciadv.adt7075
2. Croudace, I.W., Rothwell, R.G. (Eds.), 2015. Micro-XRF Studies of Sediment Cores: Applications of a non-destructive tool for the environmental sciences, Developments in Paleoenvironmental Research. Springer Netherlands, Dordrecht. https://doi.org/10.1007/978-94-017-9849-5

Reviewer #3

(Remarks to the Author)
I co-reviewed this manuscript with one of the reviewers who provided the listed reports. This is part of the Nature Communications initiative to facilitate training in peer review and to provide appropriate recognition for Early Career Researchers who co-review manuscripts.

Reviewer #4

(Remarks to the Author)
The manuscript of Hillenbrand and co-authors presents a robust and multidisciplinary dataset (XRF, clay mineralogy, magnetic susceptibility, and bulk density) aimed at reconstructing the dynamics of West Antarctic Ice Sheet (WAIS) retreat during the glacial-interglacial period of the last 800 kyr. The dataset is solid and carefully constructed, and I commend the authors for compiling and presenting it. In addition, the integration with modern datasets is particularly strong and significantly enhances the overall impact of the study.
The multiproxy approach convincingly highlights clear differences between glacial and interglacial patterns, and the link to oceanographic processes is well developed and compelling. The scientific concept is strong: the manuscript not only reconstructs past changes but also provides meaningful implications for future WAIS behaviour under ongoing climate warming. Overall, these aspects make the study suitable for publication in a journal such as Nature Communications. I particularly appreciated the Discussion section, especially the connection drawn between past dynamics and future scenarios. I thank the authors for this contribution, which I believe will be valuable to the scientific community.
However, some aspects of the manuscript should be improved before acceptance.
The main issue observed throughout both the Introduction and the Results sections, concerns writing style and structure. Many sentences are very long and occasionally difficult to follow. This is not a problem of scientific content, which is strong, but rather of how the results are communicated. I encourage the authors to revise the text for clarity and conciseness.
In addition, the distinction between Results and Discussion is sometimes confused. While some degree of overlap is

understandable in this type of article, parts of the Results section appear overly interpretative. I recommend reducing the level of interpretation in the Results and reserving more speculative or integrative statements for the Discussion.
These comments are intended as constructive suggestions to improve clarity and readability. Importantly, they do not detract from the overall quality and significance of the work, which I consider to be high.
For these reasons, I recommend minor revision.
Specific comment:
Introduction
The introduction provides a clear and relevant overview of the vulnerability of the West Antarctic Ice Sheet (WAIS) and effectively frames the importance of understanding its response to past and future climate change. The authors successfully highlight the role of ocean forcing, particularly Circumpolar Deep Water (CDW), and place the study within the broader context of sea-level rise and interglacial variability. The scientific motivation is therefore well established.
However, several aspects of the Introduction could be improved to enhance clarity and readability. The main issue concerns sentence structure: many sentences are overly long and contain multiple layers of information (e.g., lines 95-97;109-113), which makes the text at times difficult to follow. I recommend revising these passages to improve conciseness and flow, for example by splitting complex sentences and simplifying phrasing where possible.
In addition, the transition from the general background to the specific knowledge gap could be made sharper (See lines 109 -118). While the Introduction discusses uncertainties in past WAIS behaviour, the exact gap that this study aims to address is not always stated as explicitly as it could be. Strengthening this point would help to better position the novelty and relevance of the work.
Finally, the concluding paragraph of the Introduction could be made more direct and impactful. The current formulation is somewhat descriptive (e.g., “Here we investigate…”), whereas a more assertive statement of the study’s objectives and approach would improve the overall framing.
Overall, the Introduction is scientifically sound and well contextualized, but would benefit from stylistic refinement and a clearer articulation of the research gap.
Results
Clay mineral provenance of Antarctic marine sediment: Fingerprinting source areas
The first part of the Results provides important information for the subsequent interpretations. The compilation of modern and previously published datasets is particularly valuable, and the identification of kaolinite as a key tracer for the Amundsen Sea sector is well motivated. The integration of mineralogical and geochemical data represents a clear strength of the study.
However, this section would benefit from several structural and stylistic improvements. A substantial portion of the text is devoted to general background on Antarctic weathering processes and clay mineral formation, which partly repeats information that is already expected to be known by the readership or would be better suited to the Introduction. I recommend reducing this descriptive component and focusing more directly on the novel results of the study. For example, in my opinion, lines 131–143 could be removed or significantly shortened and incorporated into the Introduction.
As in the Introduction, several sentences are too long and contain different concepts, which makes the text difficult to follow. I suggest rephrasing sentence structure and improving conciseness would greatly enhance readability.
Overall, this section is robust and clearly relevant, but it would benefit from a clearer distinction between background and new results, improved readability, and a more balanced presentation of interpretations.
Major glacial-interglacial changes in WAIS extent are archived in clay mineral provenance data from the Amundsen Sea continental rise
This section presents a detailed and well-supported description of glacial–interglacial variability in sedimentary facies, physical properties, and clay mineral assemblages. The multiproxy dataset provides a coherent picture of environmental change across glacial–interglacial cycles.
In particular, the identification of variability between bioturbated diatom-rich intervals and laminated terrigenous units, as well as their linkage to interglacial and glacial conditions, is convincing and in agreement with previous studies from the Amundsen Sea margin. The integration of geochemical proxies (e.g., XRF ratios) further strengthens the interpretation of productivity changes and sedimentary environments.
Remains unclear the interpretations the turbidity current (lines 206-211) are presented with a relatively high degree of confidence, despite being indirectly inferred. While these interpretations are plausible, they should be expressed more cautiously unless supported by additional independent evidence.
Finally, as in other parts of the manuscript, several sentences are overly long and combine multiple concepts, which reduces readability. Simplifying sentence structure and separating observational results from interpretative statements would significantly improve clarity.
Discussion:
I found the Discussion to be of high quality and very engaging. I particularly appreciated the final “Global implications” section, which significantly strengthens the impact of the manuscript. Thank you to the authors for this thoughtful addition.
The glacial–interglacial variability is well supported by the multiproxy approach and provides a coherent picture of environmental changes across glacial–interglacial cycles.
I have one point that I found unclear. In lines 314–317, the authors attribute the observed changes in clay mineral assemblages primarily to a cessation of subglacial erosion. However, it is not fully clear why alternative mechanisms are not considered or discussed. Could dilution effects or changes in sediment supply and transport pathways also contribute to the observed signal? A brief discussion of potential alternatives would strengthen the interpretation's robustness.
Figure
The figures are well prepared, clear, and of high quality, and I have no major comments on them.
My only observation, which I understand the authors may choose not to address, concerns the lack of a detailed bathymetric map or geophysical image showing the precise location of the cores. While not essential, including such a figure could further improve the manuscript and better contextualize the study area.
Material and methods
Are clear and support the data.

Supplementary materials are clear and supportive of the draft and not excessively long.

Version 1:

Reviewer comments:

Reviewer #1

(Remarks to the Author)
The authors have addressed all the comments and suggestions, and I find the manuscript to be ready for submission. I have no further comments.

Reviewer #2

(Remarks to the Author)
The Authors adequately addressed all requests and scientific questions, providing an exhaustive response. As for me, The paper is now ready for publication.

Reviewer #3

(Remarks to the Author)
I co-reviewed this manuscript with one of the reviewers who provided the listed reports. This is part of the Nature Communications initiative to facilitate training in peer review and to provide appropriate recognition for Early Career Researchers who co-review manuscripts.

Open Access This Peer Review File is licensed under a Creative Commons Attribution 4.0 International License, which permits use, sharing, adaptation, distribution and reproduction in any medium or format, as long as you give appropriate credit to the original author(s) and the source, provide a link to the Creative Commons license, and indicate if changes were made.
In cases where reviewers are anonymous, credit should be given to 'Anonymous Referee' and the source.
The images or other third party material in this Peer Review File are included in the article's Creative Commons license, unless indicated otherwise in a credit line to the material. If material is not included in the article's Creative Commons license and your intended use is not permitted by statutory regulation or exceeds the permitted use, you will need to obtain permission directly from the copyright holder.
To view a copy of this license, visit https://creativecommons.org/licenses/by/4.0/

Response to reviewer comments on manuscript NCOMMS-26-010571 “Repeated loss of the West Antarctic Ice Sheet’s Amundsen Sea sector during late Pleistocene interglacials“ submitted by C.-D. Hillenbrand et al. to *Nature Communications*

We thank all four referees for their thoughtful and constructive comments. In the following we list their comments (in bold font) and give our responses (in italics). All line numbers given in our response refer to those in the revised manuscript version without track changes.

Reviewer #1 (Remarks to the Author):

In this study, the authors analyse the modern distribution of clay minerals in surface samples from the West Antarctic Ice Sheet (WAIS) sector to reconstruct past sediment provenance. Their results indicate that material derived from glaciers draining the WAIS into the Amundsen Sea sector was greatly reduced, or nearly absent, during interglacial periods. These findings suggest that the WAIS experienced substantial mass loss during these intervals.

The inferred WAIS deglaciation appears to have been driven primarily by ocean forcing and contributed to global sea-level rise. Importantly, the results indicate that such WAIS retreat may be triggered at temperature increases of only ~1–1.5 °C above pre-industrial levels—comparable to conditions during MIS 5e—highlighting the potential for the WAIS to approach a tipping point under near-future warming scenarios.

I found the applied methods to be sound and well described. The approach of using modern clay mineral distributions to reconstruct past sediment delivery and infer ice-sheet evolution is clearly presented and conceptually straightforward. To further strengthen the study, the authors could consider providing additional detail on the SSST reconstructions derived from diatom valve morphology.

We thank Reviewer#1 for the overall positive evaluation of our study and address questions regarding the SSST reconstruction in our response to a related comment by Reviewer#2.

General suggestions:

1) One site is associated with multiple core codes. I recommend that the authors list all relevant codes (PS2547, PC493, PS2551, PS58/254, PC494) at the first mention of the core and then specify a single code to be used consistently thereafter for simplicity (e.g., “hereafter referred to as PC494”). The core code used in the manuscript should also be used in the figures to ensure clarity.

Done for PS2547 & PC493 and BC495 & PC494, which are pairs of cores recovered from two separate locations (core IDs in Figs. 1, 2 and S1 were changed accordingly). All other cores are from different locations, and we have slightly rearranged core ID labels in the maps of Figs. 1 and S1 to clarify this.

2) More generally, I think there is an issue with interpretations and hypotheses being introduced and discussed within the Results section. The Results should remain primarily descriptive. I have tried to highlight specific instances where I recommend either moving the interpretative content to the Discussion or, alternatively, considering a combined Results and Discussion section.

We are grateful to Reviewer#1 for pointing this out. We are convinced that a strict separation of the “Results” and their ”Interpretation” would lead to too much text repetition. This is because we would have to briefly report the results again when interpreting them in a separate “Interpretation” section. On the other hand, we fear that combining the

interpretation of the results with the "Discussion" section, which focusses on past WAIS variations and their forcing, the "Discussion" would lose its coherence. We emphasise that our interpretations of the analysed proxies (e.g., element ratios Si/Al and Ba/Rb as productivity proxies, Zr/Rb as grain-size proxy, etc.) are generally accepted in the literature, while the interpretation of the depositional processes at sites U1533 and U1532 is based on previously published literature on the IODP Exp. 379 sites as well as conventional sediment cores and seismic profiles from the Amundsen Sea continental rise (see references in the main paper and the SI). Therefore, we have chosen to follow the reviewer's 2nd suggestion and combined the presentation of the results with their interpretation. We have renamed this section "Results and Interpretation", which (i) clarifies for the reader that results are interpreted in this section but (ii) still allows us to focus the "Discussion" section on the main implications of our findings for ice-sheet history. Furthermore, we clarify now that our interpretations of the XRF-derived palaeo-productivity proxies are based on previously published literature and refer to the study by Hopkins et al. (2024) on the Ba/Rb record from Site U1533 already in this section (in the original submission we had referred to this paper only in relation to the magnetostratigraphy; thanks for making us aware of this omission). We also added a reference to Konfirst et al. (2012), who had presented and discussed diatom abundance and species data for nearby site PS58/254-1 in addition to biogenic barium concentrations, biogenic opal contents and density data (earlier published by Hillenbrand et al. 2009).

Abstract:

I suggest not using abbreviations if possible.

We removed all abbreviations from the abstract as recommended.

Introduction:

L.109-112: This is a very long sentence, and I suggest breaking it up for clarity. In particular, the statement on MIS 5e would benefit from slight expansion. For example: "Far-field records of past global sea-level highstands and coupled ice-sheet climate model results[5], suggest that partial or even total WAIS "collapse", i.e., major, rapid retreat, occurred during some warm periods of the Brunhes chron (773–0 ka). This includes the last peak interglacial of Marine Isotope Stage (MIS) 5e (130–115 ka), which is regarded as a key analogue for a near future scenario with temperatures ~1–1.5 °C above pre-industrial levels."

We thank Reviewer#1 for this excellent suggestion, which we adopted nearly in full (lines 113-119).

L. 124: I suggest expanding slightly on the type of seawater temperature proxy introduced here.

We broke the corresponding sentence apart and provide some more information about the seawater temperature proxy in the second sentence (lines 135-140).

Results

L. 199-201: Instead of stating that "down-core Ba/Rb and Br/Rb records show distinct maxima during interglacial intervals," the authors interpret these maxima as reflecting increased phytoplankton productivity in surface waters. I would argue that this interpretative component belongs in the Discussion rather than the Results, as it moves beyond description into proxy interpretation.

L206–209: This section is not purely descriptive but includes interpretative elements, which would be more appropriate for the Discussion.

L204–209: The phrase "…probably by interactions of bottom currents with the fine-grained suspension load…" represents an interpretation of depositional processes. I recommend moving this to the Discussion section.

Regarding these three comments: See our response to main issue 2) raised by Reviewer#1 and the changes we carried out in the revised manuscript to address this point.

L211–216: This is a very long sentence and is difficult to follow. I suggest breaking it up for clarity.

In response to this comment and a comment along the same lines by Reviewer#2, we carefully went through the manuscript and the Supplementary Information and split up all long sentences.

L221: Again, this sentence is quite long and could be separated for readability. For example: "…delivery of iceberg-rafted debris (IRD). The IRD was likely deposited when WAIS sectors …"

We have split this sentence and modified the text.

L293: The phrasing "…support the hypothesis that …" is interpretative and would be better placed in the Discussion rather than the Results.

See our response to the main point 2) of Reviewer#1 and the changes in the revised manuscript.

Discussion

L349: When reading this section, I wondered why the contemporary samples from Core U1533 were not included in the plot. Did these samples also exhibit Type-2 smectite? If so, their inclusion could further support these interpretations.

Pristine latest Holocene sediments could not be sampled at either Site U1533 or Site U1532 due to core-top disturbance and/or seafloor surface sediment loss (see also Figs. 3, 4). Such disturbance/sediment loss is caused by the drilling process and common for IODP drill cores. Therefore, a set of sediment cores recovered with conventional coring equipment, e.g., multiple, box, piston or gravity cores, is often recovered from a planned IODP drill site on a pre-site survey expedition. Unfortunately, no such pre-site survey cores are available from sites U1533 and U1532, and no conventional coring gear was available on RV JOIDES Resolution during IODP Exp. 379. The lack of pre-site survey cores for both Exp. 379 sites is partly due to the fact that both sites had not been alternate sites in the original drilling proposal and were only added a few months before Exp. 379 because of unusual, very heavy sea-ice cover of most proposed sites during the previous austral summer 2017/2018.

The conventional core nearest to sites U1533 and U1532 is piston core PS58/254-1 (Fig. 1), which was recovered 68 km SSE of Site U1533 in 2001 and can, to some degree, be considered as a pre-site survey core for Site U1533. The sedimentology and diatom assemblages of this core had been studied by Hillenbrand et al. (2009) and Konfirst et al. (2012). The uppermost 18 cm of sediment of core PS58/254-1 had been lost during the coring process but could be compensated for with sediment samples from multiple core PS58/254-2, which was deployed at the same site and recovered 27 to 42 cm long, small diameter (6.3 cm) cores with undisturbed seabed surface sediments (Hillenbrand et al. 2009). These sediments had been analysed by Hillenbrand et al. (2009), with Ehrmann et al. (2011) investigating the clay mineral assemblage in the contemporary seafloor surface sediments. When we started our research for this new study in 2019, however, no sediment material from multiple core PS58/254-2 or multiple core PS58/253-2, which had been collected just ca. 35 km further east, was available because it had been used up for the previous sedimentological and

provenance analyses (Hillenbrand et al. 2009, Ehrmann et al. 2011, Simões Pereira et al. 2018, Wengler et al. 2019) as well as micropalaeontological investigations (e.g., Esper & Zonneveld 2007, Esper et al. 2010). The nearest seafloor surface sediment sample available for analysing the contemporary smectite composition was therefore a sample from box core BC495, which had been collected from the same site as piston core PC494 in 2008 and lies 149 km south of Site U1533. We therefore analysed a surface sample from BC495 for smectite chemistry for our and a related study (see Fig. 2 & Park et al. 2024).

Methods:

L420: Please add a reference to Figure 1.

Done.

L468: This is a clear and helpful explanation. You may wish to quantify the temporal offset (ca. 13,000 years) to give readers a better sense of the impact of lock-in depth on the chronology.

Done.

L469: Although this is mentioned earlier, I suggest beginning with a clarifying sentence noting that this record was not analysed as part of the present study but is instead used for comparison.

Done (see also reply to comment on L508-537 by Reviewer#1).

L489–490: "…Sites U1533 and U1532 were measured at 2-cm depth resolution during IODP Exp. 379 using a whole-round…" I suggest deleting "on board".

Done.

L508–537: This paragraph describes the collection of surface samples. I recommend introducing a separate section (e.g., "Contemporary Surface Sediments") following "Sediment Cores and Age Model," which would make this information easier for readers to locate.

We thank Reviewer#1 for this very good suggestion. We rearranged the "Methods" section as follows: We relabelled the old subsection "Sediment cores and age models" with the heading "IODP Expedition 379 drill cores and age models", cut the BC495/PC494 information from the old subsection and merged it together with the information on contemporary surface sediment samples cut from subsection "Clay mineral assemblages" in the new subsection "Contemporary seafloor surface sediment samples and conventional cores". We believe this restructuring makes it easier for the reader to find the information (N.B.: As a result of this restructuring, we swapped Tables S2 and S3).

L537: This is a useful point. You could consider adding that gravity cores (and similar surface cores) may be subject to over-penetration; therefore, without surface dating (e.g., ^{210}Pb), it is not always possible to confirm that the recovered sediment is truly modern.

*We rephrased to "*This is because at those sites sedimentation rates for MIS-1 can be very low. Moreover, surface sediments recovered in piston and gravity cores can be heavily disturbed – for example, when a piston penetrates the seabed or the "soupy" seafloor surface sediments start to flow during core recovery – or may be entirely lost, when a gravity corer over-penetrates (e.g., refs. 37,73).*"(lines 566-570).*

L600: "…of the diatom species Fragilariopsis kerguelensis (F. kerguelensis)[66–68]…"

We had followed common practice here, i.e., after the first full spelling of a genus name and a species name the genus name is abbreviated in the following text. We therefore did not change this.

Figures:

Figure 1: "Amundsen Sea" is already spelled out in the figure itself and therefore does not need to be repeated in the caption. I suggest removing it from the figure text. In addition, please define the abbreviations WAIS and APIS in the caption for clarity.

Thank you for spotting this. All changed accordingly. Please note that we added other abbreviations for locations as we have added a new table (Table 1) referring to these locations.

Reviewer #2 (Remarks to the Author):

Hillenbrand et al. provide a manuscript regarding WAIS deglaciation episodes in the last 800 kiloyears, starting from MIS 19, and discuss how these events can be used to infer information about WAIS current situation and its future projections. To do so, the Authors analyze clay mineral assemblages from various sources contained in the stratigraphic sequence, coupled with a diatom-derived paleotemperature proxy and with XRF elemental ratios. All proxies point towards a systematic deglaciation and ungrounding, for all the interglacial periods before the Holocene, of the Amundsen sector of the WAIS, in conjunction with an increase in sea surface temperature evidenced by the diatom-derived proxy. The Authors conclude that the deglaciation and ice loss during interglacial periods was determined primarily by ocean forcing, with increased advection of warm CDW towards the Amundsen shelf, and that this process, coupled with ongoing anthropogenic emissions, will inevitably cause an amplified rise of the sea level.
The manuscript is well researched, well written and the data are presented in an overall exhaustive way. The methodology is sound and the entire data set used by the authors is available and easy to read. The results are solid and innovative, being extremely important to understand the evolution of the Antarctic ice sheet during the Pleistocene, especially for the community revolving around paleo-reconstruction in polar environments and for modelling future projections. My comments concern some minor modifications, mostly to enhance data visualization and to clarify certain aspects of the work, apart from a couple of secondary aspects that, in my opinion, deserve to be discussed. The manuscript has already a very valid structure and I think that, after these few adjustments, it will be ready for publication.

We appreciate these predominantly positive comments by Reviewer#2 and hope that we can adequately address their remaining concerns in the following.

GENERAL COMMENTS

1) The Authors state that the low smectite content of glacial sediments from both IODP cores can be related to a weakened poleward transport by CDW advection. I agree with the interpretation, but I would point out that inferring this from the smectite content alone could be misleading, since the content could be lower even in a scenario of increased transport of smectite by the CDW, if the sedimentation rate contemporarily increases and the mineral gets diluted in a much larger volume. From Figs. 3 and 4 it seems that the sedimentation rate generally decreases during glacial periods, thus

corroborating the authors' interpretation, but I would like this point to be, even if briefly, discussed in the text for clarity.

We thank Reviewer#2 for their thoughtful comments on these points. We had tried to address the dilution question for low kaolinite contents during interglacials (lines 251-255 in the original submission) but – in hindsight – should have clarified this for low glacial smectite contents, too. In light of this comment by Reviewer#2 and their comment on lines 251-255 (see below), we have now rearranged this subsection (lines 279-324 in the revised manuscript), thereby addressing effects of dilution and sedimentation rates (lines 302-324). In summary, we start with highlighting the presence of Type-2 smectites in past interglacial sediments, report the coinciding increase in Ti/K ratios, and then interpret this to indicate increased smectite supply from the sub-Antarctic Pacific basin. We go on and point out that the interglacial smectite increase cannot solely result from reduced kaolinite supply from the shelf because then at least the chlorite contents should also have increased. In contrast, they lack a systematic glacial-interglacial pattern. We then argue that, in turn, the prominent kaolinite increase during glacials cannot simply reflect reduced smectite supply (because of the lack of a related chlorite change and an illite increase, which is only subtle). Finally, we highlight that despite variable sedimentation rates (i) between glacials and interglacials, and (ii) between Site U1532 and Site U1533, the smectite and kaolinite contents show the same glacial-interglacial pattern at the two sites. We finish the subsection by alerting the reader to the caveat of only few smectite chemistry analyses on the glacial sediments but emphasise that the SSST-reconstructions are consistent with our interpretations from the clay mineral assemblages.

In Table S1 the Linear Sedimentation Rate is reported (actually, there are two different LSR for both cores, without a clear distinction in the column header), but it is not clear from the layout of the table which sedimentation rates are associated with glacials and which ones with interglacials. Would it be possible to plot, maybe in the SI (in Fig. S3 or in a separate panel), the sedimentation rates estimated from the age model?

We apologise for the confusion with the two linear sedimentation rates (LSRs) given for sites U1532, U1533 (and PC494) in Table S1 because we had accidentally omitted explanatory text from the table caption. For the IODP sites, the first LSR distinguishes between MIS 2, 3 and 4 (U1532, U1533) and MIS 5a-d and MIS 5e (U1533), while the second LSR gives the average values for MIS 2-4 and MIS 5, respectively. For PC494, the first LSR is based on lithostratigraphy, whereas the second LSR uses additional tie points derived from its relative magnetic palaeointensity (RPI) record. We have now added explanatory text to the Table S1 caption and also rearranged the table columns. Moreover, we have – as suggested by Reviewer#2 – added the (second) LSRs for U1532 and U1533 in Fig. S3 to highlight glacial-interglacial variability.

2) The paleo-SSST reconstruction is compelling, but one thing that is not discussed is the apparent tendency of having higher temperatures going downcore, which is, at best, mildly suspicious. I don't seem to find any mention of this trend in the manuscript, in the SI or in the cited references for the methodological approach (42 and 69).

Thank you for this observation. First, we note that this trend is not totally consistent because SSSTs at Site U1533 were lower during MIS 7 than during MIS 5 and MIS 9, but we agree that the interglacial SSSTs from MIS 7 to MIS 15 show an increasing trend with age. Interestingly, the CDW temperatures derived by Chandler & Langebroek (2024) from stacked Southern Ocean bottom-water temperatures at 6 pelagic core sites (5 sites far north of the Antarctic Polar Front and 1 site just south of it) show the same interglacial trends as the SSSTs at Site U1533 from MIS 7 to MIS 11, and also MIS 5 is characterised by higher

temperatures than MIS 7, even though the temperature differences in the CDW stack are smaller (see Fig. R1 below). This suggests that the "suspicious" SSSTs at Site U1533 are actually those from MIS 15 to MIS 13. Hillenbrand et al. (2009) reported for core PS58/254 a major shift in glacial-interglacial amplitudes of sediment parameters starting with MIS 16, which is also observed at Site U1533 (Gohl et al. 2021, p. 43 in U1533 chapter). In their detailed diatom analysis of core PS58/254, Konfirst et al. (2012) found that this major shift also coincided with a prominent increase of diatom content in interglacial sediments after MIS 16, which is also reflected by coinciding high diatom abundances at Site U1533 (Mastro 2020). Konfirst et al. (2012) attributed this change to the Amundsen Sea Low being positioned more frequently further south during interglacial periods starting with MIS 15, causing the Southern Hemisphere Westerly Winds (SHWW) to shift closer to the Amundsen Sea continental margin. The proposed SHWW shift should have increased the CDW upwelling along the Amundsen Sea continental margin (e.g., Thoma et al. 2008), forcing WAIS retreat from the shelf and raising SSSTs above the continental rise. The interglacial SSST trends from MIS 15 to MIS 5 recorded at Site U1533 may, thus, be explained by changes in the latitudinal position of the Amundsen Sea Low.

Alternatively, other factors in addition to water temperature may have affected the SSST reconstructions at Site U1533 during the earlier interglacials. Already Glemser et al. (2019) had observed that a Fragilariopsis kerguelensis population with slightly different frustule shapes prevails in the Pacific sector (Bellingshausen Sea) when compared to the F. kerguelensis population in the Atlantic sector of the Southern Ocean, for which Kloster et al. (2018) had originally developed their temperature calibration. Recently, Ruggiero et al. (2024) showed that there is also a slight difference between F. kerguelensis populations from the Pacific (Amundsen Sea) and Indian (Sabrina Coast) sectors. Thus, we cannot rule out that the F. kerguelensis population in the Amundsen Sea has slightly changed on orbital timescales (e.g., by mixing with populations from further west and east or by becoming more endemic), which may have affected our SSST reconstructions. In this context, we note that F. kerguelensis became the dominant diatom species in the Antarctic Zone only after MIS 16 (Cortese & Gersonde 2008). Furthermore, there is some evidence that also other environmental factors, such as nutrient supply (iron), may have had an impact on the morphometry of F. kerguelensis (Cortese et al. 2012, Shukla et al. 2013).

We are, however, reluctant to discuss all these points in our paper because: (i) we think that we will need at least one more SSST record from the Amundsen Sea that spans the last 800 kyr and shows the same long-term trend as U1533 in order to demonstrate that this trend is both robust and regional rather than local (see also response below on SSSTs during MIS 5), (ii) currently, the explanations given above are not more than speculation, and (iii) such a discussion would unnecessarily distract the reader from the main focus of our study.

REDACTED

Fig. R1: Circumpolar Deep Water (CDW) temperature anomaly during the last 800 kyr (black line) reconstructed from bottom-water temperatures at six core sites mainly from the sub-Antarctic Southern Ocean (modified from Chandler & Langebroek 2024).

I am also curious about SSST reconstruction results from the glacial periods: are they possible or the poor diatom content makes it unfeasible?

Because of the extremely low diatom content and poor preservation of the diatom frustules in the glacial sediments on the Amundsen Sea continental rise (Mastro 2020; Konfirst et al. 2012; see Fig. R2 below), it was only possible to reconstruct SSSTs for some glacial periods at Site U1533 in addition to MIS 14. These are MIS 4 (+0.8 °C), MIS 6 (+0.8 °C) and MIS 8 (+1.4 °C) (Mastro 2020). However, significant uncertainties affect these glacial SSSTs, which prevented us from reporting them: 1) Poor diatom frustule preservation during glacials may have affected the rectangularity measurements. 2) Konfirst et al. (2012) found at site PS58/254 that the diatom assemblages of glacial sediments contain significant amounts of fossil diatom species reworked from stratigraphically older sediments (dating back to the Miocene) in addition to the extant diatom taxa. This indicates that at least a proportion of the F. kerguelensis *diatoms present in the glacial sediments were reworked from (slightly) older strata as well, probably by the intense subglacial erosion on the Amundsen Sea shelf during glacial periods. As it is not possible to distinguish the reworked from the "in situ"* F. kerguelensis *frustules in the glacial sediments at Site U1533, we refrain from presenting and discussing the uncertain glacial SSSTs.*

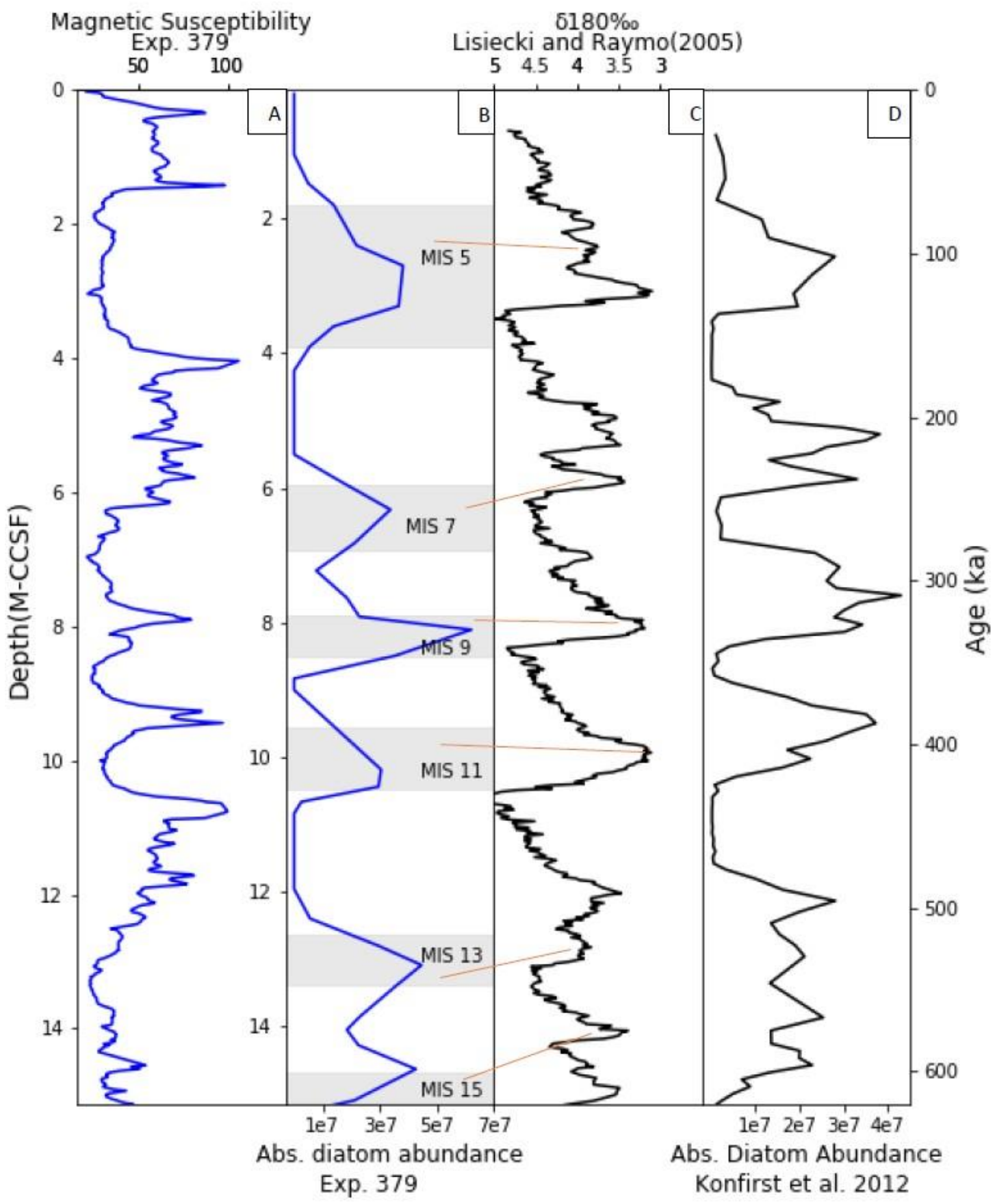

Fig. R2: Magnetic susceptibility alongside absolute diatom abundance at Site U1533 (plotted vs. depth) and the LR04 benthic foraminifera $\delta^{18}O$ stack alongside the absolute diatom abundance in piston core PS58/254-1 (plotted vs. age) for the last 800 kyr (Figure from Mastro 2020; data for PS58/254-1 from Konfirst et al. 2012).

Also, is there any way to have SSSTs for different intervals inside the same interglacial? I would expect temperatures to be higher (and thus higher CDW advection) at the end of the glacial/at the onset of the interglacial (see for example the recent work from

Pambianco et al., 2025 (Ref. 1)), and it would be interesting to compare these temperatures with other independent SST reconstructions to see if they match.

We agree that it would be very interesting to compare our SSSTs to surface ocean or even bottom-water temperature reconstructions for the LGM-Holocene transition from the Amundsen Sea (new paper by Mawbey et al. 2026, now cited) and other West Antarctic sectors, such as the Ross Sea (thank you for alerting us to the paper by Pambianco et al. 2025, which we now cite in the revised manuscript). However, apart from the problem of comparing temperatures from different parts of the water column measured with different proxies (Mg/Ca in Mawbey et al. 2026, organic biomarker RI-OH' in Pambianco et al. 2025), the largest issue is the unavailability of F. kerguelensis-*derived SSSTs for this time span from the Amundsen Sea continental rise (Fig. R2). This is mainly caused by the unavailability of pristine Holocene sediments at the IODP Exp. 379 sites and in nearby core PS58/254 because of coring disturbance/surface sediment loss (see also our response to the comment on the "Discussion" section by Reviewer#1) and partly due to the low sedimentation rates during this time interval (~2 cm/kyr, see revised Fig. S3 & Table S1). Given the still relatively low diatom abundance even in the interglacial sediments at Site U1533, the* F. kerguelensis *morphometric analyses from multiple samples taken from the same interglacial MIS had to be combined to get as many measurements as possible, and even then, it was not always possible to get close to a statistically robust number of 200 (we have now added these numbers to Table S5).*

The only exception was MIS 5, when sedimentation rates at Site U1533 were high (Fig. S3 & Table S1), allowing Ruggiero et al. (2024) to reconstruct SSSTs throughout an interglacial period (see Fig. R3 below). As expected by Reviewer#2, the highest SSSTs occur early in the interglacial. This timing is similar to that in the LGM-Holocene shelf records from the West Antarctic shelf (Pambianco et al. 2025, Mawbey et al. 2026), where the highest water temperatures were also reached during the glacial termination and early in the interglacial, respectively. However, Ruggiero et al. (2024) also reconstructed SSSTs for MIS 5 in piston core PC496 from the Amundsen Sea continental rise, where sedimentation rates were also high during MIS 5 (Horrocks 2020) and which is positioned 108 km SE of Site U1533. In this core, which is located more proximal to the WAIS margin, the highest SSSTs are recorded during the middle/later part of MIS 5 (Fig. R3), suggesting asynchrony of interglacial SSST maxima in the same Antarctic sector (possibly related to a shift in the latitude of maximum CDW upwelling). In none of these two Amundsen Sea cores, however, the SSST maxima during MIS 5 are much higher than the temperatures during the rest of this interglacial.

REDACTED

Fig. R3: SSSTs reconstructed for MIS 5 at sites U1533 (above) and PC496 (below) using the temperature model of Kloster et al. (2018). SSSTs are plotted vs. depth in centimetres below seafloor (cm b.s.f.) (figure modified from Ruggiero et al. 2024).

3) The clay mineral assemblages are the core of the work, but it is sometimes difficult for the reader to follow all the sources and associations for the different minerals and elemental compositions. I suggest implementing a brief explanation/list, maybe in a table, to sum up these details so that one can use it as a reference during the reading.

Thank you for this useful suggestion. We compiled a new Table 1 presenting the average clay mineral compositions for the various source sectors along the eastern Pacific margin of West Antarctica (Western Antarctic Peninsula, Bellingshausen and Amundsen seas), including their sub-sectors, the Sub-Antarctic SE Pacific and interior West Antarctica (basal Byrd Ice Core debris). The contemporary samples used for this characterisation are marked in Table S3. Both the Esquevin Index and the smectite composition had only been analysed on some contemporary samples from the Amundsen Sea shelf, and we added this information for its sub-sectors. Locality names in Table 1 that had not been mentioned in the original manuscript version were added to the maps of Figs. 1 and S1.

SPECIFIC COMMENTS

Lines 97-98: "This melting increases discharge of grounded ice feeding into and buttressed by the ice shelves". Please expand on this concept, the sentence is not very clear as it is.

*We rephrased this sentence accordingly (lines 99-102): "*The melting causes thinning of the ice shelves, weakening their buttressing effect, which increases the flow of grounded ice feeding into them. This, in turn, initiates glacier thinning upstream of the ice shelves and grounding-line retreat[2-4].*"*

Lines 103-108: I would rephrase as: Here, potentially irreversible ice loss from two major outlets draining into the eastern Amundsen Sea Embayment (Thwaites and Pine Island glaciers, Fig. 1) may initiate not only widespread deglaciation in this sector but also in other marine-based WAIS sectors and in the entire WAIS within the coming centuries, raising global sea-level by 3.3–5.3 metres.

In order to shorten sentences that the referees often considered as overly long, we rephrased this text as follows (lines 106-112): " Present WAIS melting is focused in the Amundsen Sea sector[2-4], where Thwaites and Pine Island glaciers (Fig. 1) drain one third of the WAIS into the eastern Amundsen Sea Embayment. Potentially irreversible ice loss from these two major outlets may initiate not only widespread deglaciation in the Amundsen Sea drainage sector but also other marine-based WAIS sectors and even the entire WAIS within the coming centuries[6,7]. This would raise global sea-level by 3.3–5.3 metres[1].*"*

Line 112: Change "last peak interglacial" with "last interglacial peak"

Done.

Line 134: "sediments from its margin". Add "continental" margin.

Done.

Lines 195-196: References are missing for these ratios (they are present in the method section, but they should be cited here as well).

We have now added references for the use of normalised barium contents as productivity proxies in this sentence. Moreover, we have inserted a sentence immediately afterwards making the reader aware of the detailed studies by Hillenbrand et al. (2009) and Konfirst et al. (2012) on nearby core PS58/254 from Resolution Drift, which previously showed that the barium proxy and sediment density relate to diatom abundance and opal content,

respectively. Finally, we clarify in the following sentence that Hopkins et al. (2024) had also used the Ba/Rb ratio as a productivity proxy for Site U1533.

Also, why Br was not normalized with Cl (Ref. 2)?

Referee#2 raises an interesting – and tricky – point here. The review chapter by Rothwell & Croudace (2015; = chapter 2 in Reference 2 cited by Reviewer#2) provides a great overview over the elements and element ratios that had been measured by XRF scanning and were previously used as palaeo-environmental (or diagenesis) proxies on sediment cores. Rothwell & Croudace (2015) explain that the Br/Cl ratio was used as a palaeo-productivity proxy by Thomson et al. (2006) in their analysis of a sequence of biogenic sediments consisting of alternating C_{org}- & sulphide-rich beds ('sapropels') and clayey nannofossil ooze beds ('marls'). Marked Br enrichments had been reported for sapropels previously, and these were partly caused by the enrichment of Br with C_{org} and partly by the higher porosity and, hence, higher sea-salt content in the pore waters of the sapropels. The latter factor is also responsible for relatively high Cl contents. Thomson et al. (2006) chose to normalise the Br counts with Cl "to reveal the presence of additional Br associated with C_{org} in excess of the constant Br/Cl sea-salt ratio"*. Rothwell & Croudace (2015) additionally refer to studies which either presented Br counts without normalisation as palaeo-productivity proxy (Caley et al. 2011; cf. Smith et al. 2017) or chose detrital elements to correct the Br data (e.g., Agnihotri et al. 2008, who used the Br/Ti ratio). In a previous investigation of sediment cores from the West Antarctic continental margin (Hillenbrand et al. 2021), we had chosen the Br/Al ratio for the same purpose.*

For our study on the IODP Exp.379 cores, we followed a similar approach but chose Rb as detrital normaliser because Rb is enriched in clay minerals and, thus, seems the most appropriate detrital normaliser for the clay-rich sediments from sites U1532 and U1533. A problem with using the Br/Cl ratio we had come across during our research for the 2021 paper was that this ratio showed no significant down-core trend, although a range of other productivity proxies (e.g., diatom content, Ba/Al ratios, $CaCO_3$ content) clearly indicated higher palaeo-productivity for (interglacial) sediment horizons (Fig. R4). These interglacial horizons were also characterised by higher Br counts but not necessarily higher Cl counts. This decoupling between Br and Cl counts suggested that the higher Br counts were due to both higher organic matter content and higher pore water (and, thus, salt) content. The Br/Cl ratio plot, however, did not reflect this because the Cl counts were much higher than the Br counts (Fig. R4). The interglacial sediments at sites U1532 and especially U1533 are much more enriched in diatoms and have, therefore, much higher porosity than the cores we had studied for the 2021 paper. This causes clear enrichments in both Cl and Br counts in the corresponding sediments, with the Br/Cl ratio, however, showing no clear down-core trend (Fig. R4). As we are unable to prove for the diatom-enriched interglacial sediments at sites U1532 and U1533 that their Br enrichments are not only caused by higher pore water but also higher C_{org} contents, we decided not to rely on Br as productivity proxy and deleted the Br/Rb ratios from the revised Figs. 3 and 4.

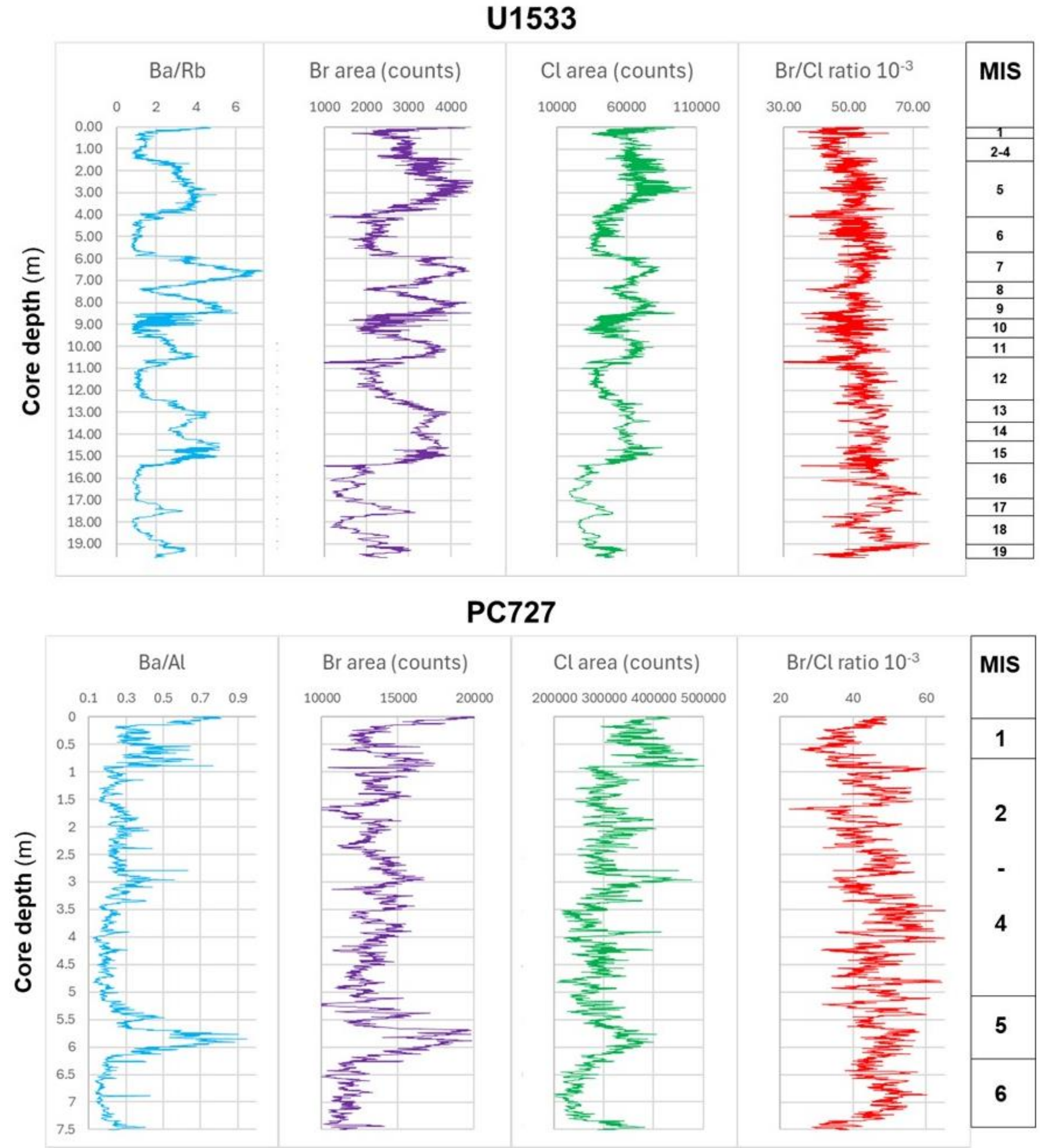

Fig. R4: Upper panel: XRF scanner data for U1533 showing that both Br and Cl counts are higher in the diatom-enriched interglacial sediments (marked by high Ba/Rb ratios), but without the Br/Cl ratios displaying the same pattern. Lower Panel: XRF scanner data for PC727 from the Bellingshausen Sea continental rise (full data set and age model for the core were published in Hillenbrand et al. 2021). Br counts are higher in the diatom-bearing sediments of interglacial MIS 1 and 5, for which Ba/Al ratios (and other proxies) indicate high productivity, but this is only partly reflected by the Cl counts and not at all by the Br/Cl ratios. In both cases the Cl counts are significantly higher than the Br counts (in PC727 Cl counts are more than an order of magnitude higher than the Br counts).

Lines 211-216: This sentence is very long. I suggest dividing it in two parts, for example: "This disparity most likely results from the dissimilar locations of the two sites on Resolution Drift. The relative contributions of different sediment transport processes affecting drift deposition throughout late Pleistocene glacial-interglacial cycles are known to vary systematically across Antarctic rise drifts, creating distinct, localized depositional settings."

Done.

Lines 251-255: Very long sentence. I suggest dividing it in two parts, for example: "The prominent kaolinite reduction in the interglacial sediments on the eastern Amundsen Sea rise does not solely result from reduced deposition of kaolinite enriched glacigenic detritus from the Amundsen Sea shelf. In this case, a corresponding prominent increase of both illite and chlorite is to be expected but is not observed".

Besides, this first sentence introduces kaolinite, and then the focus of the paragraph shifts abruptly towards smectite. I would move this part at the end of the paragraph (line 277), after the explanation regarding the high kaolinite concentrations during glacial times.

Sentence & subsection changed. See our response above regarding dilution and sedimentation rates.

Line 260: PC494 is missing from the map. If I understood this correctly, it should be located on the same site as BC495, but this is not clarified in the figure's caption. I would nonetheless suggest inserting the name of the core on the map.

In accordance with a Reviewer#1 suggestion, we use now PC494 throughout the main text and in the figures.

Line 265: Move "exclusively" after "to contain".

Done.

Line 294: Rephrase "smectites in the interglacial rise sediments" as "smectites in the Amundsen Sea rise interglacial sediments" or just "smectites in the interglacial sediments".

Changed (here and throughout).

Lines 328-334: Very long sentence. I suggest dividing it, for example "At Site U1533 the Esquevin index of illite in the pre-Holocene interglacial sediments mainly ranges from 0.30–0.40 (Fig. 3), revealing that this illite – together with other detritus – was predominantly derived from the Thurston Island block rather than Marie Byrd Land. There, the Esquevin index is typically ≤0.3029 333, and, thus, corroborates continued glacial erosion in the Thurston Island block area"

Changed.

Lines 340-341: This assumption is a bit stretched. It is true that site PS2551-1 is further west from the IODP sites, but a transport from eastern Marie Byrd Land dome towards NW/WNW would miss site PS2551-1, which is located almost straight up north to Marie Byrd Land.

*Thanks for spotting this typo. We corrected to "*NNW- to NW-directed*" (line 395). With "Marie Byrd Land dome" we refer to the area at and above sea level (e.g., Spiegel et al. 2016), whose eastern boundary is Bear Peninsula at ~111°W, whilst site PS2551 lies at 117°W.*

Lines 397-399: Please further elaborate on this concept or refer to the explanation in the SI.

*As we refer to the SI in the previous sentence and had mentioned the genetic evidence already in the previous section, we modified the sentence in that section (lines 415-418) to "*For MIS-5e, our data indicate maximum WAIS retreat at a relatively late stage (Fig. 3), in support of published genetic evidence pointing to the opening of a Trans-Antarctic seaway as the result of a WAIS collapse late during the last interglacial[20] (Supplementary Information).*"*

Lines 406-411: This sentence is a bit convoluted, I would suggest dividing it in two sentences or to shorten it to make it clearer.

Done.

Line 420: Remove the initial "while" and close the sentence at row 423 after "90%" with a dot.

Done.

Line 534: Add a comma after "sediments".

N/A (the original sentence has been split in two).

Line 548: Change "they were" with "being".

Done.

Line 908: Amundsen Sea is already spelled out on the map. WAIS and APIS are missing from the caption.

Done.

Line 950: The EI is not plotted on the Zr/Rb ratio but on the Ti/K ratio.

The figure has been changed (EI is shown in its own panel).

Line 973: See previous comment.

The figure has been changed (EI is shown in its own panel).

FIGURES

The figures are overall quite clear and understandable, but I would suggest making some improvements in order to enhance the representation of the dataset.

Figure 1

- Names of the seas and geographic indications: the sea names are aligned and justified in different ways. I think this partially impedes the readability of the figure. In order to make the figure tidier and easier to read, I suggest arranging the names in a more symmetrical way. The Patagonia name is divided, it would be tidier to leave it on one row, if possible.

All changed accordingly.

- Core names and locations: core names and symbols are not well readable, they are significantly smaller than geographic locations and often black on a dark blue background. I would suggest changing the colors (e.g. from black to white) and/or add an outline. You could also add a third panel with a focus on the Amundsen Sea sector and embayment, where most of the cores are located, showing the cores and the main locations (e.g. Amundsen Rise, etc), without the surface sediments if this helps readability. This panel could be also useful to graphically show, with arrows, the preferential transport paths described in the main text, which would help the reader following the main discussion.

We adopted the first suggestion and modified core IDs & symbols in Figs. 1 and S1. Furthermore, we added the location of the Byrd Ice Core with the average clay mineral composition of its englacial basal debris (new Table 1). We did not add a new figure focussing on the Amundsen Sea sector because 1) high resolution bathymetric data from its continental rise are patchy, i.e. the reader would not see much more detail than by zooming into Figs. 1 and S1 (cf. Fig. 1 in Gohl et al. 2023, which we show in Fig. R5 below), and 2) actually three new figure panels would be required to illustrate the transport of detritus during a) the present, b) glacial periods and c) past interglacial periods when most grounded ice in this drainage sector was lost. To be meaningful, the latter panel would require the illustration of WAIS extent (i.e., the extent of grounded ice and ideally also the extent of

floating ice shelves). We are reluctant to do this because – as we clarify in the paper – our data show major retreat and ungrounding of Thwaites Glacier, Pine Island Glacier and other ice streams draining into the Amundsen Sea Embayment but also indicate that small ice caps remained on the Marie Byrd Land dome and Thurston Island block. We cannot constrain the exact ice extent in these areas above sea level. Even more crucially, our data are unable to verify ice-sheet models which conclude that loss of grounded ice in the Thwaites Glacier and Pine Island Glacier catchments during past interglacials led to collapse of the entire WAIS (e.g., DeConto & Pollard 2016) or reconstruct ice loss in just several but not all of the WAIS drainage sectors (e.g., Golledge et al. 2021). Our data indicate loss of the marine-based ice in the Amundsen Sea drainage sector but retreat in the neighbouring WAIS catchments (which such a new figure would have to illustrate) would be highly speculative. Instead, we now briefly describe in the "Discussion" section the wide range of different WAIS configurations reconstructed for the last interglacial period by previous modelling studies (lines 367-372), so that readers interested in details can look up the modelled WAIS extent in the literature we refer to.

REDACTED

Fig. R5: Bathymetric map of the Amundsen Sea sector based on a compilation of multi- and single-beam bathymetric data (from Gohl et al. 2023).

- Symbology: you use different symbols and colors corresponding to different % of minerals. The brown triangles are often not well distinguishable on the blue background. You may use the same symbol for all the categories, with symbol size proportional to the % (e.g. small star for low %, big star for high %, in-between star size for in-between %). Even better, it would be ideal to couple this graphic representation with a color palette (e.g. reddish-white for low %, red for high %, in-between shades for in-between %), ideally choosing a different color palette for each mineral.

We added a white outline to the brown triangles to increase their visibility. However, we did not apply other changes because we had tried several ways to display the various clay mineral content ranges and strongly believe that the way how we did it, is the most suitable

(also for readers with colour vision deficiency). For example, using different symbol sizes leads to too much overlap of sampling sites (many of the sites would be invisible), while using the same colour in different shading for a clay mineral would make it very hard to recognise even significant differences in its content. We agree that our approach is to some degree a compromise, but so would be any other, too.

Figure 2

It is challenging to read this diagram in its entirety. Firstly, I would suggest moving type1 and type2 arrows on the right axis (Mg) and separating the legend, listing the glacial samples under panel 2b. The colours for BC27, BC480 and BC502 contour lines are kind of similar, and it is not easy to distinguish them. Also, looking at the relatively consistent compositions of contemporary samples (excluding sub-Antarctic SE Pacific Basin samples), it could be more reader-friendly to plot an "average" contour line from all sources of Type 1 smectites.

We moved the arrows to the right axis (thanks for this good idea), separated the legends for the two panels of the figure, and marked the outline of the smectite compositions at sites BC36 (Thwaites Glacier) and BC27 (Pine Island Glacier) by a dotted and a dashed contour line, respectively, so that they can be easier distinguished. We did not merge the shelf and slope samples to display them with a single Type-1 contour line, however, because we think it is important to highlight for the reader the similar compositions of smectites in the glacigenic detritus delivered from the different sources around the eastern Amundsen Sea Embayment.

Figures 3 and 4

The figures are simple yet clear. It would have been nice to report the core photos somewhere, i.e. in the supplementary material.

*Thanks for the compliment. Like for all IODP expeditions, the core photos and other ship-board data for U1532 and U1533 are available from IODP. For clarification, we have added the following sentence to the "Data availability" section (lines 719-721): "*The bulk of the shipboard-collected core data from IODP Exp. 379 (including core photos) is accessible at https://zenodo.org/communities/iodp (for a list of available datasets, see https://publications.iodp.org/proceedings/379/datasets.html).*".*

References

1. Chiara Pambianco et al., Past intrusion of Circumpolar Deep Water in the Ross Sea: Impacts on the ancient Ross Ice Shelf. Sci. Adv. 11, eadt7075(2025). DOI: 10.1126/sciadv.adt7075

2. Croudace, I.W., Rothwell, R.G. (Eds.), 2015. Micro-XRF Studies of Sediment Cores: Applications of a non-destructive tool for the environmental sciences, Developments in Paleoenvironmental Research. Springer Netherlands, Dordrecht. https://doi.org/10.1007/978-94-017-9849-5

We added the 1st reference but not the 2nd as we prefer to cite original papers for XRF element measurements instead, with some of those not exclusively being based on XRF scanning.

Reviewer #3 (Remarks to the Author):

I co-reviewed this manuscript with one of the reviewers who provided the listed reports. This is part of the Nature Communications initiative to facilitate training in peer review and to provide appropriate recognition for Early Career Researchers who co-review manuscripts.

Thank you for all the constructive input.

Reviewer #4 (Remarks to the Author):

The manuscript of Hillenbrand and co-authors presents a robust and multidisciplinary dataset (XRF, clay mineralogy, magnetic susceptibility, and bulk density) aimed at reconstructing the dynamics of West Antarctic Ice Sheet (WAIS) retreat during the glacial-interglacial period of the last 800 kyr. The dataset is solid and carefully constructed, and I commend the authors for compiling and presenting it. In addition, the integration with modern datasets is particularly strong and significantly enhances the overall impact of the study.
The multiproxy approach convincingly highlights clear differences between glacial and interglacial patterns, and the link to oceanographic processes is well developed and compelling. The scientific concept is strong: the manuscript not only reconstructs past changes but also provides meaningful implications for future WAIS behaviour under ongoing climate warming. Overall, these aspects make the study suitable for publication in a journal such as *Nature Communications*. I particularly appreciated the Discussion section, especially the connection drawn between past dynamics and future scenarios. I thank the authors for this contribution, which I believe will be valuable to the scientific community.

We thank Reviewer#4 for this positive evaluation.

However, some aspects of the manuscript should be improved before acceptance.

The main issue observed throughout both the Introduction and the Results sections, concerns writing style and structure. Many sentences are very long and occasionally difficult to follow. This is not a problem of scientific content, which is strong, but rather of how the results are communicated. I encourage the authors to revise the text for clarity and conciseness.

As this issue had also been raised by the other referees, we systematically went through the text of both the main manuscript and the Supplementary Information, shortened long sentences or split them up, made the text more concise where possible, and added more clarification where needed.

In addition, the distinction between Results and Discussion is sometimes confused. While some degree of overlap is understandable in this type of article, parts of the Results section appear overly interpretative. I recommend reducing the level of interpretation in the Results and reserving more speculative or integrative statements for the Discussion.

As outlined above in our response to the similar 2nd main comment by Reviewer#1, we followed a suggestion by this referee and combined "Results and Interpretation". This allowed us to avoid text duplication and keep the focus in the "Discussion" section on the nature and forcing of past WAIS changes. Given the related comments by all reviewers, we apparently had not made it clear enough in the original submission that several of the interpretations in the "Results" section are based on previous publications (e.g., use of the Ba/Rb ratio at Site U1533 as palaeo-productivity proxy, depositional processes on Resolution Drift, etc.). We have tried to clarify this in the revised manuscript version. Finally, we like to emphasise that we did not - and do not - intend to "sell" the earlier published interpretations (which we agree with) as our own and take credit away from the authors of these original studies.

These comments are intended as constructive suggestions to improve clarity and readability. Importantly, they do not detract from the overall quality and significance of the work, which I consider to be high.
For these reasons, I recommend minor revision.

We are grateful to Reviewer#4 for their constructive and thoughtful comments.

Specific comment:
Introduction
The introduction provides a clear and relevant overview of the vulnerability of the West Antarctic Ice Sheet (WAIS) and effectively frames the importance of understanding its response to past and future climate change. The authors successfully highlight the role of ocean forcing, particularly Circumpolar Deep Water (CDW), and place the study within the broader context of sea-level rise and interglacial variability. The scientific motivation is therefore well established.
However, several aspects of the Introduction could be improved to enhance clarity and readability. The main issue concerns sentence structure: many sentences are overly long and contain multiple layers of information (e.g., lines 95-97;109-113), which makes the text at times difficult to follow. I recommend revising these passages to improve conciseness and flow, for example by splitting complex sentences and simplifying phrasing where possible.

Reviewer#4 provides here an example for one of the main issues they raised, and we refer to our response above.

In addition, the transition from the general background to the specific knowledge gap could be made sharper (See lines 109 -118). While the Introduction discusses uncertainties in past WAIS behaviour, the exact gap that this study aims to address is not always stated as explicitly as it could be. Strengthening this point would help to better position the novelty and relevance of the work.
Finally, the concluding paragraph of the Introduction could be made more direct and impactful. The current formulation is somewhat descriptive (e.g., "Here we investigate…"), whereas a more assertive statement of the study's objectives and approach would improve the overall framing.
Overall, the Introduction is scientifically sound and well contextualized, but would benefit from stylistic refinement and a clearer articulation of the research gap.

We have modified the "Introduction" section accordingly to clarify (i) the existing knowledge gaps (lines 119-130) and (ii) the purpose of our study (lines 131-143).

Results

Clay mineral provenance of Antarctic marine sediment: Fingerprinting source areas
The first part of the Results provides important information for the subsequent interpretations. The compilation of modern and previously published datasets is particularly valuable, and the identification of kaolinite as a key tracer for the Amundsen Sea sector is well motivated. The integration of mineralogical and geochemical data represents a clear strength of the study.

Thank you for this positive comment.

However, this section would benefit from several structural and stylistic improvements. A substantial portion of the text is devoted to general background on Antarctic weathering processes and clay mineral formation, which partly repeats information that is already expected to be known by the readership or would be better suited to the

Introduction. I recommend reducing this descriptive component and focusing more directly on the novel results of the study. For example, in my opinion, lines 131–143 could be removed or significantly shortened and incorporated into the Introduction.

*We had deliberately placed this background text at the beginning of the subsection, which reports the clay mineral fingerprints of the source areas within and around our study area. We did this because we (i) do not expect that the broad readership of a "*Nature Communications*" article is aware of this information already, and (ii) believe that this background provides important context for the geographical distribution of the clay minerals in the contemporary seafloor surface sediments. Placing this background information into the general "Introduction" section would have separated it from the data, for which it provides the context, so we did not change this.*

As in the Introduction, several sentences are too long and contain different concepts, which makes the text difficult to follow. I suggest rephrasing sentence structure and improving conciseness would greatly enhance readability.

Overall, this section is robust and clearly relevant, but it would benefit from a clearer distinction between background and new results, improved readability, and a more balanced presentation of interpretations.

We have rephrased the related sentences and tried to improve readability.

Major glacial-interglacial changes in WAIS extent are archived in clay mineral provenance data from the Amundsen Sea continental rise
This section presents a detailed and well-supported description of glacial–interglacial variability in sedimentary facies, physical properties, and clay mineral assemblages. The multiproxy dataset provides a coherent picture of environmental change across glacial–interglacial cycles.
In particular, the identification of variability between bioturbated diatom-rich intervals and laminated terrigenous units, as well as their linkage to interglacial and glacial conditions, is convincing and in agreement with previous studies from the Amundsen Sea margin. The integration of geochemical proxies (e.g., XRF ratios) further strengthens the interpretation of productivity changes and sedimentary environments.

Thank you for the positive evaluation of this section.

Remains unclear the interpretations the turbidity current (lines 206-211) are presented with a relatively high degree of confidence, despite being indirectly inferred. While these interpretations are plausible, they should be expressed more cautiously unless supported by additional independent evidence.
Finally, as in other parts of the manuscript, several sentences are overly long and combine multiple concepts, which reduces readability. Simplifying sentence structure and separating observational results from interpretative statements would significantly improve clarity.

The turbidity current interpretations are based on the cited IODP Exp. 379 publications as well as earlier published literature on geophysical data and conventional sediment cores from the Amundsen Sea continental rise. The deep-sea channel flanking Resolution Drift (text in lines 229-232 & 494-498 and Fig. R6 below) provides clear evidence for the activity of turbidity currents in close proximity of the core sites, and we think our text describing the transport and depositional processes was cautious enough (use of "likely" and "probably"). Elaborating on these processes further is beyond the scope of our paper, but we like to point out to Reviewer#4 that Benedict Reinardy, one of the co-authors of our study, is currently

preparing a manuscript presenting independent evidence from micromorphology analysis for turbidite deposition at the IODP Exp. 379 sites.

Discussion:

I found the Discussion to be of high quality and very engaging. I particularly appreciated the final "Global implications" section, which significantly strengthens the impact of the manuscript. Thank you to the authors for this thoughtful addition. The glacial–interglacial variability is well supported by the multiproxy approach and provides a coherent picture of environmental changes across glacial–interglacial cycles.

We are pleased that Reviewer#4 apparently likes the "Discussion" section.

I have one point that I found unclear. In lines 314–317, the authors attribute the observed changes in clay mineral assemblages primarily to a cessation of subglacial erosion. However, it is not fully clear why alternative mechanisms are not considered or discussed. Could dilution effects or changes in sediment supply and transport pathways also contribute to the observed signal? A brief discussion of potential alternatives would strengthen the interpretation's robustness.

*See our response to the related comment on dilution by Reviewer#2. The only alternative explanation for the observed interglacial kaolinite minima and coinciding smectite maxima is enhanced interglacial supply of smectite enriched detritus from either the Ross Sea sector or the Bellingshausen Sea sector by bottom currents. We had discussed (and dismissed) the former possibility in the Supplementary Information and now clarify this point at the beginning of subsection "*Late Pleistocene variability in ocean forcing…*" (lines 279-285).*

Figure

The figures are well prepared, clear, and of high quality, and I have no major comments on them.

We are grateful that the figures are clear.

My only observation, which I understand the authors may choose not to address, concerns the lack of a detailed bathymetric map or geophysical image showing the precise location of the cores. While not essential, including such a figure could further improve the manuscript and better contextualize the study area.

See our response to the recommendation by Reviewer#2 of including an additional map focussing on the Amundsen Sea. Unfortunately, high resolution bathymetric data from the Amundsen Sea continental rise are patchy, i.e. the reader would not see much more detail than by zooming into Figs. 1 and S1 (cf. Fig. 1 in Gohl et al. 2023, see Fig. R5 above). Even the best bathymetric map available from the IODP Exp. 379 sites, which was published as Fig. S1 by Gohl et al. (2023) and is shown as Fig R6 below, had to combine single- and multi-beam data of very different resolution and quality, and therefore provides only limited detail.

REDACTED

Fig. R6: Bathymetric map of the vicinity of the IODP Exp. 379 sites based on multi- and single-beam bathymetry data (figure from Gohl et al. 2023).

Material and methods

Are clear and support the data.

Supplementary materials are clear and supportive of the draft and not excessively long.

Thank you.

Additional changes:

We corrected typos and incorrect grammar as well as a minor error in the characterisation of the Bellingshausen Sea source area (a continental slope sample instead of a nearby continental shelf sample had been included by mistake in the original submission – now corrected in Table S2 and Fig. S2 and taken into account for the new Table 1). Furthermore, we made very minor modifications to the text for clarification and added in both the revised manuscript and the Supplementary Information references in response to the reviews and references to new, highly relevant papers (e.g., Bentley & Wolff 2026, Carter et al. 2026, Mawbey et al. 2026).

References cited only in this rebuttal (but not in the main paper or the Supplementary Information):

Agnihotri, R., Altabet, M.A., Herbert, T.D. & Tierney, J.E. (2008): Subdecadally resolved paleoceanography of the Peru margin during the last two millennia. Geochem. Geophys. Geosys. ***9****, Q05013, doi:10.1029/2007GC001744.*

Caley, T. et al. (2011): New Arabian Sea records help decipher orbital timing of Indo-Asian monsoon. Earth Planet. Sci. Lett. ***308****, 433-444.*

Cortese, G. & Gersonde, R. (2008): Plio/Pleistocene changes in the main biogenic silica carrier in the Southern Ocean, Atlantic Sector. Mar. Geol. ***252****, 100-110.*

Cortese, G., Geronde, R. Maschner, K. & Medley, P. (2012): Glacial-interglacial size variability in the diatom Fragilariopsis kerguelensis*: Possible iron/dust controls?* Paleoceanogr. ***27****, PA1208, doi:10.1029/2011PA002187.*

Esper, O. & Zonneveld, K.A.F. (2007): The potential of organic-walled dinoflagellate cysts for the reconstruction of past sea-surface conditions in the Southern Ocean. Mar. Micropal. ***65****, 185-212.*

Esper, O., Gersonde, R. & Kadagies, N (2010): Diatom distribution in southeastern Pacific surface sediments and their relationship to modern environmental variables. Palaeogeogr. Palaeoclimatol. Palaeoecol. ***287****, 1-27.*

Rothwell, R.G. & Croudace, I.W. (2015): Twenty years of XRF core scanning marine sediments: What do geochemical proxies tell us?. In: Croudace, I.W. & Rothwell, R.G. (Eds.), Micro-XRF Studies of Sediment Cores. Dev. Paleoenvironmental Res. ***17****, pp. 25-102.*

Shukla, S.K., Cortese, G., Corsta, X. & Nayak, G.N. (2013): Climate mediated size variability of diatom Fragilariopsis kerguelensis *in the Southern Ocean.* Quat. Sci. Rev. ***69****, 49-58.*

Spiegel, C. et al. (2016): Tectonomorphic evolution of Marie Byrd Land – Implications for Cenozoic rifting activity and onset of West Antarctic glaciation. Global Planet. Change ***145****, 98-115.*

Thoma, M., Jenkins, A., Holland, D. & Jacobs, S. (2008): Modelling Circumpolar Deep Water intrusions on the Amundsen Sea continental shelf, Antarctica. Geophys. Res. Lett. ***35****, L18602, doi:10.1029/2008GL034939.*

Thomson, J., Croudace, I.W. & Rothwell, R.G. (2008): A geochemical application of the ITRAX scanner to a sediment core containing eastern Mediterranean sapropel units. In: Rothwell, R.G. (Ed.), New Techniques in Sediment Core Analysis. Geol. Soc. London Special Publ. ***267****, 65-77.*

Wengler, M. et al. (2019): A geochemical approach to reconstruct modern dust fluxes and sources to the South Pacific. Geochim. Cosmochim. Acta ***264****, 205-223.*

Response to reviewer comments on the revised version of manuscript NCOMMS-26-010571 “Repeated loss of the West Antarctic Ice Sheet’s Amundsen Sea sector during late Pleistocene interglacials“submitted by C.-D. Hillenbrand et al. to *Nature Communications*

In the following we give the reviewer comments in bold font and provide our response in italics.

Reviewer #1 (Remarks to the Author):

The authors have addressed all the comments and suggestions, and I find the manuscript to be ready for submission. I have no further comments.

Reviewer #2 (Remarks to the Author):

The Authors adequately addressed all requests and scientific questions, providing an exhaustive response. As for me, the paper is now ready for publication.

Reviewer #3 (Remarks to the Author):

I co-reviewed this manuscript with one of the reviewers who provided the listed reports. This is part of the Nature Communications initiative to facilitate training in peer review and to provide appropriate recognition for Early Career Researchers who co-review manuscripts.

We thank the referees for their thoughtful and constructive reviews of the original manuscript version, which helped to improve our paper.
